# Significant, but not biologically relevant: *Nosema ceranae* infections and winter losses of honey bee colonies

**DOI:** 10.1038/s42003-023-04587-7

**Published:** 2023-03-01

**Authors:** Vivian Schüler, Yuk-Chien Liu, Sebastian Gisder, Lennart Horchler, Detlef Groth, Elke Genersch

**Affiliations:** 1 Institute for Bee Research, Department of Molecular Microbiology and Bee Diseases, Friedrich-Engels-Str. 32, 16540 Hohen Neuendorf, Germany; 2 University of Potsdam, Institute of Biochemistry and Biology, Karl-Liebknecht-Str. 24-25, 14476 Potsdam-Golm, Germany; 3 Freie Universität Berlin, Department of Veterinary Medicine, Institute of Microbiology and Epizootics, Robert-von-Ostertag-Str. 7, 14163 Berlin, Germany

**Keywords:** Applied microbiology, Parasite biology

## Abstract

The Western honey bee *Apis mellifera*, which provides about 90% of commercial pollination, is under threat from diverse abiotic and biotic factors. The ectoparasitic mite *Varroa destructor* vectoring deformed wing virus (DWV) has been identified as the main biotic contributor to honey bee colony losses worldwide, while the role of the microsporidium *Nosema ceranae* is still controversially discussed. In an attempt to solve this controversy, we statistically analyzed a unique data set on honey bee colony health collected from a cohort of honey bee colonies over 15 years and comprising more than 3000 data sets on mite infestation levels, *Nosema* spp. infections, and winter losses. Multivariate statistical analysis confirms that *V. destructor* is the major cause of colony winter losses. Although *N. ceranae* infections are also statistically significantly correlated with colony losses, determination of the effect size reveals that *N. ceranae* infections are of no or low biological relevance.

## Abstract (web summary)

The analysis of a dataset collected over 15 years reveals no biological relevance of *Nosema ceranae* infections for colony losses; hence, *N. ceranae* is not considered a serious threat for honey bees in the background of *Varroa destructor* infestations.

## Introduction

The basis of human nutrition includes agriculturally grown crops and fruits, many of which are dependent on insect pollination for fruit set, seed production, and yield. Managed and wild insect pollinators, therefore, play a key role in ensuring that mankind is adequately supplied with food^1–3^. As a result, the health and survival of pollinating insects have attracted increasing public and scientific interest and consequently diseases as well as disease-causing pathogens and parasites that threaten pollinating insects have become the focus of many research studies. For pollinating insects, the main focus is on the Western honey bee *Apis mellifera* L., which is managed by beekeepers for honey production all over the world and provides 90% of the commercial pollination worldwide^2^. For pathogens and parasites, the focus is on those that threaten the survival of the managed honey bee colonies. The ectoparasitic mite *Varroa destructor* Anderson and Trueman together with deformed wing virus (DWV) vectored by the mite have been identified as the main contributors to colony losses^4–7^. The microsporidium *Nosema ceranae* Fries (*N. ceranae*) has also been implicated in regional colony losses^8–12^. The threat posed by these pathogens is compounded by the fact that honey bee colonies are usually infected by several pathogens simultaneously, with *V. destructor* (together with DWV) and *Nosema* spp. being the most widespread and therefore often occurring together^7,13–16^.

The mite *V. destructor* is an ectoparasite of honey bees (*Apis mellifera*, *A. cerana* F.) that infests honey bee colonies all over the world (for a recent review on *V. destructor* please see ref. ^17^). The life cycle of *V. destructor* in honey bee colonies is divided into two phases, (i) the dispersal phase in which adult female mites parasitize adult bees and use the bees as a means of transport and (ii) the reproductive phase that takes place in the capped brood cell^18^. For reproduction, a sexually mature, mated female mite enters a brood cell just before cell capping. When the honey bee larva reaches the prepupal stage, the mother mite begins laying eggs and raising her offspring. For feeding, the mother mite punctures a hole in the cuticle of the developing bee. This hole is then the feeding site for the growing mite family and allows access to the pupa’s fat body, which serves as nutritional resource^19^. Bees developing from *V. destructor* parasitized pupae show accelerated behavioral maturation, resulting in a shortened phase as nurse bees^20^, contribute less to colony productivity, and have a reduced longevity^18^. Heavily mite-infested colonies are characterized by an increasing rate of emerging bees which are not viable and have crippled wings^21^. Initially, these symptoms were thought to be caused solely by mite parasitization, but it soon became clear, that *V. destructor* is an efficient virus vector^22,23^ and that the crippled wings syndrome was caused by a virus, which was originally isolated as Egypt bee virus (EBV), but then renamed deformed wing virus (DWV)^24–27^. We now know that at least four major variants of DWV exist^27–30^ and that it is the variant DWV-B that causes most of the symptoms, is more virulent than the DWV-A, and uses *V. destructor* as biological vector^31–36^. Although *V. destructor* itself is sufficient to cause considerable damage to the parasitized pupa and the infested colony, it is the mite-vectored viruses, particularly deformed wing virus (DWV), that exacerbate the damage and link mite infestation to colony losses, especially during the winter season^5,7,29,34,37,38^.

Microsporidia are fungal-related, obligate intracellular parasites that infect many vertebrate and invertebrate host species^39^. Three microsporidian species infecting the adult Western honey bee *A. mellifera* are described: *Nosema apis*, *N. neumanni* n.sp., and *N. ceranae*. While *N. apis* is known as a honey bee-specific pathogen since more than 100 years^40^, *N. neumanni* was described as pathogen of *A. mellifera* in Uganda only recently^41^. *N. ceranae* in contrast was originally described as pathogen of the Eastern honey bee *Apis cerana*^42^, but obviously switched host several decades ago and by now is even more prevalent than *N. apis* in many *A. mellifera* populations^43–47^. However, a recent study has shown that there is still no general replacement of *N. apis* by *N. ceranae*, but replacement rather seems to be a regional phenomenon^48^, presumably influenced in its dynamics by climatic conditions since *N. ceranae* spores quickly lose their infectivity when exposed to low temperatures^49–51^.

From a clinical point of view, there is not much difference between *N. apis* and *N. ceranae*: Both pathogens follow a seasonal pattern and often cause asymptomatic infections of the midgut epithelium of adult bees^48^. Both pathogens can also cause diarrhea^52^, although the factors that cause the transition from asymptomatic to symptomatic infections are poorly understood^53,54^. Symptomatic outbreaks of *Nosema* spp.-infections are called nosemosis and can be diagnosed by the characteristic fecal spots visible at the hive entrance and inside the hive^52^. These fecal spots contain millions of infectious spores and drive the fecal-oral transmission of the disease within the colony, as adult bees cleaning the hive of the spots ingest the spores and become infected^26,55^. Infection in the individual adult bee host is initiated by germination of the ingested spores in the midgut lumen; germination is followed by extrusion of the polar tube, mechanical piercing of a cell by this polar tube, and injection of the sporoplasm into the cell through the polar tube^56,57^. The reproductive cycle within the infected host cell takes about 96 h^58^, goes through several stages (merogony, sporogony) and ends when the newly generated spores are released into the gut lumen by the bursting of the cell. These newly generated spores in the gut lumen are defecated and can infect naïve adult bees when they try to clean the hive from spore-contaminated fecal spots^26,55,59^.

In 2008, the first study was published suggesting that even asymptomatic *N. ceranae* infections result in the collapse of honey bee colonies^9^. Since then, numerous studies have been published demonstrating an association between *N. ceranae* infections and colony losses, but there are also many studies that failed to confirm this association^8–12,60–63^.

Among the studies that did not observe a statistically significant association between *N. ceranae* infection and colony losses is our longitudinal cohort study on *Nosema* spp. epidemiology and honey bee health in Northeast Germany^48,50^. In the course of a monitoring project, which was initiated in 2005 and is still ongoing, an extensive data set on colony health was continuously collected from a cohort of honey bee colonies, including data on colony losses during the winter season, prevalence of *Nosema* spp.-infection in autumn, and *V. destructor* infestation levels in autumn. We analyzed this unique 15-year data set comprising data on more than 3000 honey bee colonies using diverse uni- and multivariate statistical methods in order to investigate the relationship between colony mortality and the two pathogens that are most commonly blamed for colony losses, *V. destructor* and *N. ceranae*. We confirm that *V. destructor* is the main cause of colony winter losses, even in *N. ceranae*-infected colonies. However, in nearly mite-free colonies, a statistically significant association was demonstrated between *N. ceranae* infection in autumn and colony losses in the following winter. However, calculating the effect size of this correlation revealed that it has no biological relevance, since nowadays colonies not affected by *V. destructor* are rarely found. Thus, although *N. ceranae* is in principle capable of killing entire colonies, this effect is usually masked by the more dominant deleterious effect of the ubiquitous ectoparasite *V. destructor*. Our results end a long-standing controversy about the virulence of *N. ceranae* at the honey bee colony level and whether *N. ceranae* should be considered a serious threat to honey bee colonies.

## Results

### Winter mortality

Over the last 15 years, we performed a longitudinal cohort study on *Nosema* spp. epidemiology and colony health in the honey bee population in Northeast Germany^7,48,50^. We monitored between 180 and 270 colonies each year throughout the entire study period and continuously collected data on winter mortality as well as on *Nosema* spp. infection status and *Varroa destructor* infestation levels in the monitored colonies. The full data set for this study comprises 3502 colonies and is a uniquely solid basis for analyzing both, the dynamics of winter colony losses and the relation between *Nosema* spp.-infection and *V. destructor*-infestation in autumn with colony losses in the following winter.

**Fig. 1:**
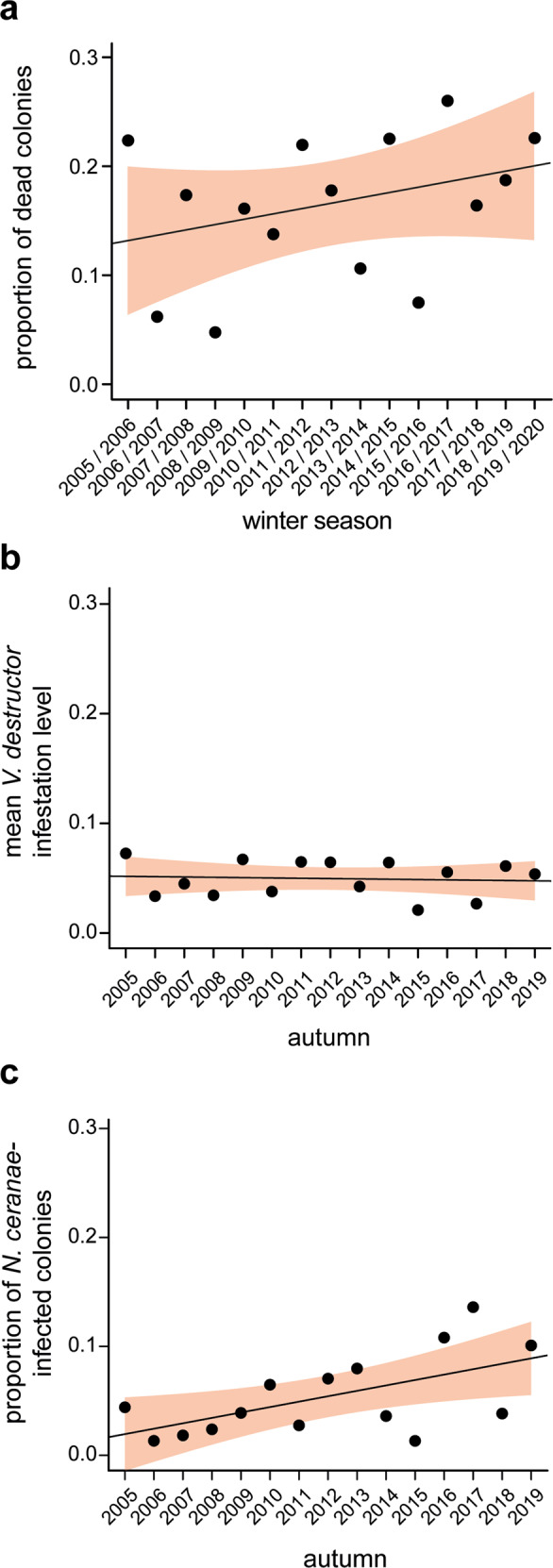
Dynamics of honey bee colony losses, *V. destructor* infestation rates, and *N. ceranae* infection prevalence within a cohort of monitored honey bee colonies. Honey bee colony losses over winter (**a**), *V. destructor* infestation levels in October (**b**), and *N. ceranae* infection prevalence in autumn (**c**) were monitored over the study period from 2005 to 2020. Each data point represents the proportion of dead colonies in spring (*n* = 3502), the mean mite load per 100 bees in October (*n* = 3492) or the prevalence of *N. ceranae*-infected colonies in autumn (*n* = 3502) per year. Linear regression models were calculated. Their regression lines are shown and their 95% CI (confidence interval) are highlighted in light red.

Within the study period, the winter colony loss rate varied from 4.8% as the lowest value in winter 2008/2009 to 26.0% as the highest value in winter 2016/2017. Using a linear model, we show that colony winter mortality increased by about 0.5 % per year in the studied cohort (Fig. 1a), but that this increase between 2005/2006 and 2019/2020 was not statistically significant (*p*-value of the *F*-statistic = 0.223, adjusted *R*^2^ = 0.043). The mean of the average winter losses was 16.31% ± 6.56% (mean ± SD) and therefore statistically significantly higher (*p*-value = 0.0023; one-sample *t*-test) than the empirical threshold for acceptable winter mortality of 10%^64^.

### Long-term dynamics of *Varroa destructor* infestations and *Nosema ceranae* infections

It is well proven and generally accepted that the mite *V. destructor* is the main cause of honey bee colony losses in winter and that mite infestation levels in autumn correlate with colony loss rates in the following winter [for a recent review see ref. ^17^]. We therefore monitored each colony for its mite infestation level in October over the entire study period 2005–2020 by counting the mites present in a bee sample of about 150 bees and expressing the infestation level as number of mites per 100 bees. The mean mite infestation rate varied between 7.26 ± 2.59 (mean ± SEM) in 2005 and 2.09 ± 1.00 (mean ± SEM) in 2015 and was unchanged throughout the study period according to the used linear model (*p*-value of the *F*-statistic = 0.7739, adjusted *R*^2^ = 0.006) (Fig. 1b). Hence, no statistically significant increase or decrease in mite infestation levels was observed.

We recently demonstrated that *N. ceranae* infection prevalence in autumn showed a statistically significant increase between 2005 and 2015^48^. We again analyzed the dynamics of *N. ceranae* infection prevalence in autumn over the entire study period of meanwhile 15 years using a linear model and demonstrate a steady increase in *N. ceranae* infection prevalence which was statistically significant (*p*-value of the *F*-statistic = 0.021, adjusted *R*^2^ = 0.295) and increased by about 0.5 % per year (Fig. 1c).

### *V. destructor* infestation and winter mortality

Although we observed a steady, though not significant, increase in winter losses in the cohort of monitored colonies over the 15-year observation period (Fig. 1a), there was no comparable trend in mean mite infestation rates (Fig. 1b). Nevertheless, a significant relationship between *V. destructor* parasitization and honey bee colony mortality could be established by comparing the mean mite infestation rates in colonies that died over the winter or survived the winter season. Calculated for the entire data set (*n* = 3492 colonies), the mean mite infestation rate in autumn was significantly higher (one-sample *t*-test, *p* < 0.0001) in colonies that collapsed during winter (13.67% ± 4.84%, mean ± SEM) than in colonies that were still alive in spring (3.27% ± 1.69%, mean ± SEM). Looking at the data for each year individually (Table 1) revealed that this significant overall relationship was true for 13 of the 15 years observed. Only in two winter seasons (2008/2009 and 2015/2016) with very low overall loss rates (4.8% and 7.5%, respectively) mite infestation level was not correlated with winter loss (Fig. 1a, Table 1). There is a simple explanation for this phenomenon: if winter losses are below a biological threshold (natural colony mortality of about 10%^64^), chances that *V. destructor* killed the colonies are low.

**Table 1:** Data on *V. destructor* infestation levels in autumn in colonies that survived or collapsed during the winter seasons over the entire study period from autumn 2005 until spring 2020.

Winter season 2005 to 2020	Colony losses in winter [%]	Total no. of colonies analyzed in autumn	Mean percentage of mites per year^a^			*p*-value^c^
			All colonies^b^	Survived colonies^b^	Collapsed colonies^b^	
05/06	18.9	227	7.26 ± 12.43	4.7 ± 9.2	18.4 ± 17.6	<0.001
06/07	6.2	226	3.36 ± 8.63	2.6 ± 7.0	14.2 ± 19.2	<0.05
07/08	17.4	219	4.49 ± 7.85	2.6 ± 4.5	13.3 ± 12.9	<0.001
08/09	4.8	210	3.44 ± 9.63	3.2 ± 9.8	7.0 ± 6.1	=0.09
09/10	16.1	180	6.71 ± 12.24	3.8 ± 7.0	21.6 ± 21.3	<0.001
10/11	13.8	247	3.78 ± 7.10	2.4 ± 4.5	12.9 ± 12.5	<0.001
11/12	22.0	255	6.48 ± 11.11	4.1 ± 6.6	14.7 ± 17.7	<0.001
12/13	17.8	270	6.45 ± 9.42	5.0 ± 7.3	13.0 ± 14.4	< 0.001
13/14	10.6	226	4.24 ± 8.33	3.6 ± 7.3	9.4 ± 13.4	<0.05
14/15	22.5	222	6.43 ± 15.41	3.3 ± 5.7	17.2 ± 28.4	<0.01
15/16	7.5	227	2.09 ± 4.82	2.1 ± 4.9	2.3 ± 3.7	=0.86
16/17	26.0	250	5.54 ± 10.23	2.8 ± 4.4	13.3 ± 16.7	<0.001
17/18	16.4	250	2.67 ± 4.54	2.0 ± 2.9	6.6 ± 8.5	<0.01
18/19	18.7	235	6.10 ± 14.63	3.2 ± 6.6	18.9 ± 27.7	<0.001
19/20	22.6	248	5.37 ± 11.46	3.9 ± 6.3	10.4 ± 20.4	<0.05

^a^*V. destructor* mites per 100 bees.

^b^Mean ± SD.

^c^For survived vs. collapsed colonies, determined by the one-sample *t*-test.

### Classification tree analysis

Our results reconfirmed what is already known from many other studies, namely that *V. destructor* is the main driver of honey bee winter mortality. However, *N. ceranae* has also been implicated in colony losses in many studies, although we have not yet been able to confirm this in our previous analyses of our cohort study data: A possible explanation is that these previous analyses were based on univariate analyses that observe the effect of single explanatory variables on colony mortality but are unable to estimate the relative influence of *V. destructor* and *N. ceranae* on colony mortality when both are present simultaneously.

**Fig. 2:**
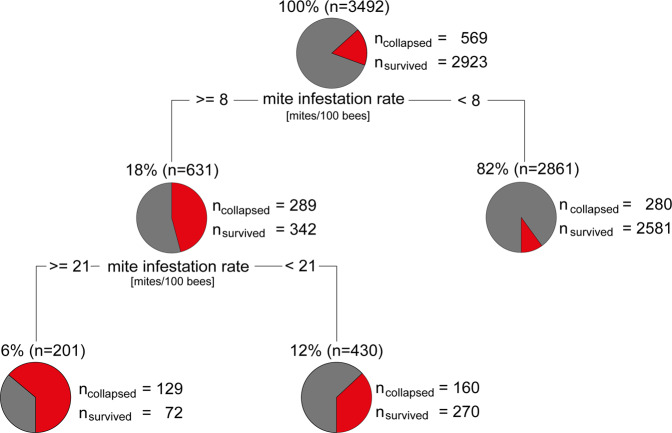
Testing the relevance of the attributes “mite infestation rate” and “*Nosema* infection status” for honey bee colony mortality by classification tree analysis. The proportion of colonies represented by each circle (leaf/outcome) is shown above the respective circle. In each circle, the gray parts represent the proportions of overwintered (survived) colonies and the red parts the proportions of collapsed colonies. The number of the collapsed and survived colonies are given next to each circle. The tested attribute that resulted in each of the two nodes is given below the circles (mite infestation rate). Horizontal lines represent the branches/decisions and the numbers within these lines show the mite infestations rates that best splitted the data to obtain the next nodes or leafs. For classification tree analysis, the R packages rpart^81^ and rattle^82^ with standard settings were used.

To close this gap of knowledge, we used our data set comprising for each colony qualitative (yes/no) data on colony winter losses, quantitative data on *V. destructor* infestation levels and both qualitative (infecting species) and semi-quantitative (categorized spore load) data on *Nosema* spp.-infection status and performed a classification tree analysis (Fig. 2), a tool of recursive partitioning for multivariate data exploration. This multivariate analysis aimed at identifying the relative impact of *V. destructor* infestation levels and *Nosema* spp. infection status (infecting species, spore load category) in autumn on the fate of the colony over winter, hence, whether a colony was prone to collapse over winter or had a realistic chance to survive.

A total of 3492 data sets was available for this analysis. Mite infestation rate was identified as the attribute that best classified our data set and hence became the root node (Fig. 2). Performing a top-down search, two decision points (nodes) describing the fate of the colony (survival or collapse) were identified (Fig. 2). Both were solely based on the attribute “mite infestation level” indicating that the also tested attributes regarding the *Nosema* spp.-infection status of the colonies (infecting species and spore load category) were not identified by this analysis as decisive factor for colony collapse over winter. The first decision point divided the analyzed colonies into two groups, one comprising 82% (*n* = 2861) of the colonies with a mite infestation rate in October of less than eight mites per 100 bees and a mortality rate of 9.8%. The remaining 18% of the colonies (*n* = 631) had an infestation level of eight or more mites per 100 bees and a mortality rate of 45.8%. This group was further subdivided into two groups, again based on the mite infestation level. 12% (*n* = 430) had an infestation level of eight or more mites but below 21 mites per 100 bees and a mortality rate of 37.2%, whereas the other branch comprised the remaining 6% (*n* = 201) of the colonies which were characterized by an infestation level of 21 or more mites per 100 bees and a mortality rate of 64.2%. This classification tree showed convincingly that over the study period of 15 years, the strongest link was between colony winter losses and *V. destructor* infestation level, but not with *N. ceranae* or *N. apis* infection.

### *N. ceranae* infection and winter mortality

**Fig. 3:**
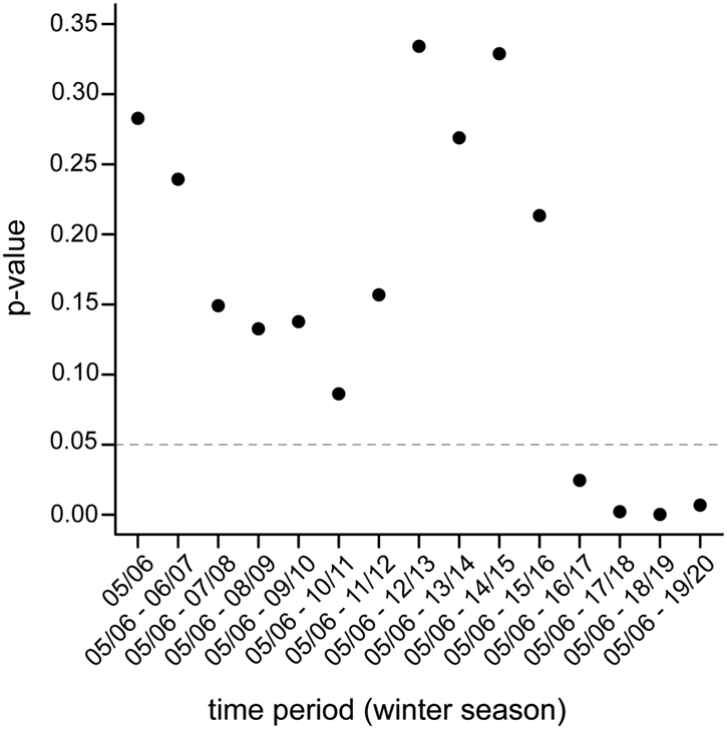
Calculated p-values for the relation between colony losses and *Nosema* spp.-infection status using cumulated data for the numbers of infected colonies. Each data point represents the cumulative number of infected colonies summed over the time window indicated on the *x*-axis. The total numbers per time window are given in Table 2. The *y*-axis gives the respective *p*-value of the *χ*^2^ test.

The results from the classification tree analysis were in accordance with previous studies that did also not reveal any relation between colony winter losses and *Nosema* spp. infection^7,48,50,63,65,66^, but contradicted other studies repeatedly showing that *N. ceranae* infections cause colony losses^9,10,12,67,68^. A striking feature of our data set is that both winter losses (Fig. 1a) and N*. ceranae* prevalence (Fig. 1c) each increased by 0.5 % per year over the observation period of 15 years. This coincidence led us to suspect that there was, after all, a link between winter losses and *N. ceranae* infections that we had previously overlooked^7,48,50^. Fact is that *Nosema* spp.-infections usually show a rather low prevalence in autumn^26,48^, and hence our data set for each autumn comprises only a few colonies infected with *Nosema* spp. and even fewer colonies infected with *N. ceranae* or representing a particular spore load category. We, therefore, speculated that the number of *Nosema* spp. infected colonies per year might be too low to see a relation between winter mortality and *Nosema* spp. infection. Hence, we decided to generate higher numbers for *Nosema* infected colonies by ignoring both the species differentiation and the infection categories and summing the numbers for the individual years starting with autumn/winter 2005/2006 and ending with the sum of autumn/winter 2005/2006 up to autumn/winter 2019/2020 (Table 2). Forming these cumulative data subsets and examining time periods instead of annual values, resulted in larger group sizes (n’s) and overall larger numbers of *Nosema* spp. infections, which made statistical analyses more robust (Table 2). We used a Chi-squared test to calculate the statistical relationship between infection status and winter mortality and indeed, the analysis started to become significant (p-value < 0.05) when more than 11 years (2005/2006 up to 2016/2017 resulting in more than 221 *Nosema* spp.-infected colonies among the analyzed 3492 colonies) were considered (Table 2, Fig. 3).

**Table 2:** Cumulative data subsets for determining the effects of Nosema spp.-infection in the autumn on honey bee colony losses in the following winter.

Cumulative overwintering period	Total number of colonies	Surviving colonies		Collapsed colonies		*p*^a^
		*Nosema* positive	*Nosema* negative	*Nosema* positive	*Nosema* negative	
2005	237	21	163	9	44	0.28
2005–2006	463	40	356	10	57	0.24
2005–2007	682	51	526	14	91	0.15
2005–2008	892	62	715	14	101	0.13
2005–2009	1072	75	853	17	127	0.14
2005–2010	1319	96	1045	22	156	0.09
2005–2011	1574	111	1229	26	208	0.16
2005–2012	1844	133	1429	29	253	0.33
2005–2013	2070	166	1598	35	271	0.27
2005–2014	2292	175	1761	38	318	0.33
2005–2015	2519	182	1964	39	334	0.21
2005–2016	2769	203	2128	53	385	0.02
2005–2017	3019	234	2306	66	413	<0.01
2005–2018	3254	240	2491	73	450	<0.001
2005–2019	3502	267	2656	74	505	<0.01

^a^Determined by the *χ*^2^ test.

**Fig. 4:**
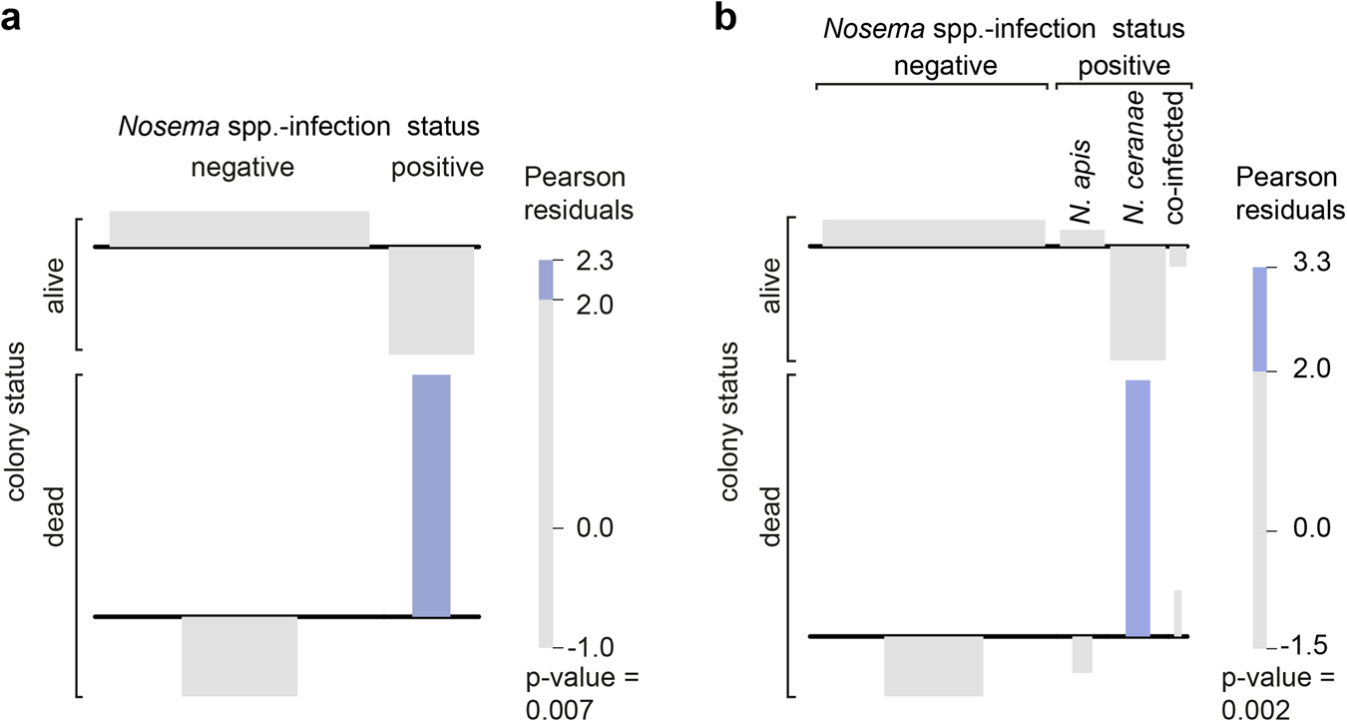
Contingency table analysis. Association plots based on Chi-squared testing show the association between **a** *Nosema* spp.-infection and colony mortality and **b** *N. apis*-, *N. ceranae* and co-infections and colony mortality. Overrepresented entries (dead/positive in **a** or dead/*N. ceranae* in **b**) are shown in blue, non-overrepresented entries in gray.

To further support and analyze this impact, we performed a Chi-squared test giving Pearson residuals using the data cumulated over the entire study period (2005/2006 to 2019/2020) for *Nosema* spp. infection (Fig. 4a) and for N. *apis*, *N. ceranae* and mixed infections separately (Fig. 4b). The black lines in the associations plots (Fig. 4) represent the expected values for the categories “alive” or “dead” in relation to the infection categories *Nosema* spp.-negative or -positive (Fig. 4a) or negative and positive for *N. apis*-, *N. ceranae*- or co-infections (Fig. 4b). Overrepresented categories are represented by rectangles above the base line, while the rectangles for underrepresented categories are below the base line. Categories with Pearson residuals above 2.0 are shown in blue and appear only for the combinations “dead” and “positive for *Nosema* spp.-infection” (Fig. 4a) or “dead” and “positive for *N. ceranae* infection” (Fig. 4b). These results confirmed that the relation between *Nosema* spp. infection and winter colony losses was statistically significant (*p*-value = 0.007). However, this was only true for *N. ceranae*-infections (*p*-value = 0.002); *N. apis*- or co-infections did not significantly contribute to winter losses suggesting that *N. ceranae* is indeed more virulent at colony level than *N. apis*.

### Statistical significance vs. biological relevance

**Fig. 5:**
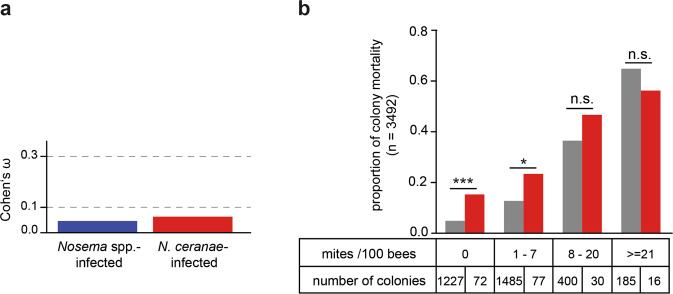
Biological relevance of *N. ceranae* infections. **a** Bar plot showing the effect size Cohen’s ω for *Nosema* spp.- (blue bar) and *N. ceranae*-infected (red bar) colonies on colony mortality. Dashed lines indicate the conventional definition of Cohen’s ω with a value for ω between 0.1 and 0.3 as small, between 0.3 and 0.5 as medium and above 0.5 as large effect^69^. **b** Bar plot showing the relation between colony mortality, mite infestation level categories (mites per 100 bees) and *N. ceranae*-infection status (gray bars = not infected with *N. ceranae*, red bars = infected with *N. ceranae*). Significance levels, which resulted from *p*-values of the *χ*^2^-tests, are indicated by asterisks (n.s., *p* ≥ 0.05; significantly different: *0.05 < *p* < 0.01; **0.01 < *p* < 0.001; ***0.001 > *p*).

P-values are a measure of statistical significance, but are insufficient to show biological relevance. To analyze the biological relevance of our results, we therefore used the effect size measure Cohen’s ω, which is applicable for two times two and larger contingency tables and is considered a measure of relevance with values above 0.1, 0.3, and 0.5 indicating a small, medium, and large effect size, respectively^69^. Calculating Cohen’s ω for our data set and the relation between colony losses and *Nosema* spp.- or *N. ceranae*-infection revealed an effect size below 0.1, hence, a less than small effect (Fig. 5a). These results indicated that although we showed a statistically significant relationship between colony losses and *Nosema* spp. infection, particularly *N. ceranae* infection, these relationships are of minor or no biological relevance supporting the results of the classification tree analysis (Fig. 2) identifying *V. destructor* as main factor in colony losses.

We therefore reanalyzed the data, but this time we considered the *V. destructor* infestation rate of the colonies by analyzing the mortality rate of colonies infected and not infected with *N. ceranae* (Fig. 5b) within the mite infestation categories as defined by the classification tree (Fig. 2). This analysis revealed that *N. ceranae* infection only contributed significantly to colony mortality when the colonies did not harbor detectable *V. destructor* mites or very few mites (1-7 per 100 bees) in October. Only in these 149 out of 3474 colonies, *N. ceranae* was significantly correlated with winter mortality (Fig. 5b).

## Discussion

Honey bee colony losses and the quest for abiotic and biotic factors causing them are hot topics in the field of bee research since two decades. While it is widely accepted and unequivocally substantiated by many studies, that the ectoparasitic mite *V. destructor* and the viruses vectored by the mite play a key role in winter colony losses, the role of *N. ceranae* infections is less clear. There are studies clearly showing detrimental effects of *N. ceranae* infection on honey bee colonies^8–12^. But there are also numerous monitoring studies that fail to observe such an association between *N. ceranae* infection and colony losses^48,50,60–62^. One possible reason for this could be that in monitoring studies the damage caused by the almost ubiquitous infestation of colonies with *V. destructor* masks the effects caused by pathogens with rather low prevalence such as *N. ceranae*. This masking effect is difficult to see through because most observational studies on colony losses include too few colonies and are conducted over too short a time period to observe statistically significant associations for low-prevalence pathogens. Moreover, although most monitoring studies collect data for multiple pathogens, more complex or multivariate analyses on the interaction between pathogens or their joint effect and relative influence on colony mortality are rarely performed. There is one study analyzing the interaction of pathogens and showing, that *N. ceranae*-infections in spring correlate statistically significantly with an increased prevalence of *Ascosphaera apis* infections and higher levels of *V. destructor* infestation in summer^70^. However, data on the relative impact of individual co-infecting pathogens on colony mortality in the field are lacking, although it is widely accepted that colony collapse is a multifactorial process, often likely involving more than one pathogen^13^.

Our data on *V. destructor* and *Nosema* spp. load and winter colony mortality, collected continuously over 15 years from a relatively stable cohort of about 25 apiaries contributing ten colonies each is a unique resource to study the role of *V. destructor* and *N. ceranae* on colony mortality in the field, especially the relative impact of these two parasites on overwintering success of honey bee colonies, most of which were concurrently infested by *V. destructor* and infected with *Nosema* spp.

With more than 3000 data sets collected over 15 years, we were able to confirm that big data sets and long study durations are key for robust analyses: Only by summing the data for more than 11 years did a statistically significant association between *N. ceranae-*, but not *N. apis*-infection in the autumn and colony losses the following winter become evident. This result indicated that at colony level *N. ceranae* is indeed more virulent than *N. apis* confirming previous studies on *N. ceranae*-induced colony losses^8–12^. No such relation between *N. ceranae*-infections and colony losses was observed when the data were analyzed year by year^48,50^ because the prevalence of *Nosema* spp.-infections in autumn and the mortality rate among these colonies are usually too low for statistical significance. This in turn explains previous studies that did not support *N. ceranae*-induced colony losses^48,50,60–62^.

A statistically significant association between *N. ceranae* infection and colony losses was in accordance with studies that had suggested an increased colony virulence of *N. ceranae* compared to *N. apis* and the abilty of *N. ceranae* to cause the collapse of entire colonies^8–12^. However, our multivariate data exploration via classification tree analysis had identified *V. destructor* infestation as the main variable explaining colony losses in our cohort. A result that is also in accordance with many other studies clearly linking mite infestation, but not *N. ceranae* infection, to winter colony mortality^5,7,29,34,37,38^. Only with a mite infestation rate in October of less than eight mites per 100 bees, an acceptable and natural winter mortality rate below 10%^64^ can be reached. In the monitored cohort this was the case for 82% of the colonies over the entire duration of the study, leaving 18% of the cohort contributing to unacceptably elevated colony losses. Since classification tree analysis is the method of choice for determining biologically relevant factors and the classification tree identified mite infestation as the only relevant factor, it was not surprising that the determination of Cohen’s ω confirmed that the biological relevance of *N. ceranae* infection for colony losses is low despite the statistical significance of this association. This clearly shows that for biological questions the focus should rather not solely lay on statistical significance but more on the effect size and biological relevance.

Hence, our results confirmed that *V. destructor* is the major cause of colony winter losses, although *N. ceranae* infections can also have deleterious effects. However, these effects are normally masked by the more severe effects of *V. destructor* on colony health and therefore only detectable in colonies that are not infested with mites or are infested at low levels. As long as *V. destructor* infestation is the dominating health problem in honey bee colonies and *N. ceranae* prevalence is low, *N. ceranae* can be classified as pathogen causing little concern, because its role in colony losses is marginal. Therefore, we do not yet consider *N. ceranae* a serious threat to honey bee colonies. However, the situation might change when the prevalence of *N. ceranae* reaches a critical point. Since the increase in *N. ceranae* prevalence is continuing (ref. ^48^ and this study), it might only be a question of time when this point will be reached. Monitoring not only mite infestation levels in colonies but also *N. ceranae* infection prevalence in honey bee populations is therefore advisable. Hence, we will continue our study and continuously calculate the effect size of *N. ceranae* infection on colony losses to determine the critical prevalence of *N. ceranae* in a honey bee population.

Remarkable is that in an early report on colony collapse due to *N. ceranae*^10^ it was explicitly pointed out that mites were absent in all samples indicating a very low number or even the total absence of *V. destructor* in these collapsed colonies due to efficient mite control. The absence of *V. destructor* and concomitant presence of *N. ceranae* was the most convincing argument for *N. ceranae* being the cause of colony collapse in the reported case. Moreover, for experimentally demonstrating *N. ceranae*-induced colony collapse, the colonies needed to be tightly controlled for *V. destructor* infestation^9^. These studies indirectly corroborate our results which indicated that *N. ceranae*-induced colony collapse only becomes evident in (nearly) mite-free colonies. Hence, one can say that the more efficient the mite control is, the more likely it is that *N. ceranae* induced damages become detectable. But again, as long as the prevalence of *N. ceranae* is low, this does not pose a serious threat because *N. ceranae* is simply not a highly virulent pathogen. With these results we end a long-standing controversy about whether *N. ceranae* is capable of killing entire honey bee colonies—yes, it is under certain circumstances—and should be considered a serious threat for honey bees in general—no, rather not as long as *V. destructor* infestations are the dominating health problem.

## Methods

### Bee samples, field survey

**Fig. 6:**
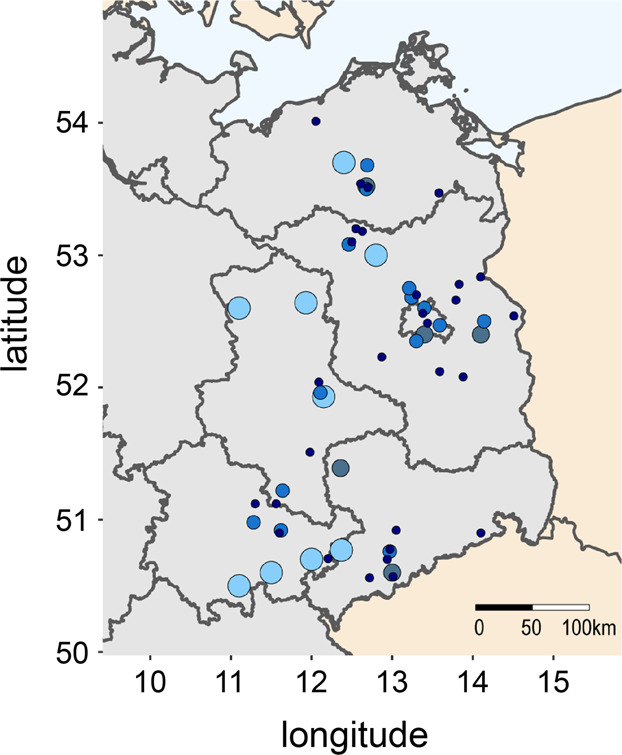
Map section of Northeast Germany showing the location of the apiaries which participated in the study. Northeast Germany is shown in gray with differently sized circles in different shades of blue showing the locations of the apiaries. The size and color of the circles represent the number of years for which data are available for each apiary (light blue, 12–15 years; gray-blue, 9–11 years; royal blue, 5–8 years; dark blue, 1–4 years). The map of Northeast Germany was sourced from the Database of Global Administrative Areas (http://GADM.org)^86^.

The data set of this study comprises data and samples which were collected from autumn 2005 to spring 2020 in the course of a 15 year longitudinal cohort-study on *Nosema* spp. epidemiology and honey bee health (Supplementary Data 1)^7,48,50^. Honey bee samples were collected in autumn and colonies were checked for their survival in spring of the respective overwintering period (week 36 to week 14 of the following year) from about 23 apiaries which were located in Northeast Germany (Fig. 6). Briefly, apiaries participated with ten so called “monitoring colonies” each. Apiaries or monitoring colonies that dropped out during the study period were substituted by adequate replacement. Hence, more than half of the apiaries (14 of ~23) participated for more than 9 years and 5 of them even for the entire duration of the study, i.e., 15 years. From at least 19 apiaries, samples were provided over a time period of consecutive 5–11 years (Fig. 6). This resulted in an annual mean of 23.4 ± 2.26 (mean ± SD) apiaries with 9.77 ± 1.25 (mean ± SD) colonies each, giving an overall count of *n* = 3502 sampled monitoring colonies which provide the basis of our analyses.

Sampling of bees for subsequent pathogen analysis was performed between calendar week 36 and 38 (late September/beginning of October)^48,50^. About 300 in-hive honey bees were sampled from a super above the queen excluder^71^ from each monitoring colony. Bee samples were stored at −20 °C until further analysis. At least 150 bees were used for determining *V. destructor* infestation levels, 20 bees were used for determining the *Nosema* spp. infection status and the remaining bees were stored as retention sample in case analyses had to be repeated.

### Determination of mite infestation levels

For determining the mite infestation level of a colony, *V. destructor* mites were washed from about 150 sampled bees following a standard protocol using a detergent solution^72^. Briefly, the frozen bees were covered with soap and water in a jar. Subsequently, the jar was shaken for 20 s and emptied into two stacked sieves with a white nylon cloth between them. All mites were rinsed with plenty of water under high pressure through the upper sieve which has larger apertures (3–4 mm) not allowing bees to pass. All mites were collected on the cloth in the second sieve, which has smaller apertures (<0.5 mm) that no mite fits through. To gain the mite infestation level of an individual colony in %, the number of counted mites was divided by the number of sampled and washed bees multiplied by 100. The mite infestation levels for each winter season (mean percentage of mites per year; Table 1) for the three categories “all colonies”, “survived colonies”, and “collapsed colonies” were calculated on the basis of the individual colony values by calculating the mean (±SD) over all colonies of the respective category in the respective winter season. The mean (±SEM) mite infestation levels for the entire study period for each of the three categories were calculated on the basis of these annual means (Table 1). Unfortunately, mite infestation rate could not be determined in one apiary (ten colonies) in the first year (2005/2006) because of an insufficient number of honey bees available. Therefore, the dataset for mite infestation level had to be reduced from 3502 to 3492 (Table 1).

### Diagnosis of *Nosema* spp. infection and molecular species differentiation

Diagnosis of *Nosema* spp. infections was performed in accordance with the “Manual of Standards for Diagnostics and Vaccines” published by the Office International des Epizooties (OIE), the World Organization for Animal Health^7,48,50,73^. In short, per colony 20 pooled bee abdomens were homogenized in 4 ml tap water (200 µl/bee) and microscopically examined for the presence of spores. The moderate sample size of 20 bees also given in the OIE manual is considered sufficient when the study unit is the honey bee colony^74,75^. This sample size allows to microscopically determine detectable levels of infection above 15% with 96% probability of detection^76,77^. This level of infection in a colony was considered biologically relevant^9^. Infection levels were determined by counting the number of *Nosema* spp. spores per view field (three technical replicates each) in a hemocytometer (Neubauer-improved, VWR, Darmstadt, Germany) using an inverse microscope (VWR, Darmstadt, Germany) with ×100 magnification. Since the view field represents 0.1 µl, the detection threshold is 1 spore in 0.1 µl, hence, 40,000 spores per pooled sample or 2000 spores per bee on average. For classification of the infection levels, standard categories were used^73^: 0 (no spores), 1 (1–10 spores), 2 (11–100 spores), and 3 (more than 100 spores).

*Nosema* spp.-positive samples were subjected to further molecular species differentiation either via PCR-amplification of a conserved region of the 16S rRNA gene followed by RFLP (restriction fragment length polymorphism) analysis of this amplicon^43,50^ or via a species-specific duplex PCR-protocol taking advantage of species-specific sequence differences in the highly conserved gene coding for the DNA-dependent RNA polymerase II largest subunit^78^. Molecular differentiation enabled the distinction between single infections with either *N. apis* or *N. ceranae*, or co-infections where both infections are present at the same time (Table 3).

**Table 3:** Data on *Nosema* spp. epidemiology (prevalence of *Nosema* spp.-, *N. apis*-, *N. ceranae*- and co-infections) and winter losses collected between autumn 2005 and spring 2020.

Winter season	Total no. of colonies analyzed in autumn	No. of colonies between weeks 36 and 14						Colony losses [%]
		Colonies alive in spring (survivors)			Colonies dead in spring (winter loss)			
		Total	*Nosema* positive^a^	*Nosema* negative	Total	*Nosema* positive^a^	*Nosema* negative	
2005/2006	237	184	21 (16, 4, 1)	163	53	9 (3, 6, 0)	44	22.4
2006/2007	226	212	19 (14, 3, 2)	193	14	1 (1, 0, 0)	13	6.2
2007/2008	219	181	11 (7, 3, 1)	170	38	4 (3, 1, 0)	34	17.4
2008/2009	210	200	11 (6, 5, 0)	189	10	0 (0, 0, 0)	10	4.8
2009/2010	180	151	13 (7, 6, 0)	138	29	3 (2, 1, 0)	26	16.1
2010/2011	247	213	21 (9,12, 0)	192	34	5 (1, 4, 0)	29	13.8
2011/2012	255	199	15 (6, 5, 4)	184	56	4 (1, 2, 1)	52	22.0
2012/2013	270	222	22 (4, 18, 0)	200	48	3 (2, 1, 0)	45	17.8
2013/2014	226	202	33 (16, 13, 4)	169	24	6 (1, 5, 0)	18	10.6
2014/2015	222	172	9 (4, 5, 0)	163	50	3 (0, 3, 0)	47	22.5
2015/2016	227	210	7 (5, 2, 0)	203	17	1 (0, 1, 0)	16	7.5
2016/2017	250	185	21 (6, 15, 0)	164	65	14 (2, 12, 0)	51	26.0
2017/2018	250	209	31 (6, 24, 1)	178	41	13 (1, 10, 2)	28	16.4
2018/2019	235	191	6 (1, 5, 0)	185	44	7 (2, 4, 1)	37	18.7
2019/2020	248	192	27 (2, 24, 1)	165	56	1 (0, 1, 0)	55	22.6

^a^The numbers of colonies positive for *N. apis*, for *N. ceranae*, and for both (mixed infections) are given in this order in parentheses.

### Statistics

Data were curated, transformed, and presented in spreadsheets for the analysis with the statistic software R^79^ using the package *openxlsx*^80^ (Tables 1, 2, and 3). The statistical analysis of mean percentage of *Varroa* infestation in survived colonies and collapsed colonies was determined by the one-sample *t*-test for the entire observation period as well as for each winter season individually (Table 1).

For the dynamics of winter losses, mean mite infestation levels, and *N. ceranae* infection prevalence, we performed linear regression models (using R base package *stats*) and calculated its regression line, its slope, the adjusted *R*^2^ and the *F*-statistic (Fig. 1a–c).

To describe and visualize which variable(s) has (have) the largest share in honey bee colony mortality, we took advantage of classification tree analysis (decision trees), a tool of recursive partitioning for multivariate data exploration. We constructed a tree (Fig. 2) with default settings using *rpart*^81^ and *rattle*^82^. In the construction of the tree, the binary target variable was colony mortality over winter (yes/no) and the tested attributes on each colony were the mite infestation levels and the *Nosema* infection status, i.e. the infecting *Nosema* species (*n* = 3151 non-infected, *n* = 128 for *N. apis*, *n* = 195 for *N. ceranae*, and *n* = 18 for mixed infections) and the respective spore load categories (*n* = 3151 for infection category 0, *n* = 140 for category 1, *n* = 141 for category 2, and *n* = 60 to category 3). In the obtained tree, each node represents an attribute, each branch represents a decision, and each leaf represents the outcome of the decision.

To understand the role of *N. ceranae* infection in more detail, we looked at the *Nosema* spp. infection frequencies using contingency tables (Table 2) and performed chi-squared calculation (*χ*^2^-calculation). The results were plotted in a dot plot (Fig. 3) and two-way association plots (Fig. 4) created by *vcd*^83–85^. The obtained results of the significance statistic were further assessed by strength statistic using the effect size index Cohen’s ω^69^, which is an index for the biological effect size between two categorical variables (Fig. 5a). Cohen’s ω is calculated as follows:

1 $$\omega =\root{2}\of{{\sum }_{i=1}^{m}\frac{{({p}_{1i}-{p}_{0i})}^{2}}{{p}_{0i}}}$$

*p*_1i_ = the proportion in cell i posited by the alternate hypothesis and reflects the effect for that cell; *p*_0i_ = the proportion in cell i posited by the null hypothesis; *m* = number of cells [^69^ p. 216 formula 7.2.1]. For the interpretation of Cohen’s ω in terms of effect size, there is a framework of conventional definition saying that 0.1 ≤ ω < 0.3 is a small, 0.3 ≤ ω < 0.5 is a medium and ω ≥ 0.5 is a large effect size [^69^ chapter 7.3 p. 227]. To obtain the relation of colony mortalities depending on the two factors *Nosema*-infection and *Varroa*-infestation, chi-squared tests were performed and visualized by a bar plot (Fig. 5b).

The map section of Northeast Germany (Fig. 6) showing the boundaries of the Northeastern Federal States of Germany was sourced from the Database of Global Administrative Areas (http://GADM.org)^86^. Data on the location of the apiaries and the duration of participation were inserted by the use of the following R packages: *rnaturalearth*^87^, *raster*^88^, *ggplot2*^89^, *sf*^90^, *sp*^91,92^, *rgeos*^93^, and *reshape*^94^.

### Reporting summary

Further information on research design is available in the Nature Portfolio Reporting Summary linked to this article.

## Supplementary information

Supplementary Data 1

Reporting Summary

## Data Availability

All data generated or analyzed during this study are included in this published article (and its supplementary information files). The numerical data for the analyses and graphs are provided in Supplementary Data 1.
