# Supplementary material for: Significant, but not biologically relevant: Nosema ceranae infections and winter losses of honey bee colonies
Source: Commun Biol. 2023 Mar 1;6:229. doi: 10.1038/s42003-023-04587-7 (PMC9977864; doi:10.1038/s42003-023-04587-7)
Supplement: Supplementary file 1 — Supplementary Data 1 [file 42003_2023_4587_MOESM1_ESM.pdf]

Supplementary Information

Significant, but not biologically relevant: *Nosema ceranae* infection and winter losses of honey bee colonies.

Vivian Schüler[1], Yuk-Chien Liu[1,2], Sebastian Gisder[1], Lennart Horchler[1], Detlef Groth[2], Elke Genersch[1,3] *

[1] Institute for Bee Research, Department of Molecular Microbiology and Bee Diseases, Friedrich-Engels-Str. 32, 16540 Hohen Neuendorf, Germany
[2] University of Potsdam, Institute of Biochemistry and Biology, Karl-Liebknecht-Str. 24-25, 14476 Potsdam-Golm, Germany
[3] Freie Universität Berlin, Department of Veterinary Medicine, Institute of Microbiology and Epizootics, Robert-von-Ostertag-Str. 7, 14163 Berlin, Germany

* Corresponding author

Table of Contents

Supplementary Table 1

Supplementary Table

Supplementary Table 1. Data collected from autumn 2005 to spring 2020 on winter colony losses (determined in spring) as well as *Nosema* spp. infection status and mite infestation load (both determined in autumn).

year	Name of apiary	Winter colony loss - in spring	*Nosema* spp. infection status – in autumn				Mite infestation load in autumn: *V. destructor*/ 100 bees
			Nosema infection category	*Nosema .apis*	*Nosema .ceranae*	*Nosema* .mix	
2005	10	1	0	0	0	0	no data
2005	10	1	0	0	0	0	no data
2005	10	1	0	0	0	0	no data
2005	10	1	0	0	0	0	no data
2005	10	1	0	0	0	0	no data
2005	10	1	0	0	0	0	no data
2005	10	1	0	0	0	0	no data
2005	10	1	0	0	0	0	no data
2005	10	1	0	0	0	0	no data
2005	10	1	0	0	0	0	no data
2005	13	0	0	0	0	0	13,3
2005	13	0	0	0	0	0	37,9
2005	13	0	0	0	0	0	25
2005	13	0	0	0	0	0	34,8
2005	13	0	0	0	0	0	70,4
2005	13	0	0	0	0	0	5,7
2005	13	0	0	0	0	0	32,4
2005	13	0	0	0	0	0	26,9
2005	13	0	1	1	0	0	6,7
2005	13	0	0	0	0	0	14,3
2005	36	1	2	0	1	0	9,4
2005	36	1	2	0	1	0	6,1
2005	36	1	2	0	1	0	14,3
2005	36	1	0	0	0	0	19,4
2005	36	1	0	0	0	0	20,7
2005	36	1	2	0	1	0	4,1
2005	36	1	3	0	1	0	22,7
2005	36	1	0	0	0	0	31,3
2005	36	1	0	0	0	0	20,7
2005	36	1	2	0	1	0	10,5
2005	1	0	0	0	0	0	7,4
2005	1	0	0	0	0	0	0
2005	1	0	0	0	0	0	19,4
2005	1	0	0	0	0	0	5,9
2005	1	0	0	0	0	0	0
2005	1	0	0	0	0	0	0
2005	1	0	0	0	0	0	2,8
2005	1	0	0	0	0	0	23,3
2005	1	0	0	0	0	0	7,7
2005	1	0	0	0	0	0	2,9
2005	41	1	0	0	0	0	22,6
2005	41	0	0	0	0	0	11,1

2005	41	1	0	0	0	0	71
2005	41	1	0	0	0	0	7
2005	41	1	0	0	0	0	24,1
2005	41	0	0	0	0	0	12,5
2005	41	0	0	0	0	0	3,6
2005	41	1	0	0	0	0	20,7
2005	41	1	1	1	0	0	23,5
2005	41	1	0	0	0	0	14,8
2005	23	1	0	0	0	0	9,4
2005	23	1	0	0	0	0	80
2005	23	1	0	0	0	0	45,9
2005	23	1	0	0	0	0	38,5
2005	23	0	3	0	0	1	0
2005	23	1	0	0	0	0	43,8
2005	23	1	0	0	0	0	0
2005	23	1	0	0	0	0	20,7
2005	23	0	0	0	0	0	12,5
2005	23	1	0	0	0	0	24,1
2005	6	1	0	0	0	0	26,9
2005	6	0	0	0	0	0	26,9
2005	6	0	0	0	0	0	3,8
2005	6	0	0	0	0	0	0
2005	6	0	2	1	0	0	6,24
2005	6	0	0	0	0	0	0
2005	6	0	0	0	0	0	0
2005	6	1	0	0	0	0	9,5
2005	6	0	0	0	0	0	2,6
2005	6	0	0	0	0	0	0
2005	5	0	0	0	0	0	2,9
2005	5	0	0	0	0	0	0
2005	5	0	0	0	0	0	6,9
2005	5	0	1	1	0	0	9,1
2005	5	1	0	0	0	0	12,5
2005	5	0	0	0	0	0	0
2005	5	0	2	0	1	0	2,9
2005	5	1	0	0	0	0	0
2005	5	0	0	0	0	0	0
2005	5	1	0	0	0	0	2,6
2005	3	0	0	0	0	0	0
2005	3	0	0	0	0	0	6,3
2005	3	0	0	0	0	0	3,5
2005	3	0	0	0	0	0	14,3
2005	3	0	0	0	0	0	2,7
2005	3	0	2	0	1	0	0
2005	3	1	2	1	0	0	0
2005	3	0	0	0	0	0	4,5
2005	3	0	3	1	0	0	0
2005	3	0	2	1	0	0	0
2005	19	0	0	0	0	0	4,3
2005	19	0	0	0	0	0	4
2005	19	0	0	0	0	0	0
2005	19	0	0	0	0	0	0
2005	19	0	0	0	0	0	0

2005	19	0	0	0	0	0	0
2005	19	0	0	0	0	0	0
2005	19	0	0	0	0	0	5
2005	19	0	0	0	0	0	15,4
2005	19	0	0	0	0	0	0
2005	10	1	0	0	0	0	21,2
2005	18	0	0	0	0	0	0
2005	18	0	0	0	0	0	0
2005	18	0	0	0	0	0	0
2005	18	0	0	0	0	0	3,2
2005	18	0	0	0	0	0	0
2005	18	0	0	0	0	0	0
2005	18	0	0	0	0	0	0
2005	18	0	0	0	0	0	0
2005	18	0	0	0	0	0	0
2005	18	0	0	0	0	0	0
2005	18	0	0	0	0	0	0
2005	18	0	0	0	0	0	0
2005	18	0	0	0	0	0	0
2005	18	0	0	0	0	0	0
2005	18	0	0	0	0	0	0
2005	18	0	3	1	0	0	0
2005	18	0	0	0	0	0	4
2005	18	0	0	0	0	0	0
2005	18	0	0	0	0	0	0
2005	18	0	0	0	0	0	0
2005	38	0	0	0	0	0	0
2005	38	0	0	0	0	0	0
2005	38	0	0	0	0	0	0
2005	38	1	0	0	0	0	16,7
2005	38	0	0	0	0	0	0
2005	38	1	1	1	0	0	5,7
2005	38	0	0	0	0	0	9,1
2005	38	0	0	0	0	0	3,7
2005	38	0	0	0	0	0	0
2005	38	0	0	0	0	0	5,9
2005	22	0	0	0	0	0	0
2005	22	0	0	0	0	0	0
2005	22	0	0	0	0	0	0
2005	22	0	0	0	0	0	0
2005	22	0	0	0	0	0	0
2005	22	0	0	0	0	0	7,7
2005	22	0	0	0	0	0	0
2005	22	0	0	0	0	0	6,7
2005	22	0	0	0	0	0	0
2005	22	0	0	0	0	0	0
2005	42	0	0	0	0	0	0
2005	42	0	0	0	0	0	0
2005	42	1	0	0	0	0	8,1
2005	42	0	3	1	0	0	0
2005	42	0	0	0	0	0	0
2005	42	0	2	1	0	0	3
2005	42	1	0	0	0	0	0

2005	42	0	0	0	0	0	0
2005	42	0	2	1	0	0	0
2005	42	0	0	0	0	0	3
2005	8	0	2	1	0	0	0
2005	8	0	0	0	0	0	0
2005	8	0	0	0	0	0	0
2005	8	0	0	0	0	0	0
2005	8	0	0	0	0	0	0
2005	8	0	0	0	0	0	0
2005	8	0	0	0	0	0	0
2005	8	0	0	0	0	0	0
2005	8	0	0	0	0	0	0
2005	8	0	0	0	0	0	0
2005	16	0	0	0	0	0	0
2005	16	0	0	0	0	0	0
2005	16	0	0	0	0	0	1
2005	16	0	0	0	0	0	0
2005	16	0	1	1	0	0	2,1
2005	16	0	0	0	0	0	0
2005	16	0	0	0	0	0	0
2005	16	0	0	0	0	0	4,5
2005	16	0	0	0	0	0	0
2005	16	0	0	0	0	0	0
2005	24	0	0	0	0	0	4,3
2005	24	0	0	0	0	0	13,3
2005	24	0	0	0	0	0	13
2005	24	0	0	0	0	0	0
2005	24	0	0	0	0	0	3,1
2005	24	0	0	0	0	0	6,5
2005	24	1	0	0	0	0	42,5
2005	24	0	0	0	0	0	2,9
2005	24	1	0	0	0	0	11,8
2005	24	0	0	0	0	0	2,4
2005	26	0	0	0	0	0	3,2
2005	26	0	0	0	0	0	3
2005	26	0	0	0	0	0	0
2005	26	0	0	0	0	0	2
2005	26	0	0	0	0	0	0
2005	26	0	0	0	0	0	3,7
2005	26	0	0	0	0	0	4,2
2005	26	0	0	0	0	0	1
2005	26	0	0	0	0	0	14,1
2005	26	0	0	0	0	0	1
2005	29	0	0	0	0	0	11,8
2005	29	0	0	0	0	0	6,5
2005	29	0	0	0	0	0	0
2005	29	0	0	0	0	0	3,4
2005	29	0	0	0	0	0	3,1
2005	29	0	0	0	0	0	0
2005	29	0	0	0	0	0	0
2005	29	0	0	0	0	0	0
2005	29	0	0	0	0	0	8,8
2005	29	0	0	0	0	0	0

2005	15	0	0	0	0	0	0
2005	15	0	0	0	0	0	11,1
2005	15	1	0	0	0	0	0
2005	15	0	0	0	0	0	6,9
2005	15	0	0	0	0	0	9,4
2005	15	0	0	0	0	0	6,1
2005	15	0	0	0	0	0	3,1
2005	15	0	0	0	0	0	3,7
2005	15	0	0	0	0	0	12,9
2005	15	0	0	0	0	0	0
2005	43	0	0	0	0	0	8,3
2005	43	0	0	0	0	0	0
2005	43	0	3	0	1	0	6,5
2005	43	0	0	0	0	0	0
2005	43	0	3	1	0	0	0
2005	43	0	2	1	0	0	0
2005	43	0	0	0	0	0	6,1
2005	43	0	0	0	0	0	3,8
2005	43	0	2	1	0	0	0
2005	43	0	1	1	0	0	3,3
2005	17	1	0	0	0	0	5,6
2005	17	0	0	0	0	0	57,1
2005	17	0	0	0	0	0	9,7
2005	17	0	1	1	0	0	7,4
2005	17	1	0	0	0	0	11,8
2005	17	0	2	0	1	0	18,2
2005	17	0	0	0	0	0	12,1
2005	17	1	0	0	0	0	3,1
2005	17	1	0	0	0	0	6,7
2005	17	0	0	0	0	0	3,4
2005	12	0	0	0	0	0	1,3
2005	12	0	0	0	0	0	0,6
2005	12	0	0	0	0	0	0,3
2005	12	0	0	0	0	0	0,9
2005	12	0	0	0	0	0	0,7
2005	12	0	0	0	0	0	1,1
2006	13	0	0	0	0	0	2,5
2006	13	0	0	0	0	0	2
2006	13	0	0	0	0	0	0
2006	13	0	0	0	0	0	2,3
2006	13	0	0	0	0	0	0
2006	13	0	0	0	0	0	2,6
2006	13	0	0	0	0	0	3
2006	13	0	0	0	0	0	2,5
2006	13	0	0	0	0	0	4,1
2006	13	0	0	0	0	0	2
2006	1	0	0	0	0	0	0
2006	1	0	0	0	0	0	0
2006	1	0	0	0	0	0	0
2006	1	0	0	0	0	0	0
2006	1	0	0	0	0	0	0
2006	1	0	0	0	0	0	0
2006	1	0	0	0	0	0	0

2006	1	0	0	0	0	0	0
2006	1	0	0	0	0	0	0
2006	1	0	0	0	0	0	0
2006	41	0	1	1	0	0	34,1
2006	41	1	0	0	0	0	10
2006	41	0	0	0	0	0	56,25
2006	41	1	0	0	0	0	69,2
2006	41	0	0	0	0	0	15,6
2006	41	0	0	0	0	0	0,8
2006	41	0	0	0	0	0	27,8
2006	41	0	0	0	0	0	22,5
2006	41	0	0	0	0	0	8,6
2006	41	1	0	0	0	0	27,5
2006	23	0	0	0	0	0	4
2006	23	0	2	0	1	0	12
2006	23	0	0	0	0	0	8
2006	23	0	3	0	0	1	0
2006	23	0	0	0	0	0	2,5
2006	23	0	0	0	0	0	0
2006	23	0	0	0	0	0	7,5
2006	23	0	1	0	1	0	0
2006	23	0	2	1	0	0	4
2006	23	0	0	0	0	0	0
2006	6	0	0	0	0	0	0
2006	6	0	0	0	0	0	0
2006	6	0	0	0	0	0	0
2006	6	0	0	0	0	0	0
2006	6	0	2	1	0	0	0
2006	6	0	0	0	0	0	0
2006	6	0	0	0	0	0	0
2006	6	0	0	0	0	0	0
2006	6	0	0	0	0	0	0
2006	6	0	0	0	0	0	0
2006	5	0	0	0	0	0	0
2006	5	0	0	0	0	0	2
2006	5	0	0	0	0	0	6
2006	5	0	0	0	0	0	2
2006	5	1	0	0	0	0	6,7
2006	5	0	0	0	0	0	0
2006	5	0	0	0	0	0	6
2006	5	0	0	0	0	0	18
2006	5	0	0	0	0	0	2,5
2006	5	0	0	0	0	0	1
2006	3	0	2	1	0	0	0
2006	3	0	3	1	0	0	0
2006	3	0	1	1	0	0	6,7
2006	3	0	2	1	0	0	0
2006	3	0	0	0	0	0	0
2006	3	0	0	0	0	0	0
2006	3	0	0	0	0	0	0
2006	3	0	0	0	0	0	0
2006	3	0	0	0	0	0	0
2006	3	0	1	1	0	0	0

2006	18	0	0	0	0	0	0
2006	18	0	0	0	0	0	1,4
2006	18	0	0	0	0	0	0
2006	18	0	0	0	0	0	0
2006	18	0	0	0	0	0	0
2006	18	0	0	0	0	0	0
2006	18	0	0	0	0	0	0
2006	18	0	0	0	0	0	0
2006	18	0	0	0	0	0	0
2006	18	0	0	0	0	0	2,1
2006	18	0	0	0	0	0	0
2006	18	0	0	0	0	0	0
2006	18	0	0	0	0	0	0
2006	18	0	0	0	0	0	2,6
2006	18	0	0	0	0	0	5,1
2006	18	0	0	0	0	0	0
2006	18	0	0	0	0	0	0
2006	18	0	0	0	0	0	0
2006	18	0	0	0	0	0	2,5
2006	18	0	0	0	0	0	0
2006	38	0	0	0	0	0	0
2006	38	0	0	0	0	0	0
2006	38	0	0	0	0	0	0
2006	38	0	0	0	0	0	0
2006	38	0	0	0	0	0	0
2006	38	0	0	0	0	0	2,5
2006	38	0	0	0	0	0	0
2006	38	0	0	0	0	0	7,5
2006	38	0	0	0	0	0	0
2006	38	0	0	0	0	0	2,5
2006	22	0	0	0	0	0	0
2006	22	0	0	0	0	0	0
2006	22	0	1	1	0	0	2
2006	22	0	0	0	0	0	0
2006	22	0	0	0	0	0	0
2006	22	0	0	0	0	0	0
2006	22	1	0	0	0	0	6
2006	22	1	0	0	0	0	2
2006	22	1	0	0	0	0	0
2006	22	0	0	0	0	0	1,9
2006	42	0	0	0	0	0	0
2006	42	0	0	0	0	0	1,3
2006	42	1	0	0	0	0	6
2006	42	1	0	0	0	0	20
2006	42	1	0	0	0	0	6,4
2006	42	0	0	0	0	0	0
2006	42	0	0	0	0	0	4,3
2006	42	0	0	0	0	0	2,2
2006	42	0	0	0	0	0	8
2006	42	0	0	0	0	0	0
2006	8	0	0	0	0	0	0
2006	8	0	0	0	0	0	0
2006	8	0	0	0	0	0	0

2006	8	0	0	0	0	0	0
2006	8	0	0	0	0	0	0
2006	8	0	0	0	0	0	4
2006	8	0	0	0	0	0	0
2006	8	0	0	0	0	0	0
2006	8	0	0	0	0	0	0
2006	8	0	0	0	0	0	0
2006	16	0	0	0	0	0	0
2006	16	0	0	0	0	0	0
2006	16	0	0	0	0	0	0
2006	16	0	0	0	0	0	0
2006	16	0	0	0	0	0	0
2006	16	0	0	0	0	0	0
2006	16	0	0	0	0	0	0
2006	16	1	0	0	0	0	0
2006	16	0	0	0	0	0	0
2006	16	0	0	0	0	0	0
2006	24	0	1	1	0	0	5
2006	24	0	0	0	0	0	0
2006	24	0	0	0	0	0	5,6
2006	24	0	0	0	0	0	0
2006	24	0	0	0	0	0	2,5
2006	24	0	0	0	0	0	2,5
2006	24	0	0	0	0	0	0
2006	24	0	0	0	0	0	0
2006	24	0	0	0	0	0	0
2006	24	0	0	0	0	0	0
2006	26	0	0	0	0	0	0
2006	26	0	1	1	0	0	0
2006	26	0	0	0	0	0	0
2006	26	0	0	0	0	0	0
2006	26	0	0	0	0	0	0
2006	26	0	0	0	0	0	0
2006	26	0	0	0	0	0	0
2006	26	0	0	0	0	0	0
2006	26	0	0	0	0	0	0
2006	26	0	0	0	0	0	0
2006	29	0	0	0	0	0	0
2006	29	0	0	0	0	0	0
2006	29	0	0	0	0	0	5
2006	29	0	0	0	0	0	15
2006	29	0	0	0	0	0	2,5
2006	29	0	0	0	0	0	5
2006	29	0	0	0	0	0	5
2006	29	0	0	0	0	0	0
2006	29	0	0	0	0	0	0
2006	29	0	0	0	0	0	5
2006	15	0	0	0	0	0	0
2006	15	0	0	0	0	0	3,9
2006	15	0	2	1	0	0	6,8
2006	15	0	0	0	0	0	44,1
2006	15	0	0	0	0	0	5,3
2006	15	0	2	1	0	0	18,9

2006	15	0	0	0	0	0	1,9
2006	15	0	0	0	0	0	10
2006	15	0	0	0	0	0	10,6
2006	15	0	3	1	0	0	3,2
2006	43	0	0	0	0	0	0
2006	43	0	0	0	0	0	0
2006	43	0	0	0	0	0	0
2006	43	0	0	0	0	0	0
2006	43	0	0	0	0	0	0
2006	43	0	0	0	0	0	0
2006	43	0	0	0	0	0	0
2006	43	0	0	0	0	0	0
2006	43	0	0	0	0	0	0
2006	43	0	0	0	0	0	0
2006	17	0	0	0	0	0	10
2006	17	0	0	0	0	0	4
2006	17	0	0	0	0	0	2
2006	17	0	0	0	0	0	6,5
2006	17	0	0	0	0	0	0
2006	17	0	0	0	0	0	18,2
2006	17	0	0	0	0	0	0
2006	17	1	0	0	0	0	8,3
2006	17	0	0	0	0	0	34,3
2006	17	1	0	0	0	0	36,6
2006	20	0	0	0	0	0	0
2006	20	0	0	0	0	0	5
2006	20	0	0	0	0	0	0
2006	20	0	0	0	0	0	0
2006	20	0	0	0	0	0	0
2006	20	0	0	0	0	0	0
2006	19	0	0	0	0	0	0
2006	19	1	1	1	0	0	0
2006	19	0	0	0	0	0	0
2006	19	0	0	0	0	0	0
2006	19	0	0	0	0	0	0
2006	19	0	0	0	0	0	0
2006	19	0	0	0	0	0	0
2006	19	0	0	0	0	0	0
2006	19	0	0	0	0	0	0
2006	19	0	0	0	0	0	0
2006	12	0	0	0	0	0	1,7
2006	12	0	0	0	0	0	0
2006	12	0	0	0	0	0	0
2006	12	0	0	0	0	0	0
2006	12	0	0	0	0	0	0
2006	12	0	2	0	0	1	0
2006	12	0	0	0	0	0	2
2006	12	0	0	0	0	0	0
2006	12	0	2	0	1	0	0
2006	12	0	0	0	0	0	0
2007	13	0	0	0	0	0	0
2007	13	0	0	0	0	0	1,4
2007	13	0	0	0	0	0	0

2007	13	0	0	0	0	0	0
2007	13	0	0	0	0	0	0
2007	13	0	0	0	0	0	0
2007	13	0	0	0	0	0	0
2007	13	0	0	0	0	0	0
2007	13	0	0	0	0	0	0
2007	13	0	0	0	0	0	0
2007	1	0	0	0	0	0	0
2007	1	0	0	0	0	0	1,4
2007	1	0	0	0	0	0	1,6
2007	1	0	0	0	0	0	1,7
2007	1	0	0	0	0	0	2,7
2007	1	0	0	0	0	0	1,4
2007	1	0	0	0	0	0	4,5
2007	1	0	0	0	0	0	0
2007	1	0	0	0	0	0	1,8
2007	1	0	0	0	0	0	1,8
2007	41	0	0	0	0	0	1,9
2007	41	0	0	0	0	0	7,1
2007	41	0	0	0	0	0	1,1
2007	41	0	0	0	0	0	1,4
2007	41	0	0	0	0	0	0
2007	41	0	0	0	0	0	0
2007	41	0	0	0	0	0	5
2007	41	0	0	0	0	0	0
2007	41	0	0	0	0	0	0
2007	41	0	0	0	0	0	0
2007	23	1	0	0	0	0	8,5
2007	23	1	0	0	0	0	10
2007	23	1	0	0	0	0	10,5
2007	23	0	0	0	0	0	8,6
2007	23	1	0	0	0	0	18,9
2007	23	1	0	0	0	0	7,9
2007	23	1	0	0	0	0	10,5
2007	23	1	0	0	0	0	5,4
2007	23	1	0	0	0	0	14,1
2007	23	1	0	0	0	0	8,1
2007	6	0	1	0	0	1	1,1
2007	6	0	0	0	0	0	0
2007	6	0	2	0	1	0	0
2007	6	0	3	0	1	0	0
2007	6	1	0	0	0	0	8,9
2007	6	1	2	0	1	0	9,1
2007	6	0	0	0	0	0	1,6
2007	6	0	0	0	0	0	6,3
2007	6	0	3	1	0	0	2,9
2007	6	0	3	1	0	0	0
2007	5	0	0	0	0	0	0
2007	5	0	0	0	0	0	1,9
2007	5	0	0	0	0	0	1,8
2007	5	0	0	0	0	0	5,6
2007	5	0	0	0	0	0	16,2
2007	5	0	0	0	0	0	5,1

2007	5	0	0	0	0	0	0
2007	5	0	0	0	0	0	1,6
2007	5	1	0	0	0	0	24,4
2007	5	0	0	0	0	0	5,7
2007	3	0	0	0	0	0	5,8
2007	3	0	0	0	0	0	3,7
2007	3	0	0	0	0	0	4,5
2007	3	0	0	0	0	0	1,8
2007	3	0	0	0	0	0	9,1
2007	3	0	0	0	0	0	3,3
2007	3	0	0	0	0	0	7,3
2007	3	0	0	0	0	0	6,2
2007	3	1	0	0	0	0	1,6
2007	3	1	0	0	0	0	13,6
2007	18	0	0	0	0	0	0
2007	18	0	0	0	0	0	0
2007	18	0	0	0	0	0	0
2007	18	0	0	0	0	0	0
2007	18	0	0	0	0	0	0
2007	18	0	0	0	0	0	0
2007	18	0	0	0	0	0	0
2007	18	0	0	0	0	0	3,2
2007	18	0	2	1	0	0	0
2007	18	0	0	0	0	0	3,6
2007	22	0	0	0	0	0	0,8
2007	22	0	0	0	0	0	0
2007	22	0	0	0	0	0	0
2007	22	0	0	0	0	0	2
2007	22	0	0	0	0	0	0
2007	22	0	0	0	0	0	0
2007	22	0	0	0	0	0	0
2007	22	0	0	0	0	0	1,6
2007	22	0	0	0	0	0	1,3
2007	22	0	0	0	0	0	3,4
2007	42	0	0	0	0	0	10
2007	42	1	0	0	0	0	7,7
2007	42	1	0	0	0	0	9,4
2007	42	0	0	0	0	0	3,3
2007	42	0	0	0	0	0	0
2007	42	1	2	1	0	0	34,7
2007	42	1	0	0	0	0	13,9
2007	42	0	0	0	0	0	4,1
2007	42	0	0	0	0	0	3,9
2007	42	0	0	0	0	0	4,3
2007	8	0	0	0	0	0	5,7
2007	8	0	0	0	0	0	0
2007	8	0	0	0	0	0	0
2007	8	0	0	0	0	0	0
2007	8	0	0	0	0	0	0
2007	8	0	0	0	0	0	0
2007	8	0	0	0	0	0	0
2007	8	0	0	0	0	0	0
2007	8	0	0	0	0	0	0

2007	8	0	0	0	0	0	3,2
2007	16	0	0	0	0	0	0
2007	16	1	0	0	0	0	0
2007	16	0	0	0	0	0	0
2007	16	0	0	0	0	0	3,7
2007	16	0	0	0	0	0	1
2007	16	0	0	0	0	0	2
2007	16	0	0	0	0	0	1,2
2007	16	0	0	0	0	0	0
2007	16	0	1	1	0	0	4,8
2007	16	0	0	0	0	0	2,2
2007	38	0	0	0	0	0	17,7
2007	38	0	0	0	0	0	0
2007	38	0	0	0	0	0	0
2007	38	0	2	1	0	0	0
2007	38	1	0	0	0	0	13,3
2007	38	0	0	0	0	0	3,1
2007	38	0	0	0	0	0	4,5
2007	38	0	0	0	0	0	11,1
2007	38	1	0	0	0	0	1,9
2007	38	1	0	0	0	0	0
2007	24	0	0	0	0	0	6,5
2007	24	0	0	0	0	0	14,5
2007	24	1	0	0	0	0	32,1
2007	24	0	0	0	0	0	7,4
2007	24	1	0	0	0	0	9,3
2007	24	0	0	0	0	0	13,9
2007	24	0	0	0	0	0	5,2
2007	24	0	0	0	0	0	2
2007	24	0	0	0	0	0	12,9
2007	24	1	0	0	0	0	12,5
2007	26	0	0	0	0	0	0
2007	26	0	0	0	0	0	0
2007	26	0	0	0	0	0	0
2007	26	0	0	0	0	0	3
2007	26	0	0	0	0	0	0
2007	26	0	0	0	0	0	5,7
2007	26	0	0	0	0	0	0
2007	26	0	0	0	0	0	0
2007	26	0	0	0	0	0	5,2
2007	26	0	0	0	0	0	0
2007	29	0	1	0	1	0	29,6
2007	29	1	0	0	0	0	46
2007	29	1	0	0	0	0	28
2007	29	1	0	0	0	0	25,8
2007	29	1	0	0	0	0	29,1
2007	29	1	0	0	0	0	50,8
2007	29	0	0	0	0	0	2,6
2007	29	1	0	0	0	0	27,4
2007	29	0	0	0	0	0	31
2007	29	0	0	0	0	0	11,7
2007	15	0	0	0	0	0	1,8
2007	15	0	0	0	0	0	0

2007	15	0	0	0	0	0	0
2007	15	0	0	0	0	0	4,8
2007	15	0	0	0	0	0	0
2007	15	0	0	0	0	0	0
2007	15	0	0	0	0	0	0
2007	15	1	0	0	0	0	0
2007	15	0	0	0	0	0	0
2007	15	0	0	0	0	0	1,6
2007	43	0	0	0	0	0	0
2007	43	0	0	0	0	0	0
2007	43	0	0	0	0	0	0
2007	43	0	0	0	0	0	0
2007	43	0	0	0	0	0	0
2007	43	0	0	0	0	0	3,1
2007	43	0	0	0	0	0	2,2
2007	43	0	0	0	0	0	0
2007	43	0	0	0	0	0	1,4
2007	43	0	0	0	0	0	1,6
2007	17	0	0	0	0	0	0
2007	17	0	0	0	0	0	0
2007	17	1	0	0	0	0	0
2007	17	0	0	0	0	0	0
2007	17	0	0	0	0	0	0
2007	17	1	0	0	0	0	0
2007	17	0	0	0	0	0	0
2007	17	0	0	0	0	0	7,3
2007	17	0	0	0	0	0	0
2007	17	0	0	0	0	0	0
2007	20	1	1	1	0	0	2
2007	20	0	0	0	0	0	0
2007	20	0	0	0	0	0	0
2007	20	0	0	0	0	0	8,8
2007	20	0	0	0	0	0	13,9
2007	20	0	0	0	0	0	3,4
2007	20	0	0	0	0	0	0
2007	20	1	2	1	0	0	0
2007	20	1	0	0	0	0	10,9
2007	19	0	0	0	0	0	0
2007	19	0	0	0	0	0	3,8
2007	19	0	0	0	0	0	2,9
2007	19	0	0	0	0	0	6,7
2007	19	0	0	0	0	0	3,4
2007	19	0	0	0	0	0	6,4
2007	19	0	0	0	0	0	2,7
2007	19	1	0	0	0	0	0
2007	19	0	0	0	0	0	1,8
2007	19	0	0	0	0	0	3,8
2007	12	0	0	0	0	0	0
2007	12	0	0	0	0	0	0
2007	12	0	0	0	0	0	0
2007	12	0	0	0	0	0	0
2007	12	0	1	1	0	0	0
2007	12	0	0	0	0	0	0

2007	12	0	0	0	0	0	1
2007	12	0	0	0	0	0	2
2007	12	0	0	0	0	0	4,3
2007	12	0	3	1	0	0	0
2008	13	0	1	1	0	0	0
2008	13	0	0	0	0	0	0
2008	13	0	3	1	0	0	0
2008	13	0	0	0	0	0	0
2008	13	0	2	1	0	0	0
2008	13	0	0	0	0	0	0
2008	13	0	0	0	0	0	0
2008	13	0	3	1	0	0	0
2008	13	0	0	0	0	0	0
2008	13	0	0	0	0	0	0
2008	1	0	0	0	0	0	0
2008	1	0	0	0	0	0	0
2008	1	0	0	0	0	0	0
2008	1	0	0	0	0	0	0
2008	1	0	0	0	0	0	0
2008	1	0	0	0	0	0	0
2008	1	0	0	0	0	0	0
2008	1	0	0	0	0	0	0
2008	1	0	0	0	0	0	0
2008	1	0	0	0	0	0	0
2008	41	0	0	0	0	0	5,6
2008	41	0	0	0	0	0	13,3
2008	41	0	0	0	0	0	5
2008	41	0	0	0	0	0	6,6
2008	41	0	0	0	0	0	3,6
2008	41	0	0	0	0	0	1,3
2008	41	0	0	0	0	0	2,1
2008	41	0	0	0	0	0	111,6
2008	41	0	2	0	1	0	17,1
2008	41	0	0	0	0	0	0
2008	23	1	0	0	0	0	6,3
2008	23	1	0	0	0	0	11,7
2008	23	0	0	0	0	0	11,3
2008	23	0	0	0	0	0	24,7
2008	23	0	0	0	0	0	2,3
2008	23	0	0	0	0	0	6,8
2008	23	1	0	0	0	0	15,2
2008	23	1	0	0	0	0	3,3
2008	23	0	0	0	0	0	7,8
2008	23	0	0	0	0	0	0
2008	6	0	0	0	0	0	4,8
2008	6	0	0	0	0	0	4,5
2008	6	0	0	0	0	0	1,2
2008	6	0	0	0	0	0	0
2008	6	0	1	0	1	0	0
2008	6	0	0	0	0	0	5
2008	6	0	0	0	0	0	0
2008	6	0	0	0	0	0	0
2008	6	0	0	0	0	0	3

2008	6	0	0	0	0	0	0
2008	5	0	0	0	0	0	2,6
2008	5	0	0	0	0	0	0
2008	5	0	0	0	0	0	2,6
2008	5	0	0	0	0	0	1,1
2008	5	0	0	0	0	0	5,8
2008	5	0	0	0	0	0	12,3
2008	5	0	0	0	0	0	12,9
2008	5	0	0	0	0	0	5
2008	5	0	0	0	0	0	0
2008	5	0	0	0	0	0	3,2
2008	3	0	0	0	0	0	0
2008	3	0	0	0	0	0	0
2008	3	0	0	0	0	0	0
2008	3	0	0	0	0	0	3,6
2008	3	0	0	0	0	0	0
2008	3	0	0	0	0	0	0
2008	3	0	0	0	0	0	0
2008	3	0	0	0	0	0	1,1
2008	3	0	0	0	0	0	1,2
2008	3	0	0	0	0	0	0
2008	18	0	0	0	0	0	0
2008	18	0	0	0	0	0	3,7
2008	18	0	0	0	0	0	3,8
2008	18	0	0	0	0	0	4,4
2008	18	0	0	0	0	0	7,8
2008	18	0	0	0	0	0	53,7
2008	18	0	0	0	0	0	5,9
2008	18	0	0	0	0	0	12,5
2008	18	0	0	0	0	0	0
2008	18	1	0	0	0	0	3
2008	38	0	0	0	0	0	3,1
2008	38	0	0	0	0	0	0
2008	38	0	0	0	0	0	0
2008	38	1	0	0	0	0	0
2008	38	0	0	0	0	0	0
2008	38	0	0	0	0	0	0
2008	38	0	0	0	0	0	2,6
2008	38	0	0	0	0	0	0
2008	38	0	0	0	0	0	0
2008	38	0	0	0	0	0	1,7
2008	22	0	0	0	0	0	0
2008	22	0	0	0	0	0	0
2008	22	0	0	0	0	0	0
2008	22	0	0	0	0	0	0
2008	22	0	0	0	0	0	0
2008	22	0	0	0	0	0	0
2008	22	0	0	0	0	0	0
2008	22	0	0	0	0	0	0
2008	22	0	0	0	0	0	0
2008	22	0	0	0	0	0	0
2008	42	0	0	0	0	0	0
2008	42	0	0	0	0	0	0

2008	42	0	0	0	0	0	0
2008	42	0	0	0	0	0	0
2008	42	0	2	1	0	0	0
2008	42	0	0	0	0	0	0
2008	42	0	0	0	0	0	0
2008	42	0	0	0	0	0	0
2008	42	0	0	0	0	0	0
2008	42	0	0	0	0	0	1,4
2008	8	0	0	0	0	0	0
2008	8	0	0	0	0	0	0
2008	8	0	0	0	0	0	0
2008	8	0	0	0	0	0	0
2008	8	0	0	0	0	0	0
2008	8	0	0	0	0	0	0
2008	8	0	0	0	0	0	0
2008	8	0	0	0	0	0	0
2008	8	0	0	0	0	0	0
2008	8	0	0	0	0	0	1,3
2008	16	0	0	0	0	0	0
2008	16	0	0	0	0	0	0
2008	16	0	0	0	0	0	0
2008	16	0	0	0	0	0	0
2008	16	0	0	0	0	0	0
2008	16	0	0	0	0	0	1,2
2008	16	0	0	0	0	0	0
2008	16	0	0	0	0	0	7
2008	16	0	0	0	0	0	2
2008	16	0	0	0	0	0	0
2008	24	0	0	0	0	0	12,8
2008	24	0	0	0	0	0	1,4
2008	24	0	0	0	0	0	0
2008	24	0	0	0	0	0	0
2008	24	0	0	0	0	0	23,2
2008	24	0	0	0	0	0	0
2008	24	0	0	0	0	0	0
2008	24	0	0	0	0	0	0
2008	24	0	2	0	1	0	17,9
2008	24	0	0	0	0	0	0
2008	26	1	0	0	0	0	5,7
2008	26	0	0	0	0	0	0
2008	26	0	0	0	0	0	0
2008	26	0	0	0	0	0	0
2008	26	1	0	0	0	0	1,2
2008	26	0	0	0	0	0	0
2008	26	0	0	0	0	0	0
2008	26	0	0	0	0	0	0
2008	26	0	0	0	0	0	0
2008	26	0	0	0	0	0	0
2008	15	0	0	0	0	0	26,9
2008	15	0	0	0	0	0	0
2008	15	0	0	0	0	0	0
2008	15	0	0	0	0	0	0
2008	15	0	0	0	0	0	0

2008	15	0	0	0	0	0	0
2008	15	0	0	0	0	0	0
2008	15	0	0	0	0	0	0
2008	15	0	0	0	0	0	0
2008	15	0	0	0	0	0	0
2008	43	0	0	0	0	0	0
2008	43	0	0	0	0	0	3,6
2008	43	0	0	0	0	0	0
2008	43	0	0	0	0	0	1,8
2008	43	0	0	0	0	0	3,3
2008	43	0	0	0	0	0	4,2
2008	43	0	0	0	0	0	1,7
2008	43	0	0	0	0	0	0
2008	43	0	0	0	0	0	0
2008	43	0	0	0	0	0	1,9
2008	17	0	0	0	0	0	0
2008	17	0	0	0	0	0	1,3
2008	17	0	0	0	0	0	0
2008	17	0	0	0	0	0	0
2008	17	0	0	0	0	0	0
2008	17	0	0	0	0	0	1,5
2008	17	0	0	0	0	0	0
2008	17	0	0	0	0	0	3,8
2008	17	0	0	0	0	0	5,8
2008	17	0	0	0	0	0	0
2008	20	0	0	0	0	0	0
2008	20	1	0	0	0	0	5,6
2008	20	0	0	0	0	0	1,4
2008	20	0	0	0	0	0	1,9
2008	20	0	0	0	0	0	0
2008	20	0	0	0	0	0	0
2008	20	0	0	0	0	0	1,1
2008	20	0	0	0	0	0	1,6
2008	20	0	0	0	0	0	2,5
2008	20	0	0	0	0	0	0
2008	19	0	0	0	0	0	2,5
2008	19	0	2	0	1	0	17,7
2008	19	0	0	0	0	0	1,1
2008	19	0	0	0	0	0	2,1
2008	19	0	0	0	0	0	10,2
2008	19	1	0	0	0	0	18,2
2008	19	0	0	0	0	0	0
2008	19	0	0	0	0	0	0
2008	19	0	0	0	0	0	6,4
2008	19	0	0	0	0	0	12,7
2008	12	0	0	0	0	0	7,3
2008	12	0	0	0	0	0	9,5
2008	12	0	0	0	0	0	3,3
2008	12	0	0	0	0	0	2,7
2008	12	0	0	0	0	0	5,9
2008	12	0	0	0	0	0	16,1
2008	12	0	0	0	0	0	16
2008	12	0	0	0	0	0	2,8

2008	12	0	2	0	1	0	12,2
2008	12	0	2	1	0	0	2,4
2009	13	0	1	1	0	0	0
2009	13	0	0	0	0	0	0
2009	13	0	0	0	0	0	0
2009	13	0	0	0	0	0	1,1
2009	13	0	0	0	0	0	0
2009	13	0	0	0	0	0	0
2009	13	0	0	0	0	0	0
2009	13	0	0	0	0	0	0
2009	13	0	0	0	0	0	0
2009	13	0	0	0	0	0	1,3
2009	1	0	0	0	0	0	0
2009	1	0	0	0	0	0	0
2009	1	0	0	0	0	0	3,2
2009	1	0	0	0	0	0	0
2009	1	0	0	0	0	0	0
2009	1	0	0	0	0	0	0
2009	1	0	0	0	0	0	0
2009	1	0	0	0	0	0	0
2009	1	0	0	0	0	0	0
2009	1	0	0	0	0	0	0
2009	5	1	0	0	0	0	104,4
2009	5	0	0	0	0	0	0
2009	5	0	0	0	0	0	4,1
2009	5	0	0	0	0	0	1,7
2009	5	1	0	0	0	0	14,4
2009	5	0	0	0	0	0	9,3
2009	5	1	0	0	0	0	2,8
2009	5	1	0	0	0	0	22,1
2009	5	1	0	0	0	0	4,2
2009	5	0	0	0	0	0	7,8
2009	3	0	0	0	0	0	0
2009	3	0	0	0	0	0	0
2009	3	0	0	0	0	0	0
2009	3	0	0	0	0	0	0
2009	3	1	0	0	0	0	0
2009	3	0	0	0	0	0	0
2009	3	0	0	0	0	0	0
2009	3	0	0	0	0	0	0
2009	3	0	0	0	0	0	0
2009	3	0	0	0	0	0	0
2009	18	0	0	0	0	0	2,3
2009	18	0	0	0	0	0	25,4
2009	18	0	2	1	0	0	4
2009	18	0	0	0	0	0	1
2009	18	0	0	0	0	0	7,8
2009	18	0	0	0	0	0	9,6
2009	18	0	0	0	0	0	4,1
2009	18	0	0	0	0	0	5
2009	18	0	0	0	0	0	0
2009	18	0	0	0	0	0	3,1
2009	38	1	0	0	0	0	30

2009	38	0	0	0	0	0	7,8
2009	38	1	0	0	0	0	16,9
2009	38	0	0	0	0	0	21,8
2009	38	0	0	0	0	0	13,2
2009	38	1	0	0	0	0	11,8
2009	38	1	0	0	0	0	14,1
2009	38	1	0	0	0	0	30,6
2009	38	1	0	0	0	0	18,2
2009	38	0	0	0	0	0	6,1
2009	22	1	1	1	0	0	8
2009	22	0	0	0	0	0	5,4
2009	22	0	1	1	0	0	0,8
2009	22	1	0	0	0	0	14,6
2009	22	0	0	0	0	0	4,4
2009	22	1	1	1	0	0	28,4
2009	22	0	0	0	0	0	3,8
2009	22	1	0	0	0	0	24,4
2009	22	0	0	0	0	0	2
2009	22	0	0	0	0	0	10,7
2009	42	0	0	0	0	0	0
2009	42	0	0	0	0	0	4,7
2009	42	0	0	0	0	0	0
2009	42	0	0	0	0	0	2,1
2009	42	0	0	0	0	0	0,9
2009	42	1	0	0	0	0	13,5
2009	42	0	0	0	0	0	2,6
2009	42	0	0	0	0	0	2,8
2009	42	0	0	0	0	0	2,1
2009	42	0	0	0	0	0	0,9
2009	8	0	0	0	0	0	5,1
2009	8	0	0	0	0	0	0
2009	8	0	0	0	0	0	1,6
2009	8	0	0	0	0	0	0
2009	8	0	0	0	0	0	0
2009	8	0	0	0	0	0	1,5
2009	8	0	0	0	0	0	0
2009	8	0	0	0	0	0	0
2009	8	0	0	0	0	0	0
2009	8	0	2	1	0	0	0
2009	16	0	0	0	0	0	0
2009	16	0	0	0	0	0	0
2009	16	0	0	0	0	0	0
2009	16	0	0	0	0	0	0
2009	16	0	0	0	0	0	0
2009	16	0	0	0	0	0	1,4
2009	16	0	0	0	0	0	0
2009	16	0	0	0	0	0	0
2009	16	0	0	0	0	0	0
2009	16	0	0	0	0	0	0
2009	24	0	0	0	0	0	0
2009	24	0	0	0	0	0	8,2
2009	24	0	0	0	0	0	1,3
2009	24	0	0	0	0	0	2,8

2009	24	0	0	0	0	0	1,3
2009	24	0	0	0	0	0	16,8
2009	24	0	0	0	0	0	1,4
2009	24	0	0	0	0	0	1,2
2009	24	0	0	0	0	0	0
2009	24	0	0	0	0	0	4,7
2009	26	0	0	0	0	0	18,2
2009	26	1	0	0	0	0	17
2009	26	1	0	0	0	0	24,2
2009	26	1	0	0	0	0	40
2009	26	0	0	0	0	0	27,5
2009	26	1	0	0	0	0	39,6
2009	26	1	0	0	0	0	30,4
2009	26	0	0	0	0	0	23,7
2009	26	1	0	0	0	0	56,7
2009	26	0	0	0	0	0	55,1
2009	15	0	0	0	0	0	0
2009	15	0	3	1	0	0	0
2009	15	0	0	0	0	0	3,2
2009	15	0	0	0	0	0	1,8
2009	15	0	2	1	0	0	0
2009	15	0	0	0	0	0	0
2009	15	0	2	1	0	0	2,3
2009	15	0	0	0	0	0	6,8
2009	15	0	0	0	0	0	1,8
2009	15	0	0	0	0	0	11,6
2009	43	0	0	0	0	0	9,1
2009	43	0	0	0	0	0	0
2009	43	0	0	0	0	0	1,8
2009	43	0	0	0	0	0	9,2
2009	43	0	0	0	0	0	1,3
2009	43	0	0	0	0	0	7,1
2009	43	0	0	0	0	0	0
2009	43	0	0	0	0	0	8,3
2009	43	0	0	0	0	0	2,7
2009	43	0	0	0	0	0	5,8
2009	17	0	0	0	0	0	4,2
2009	17	0	0	0	0	0	0
2009	17	1	0	0	0	0	4,9
2009	17	0	0	0	0	0	13,6
2009	17	1	0	0	0	0	5,7
2009	17	0	0	0	0	0	8,5
2009	17	0	0	0	0	0	3,3
2009	17	1	0	0	0	0	4
2009	17	0	0	0	0	0	9,3
2009	17	0	0	0	0	0	1,1
2009	20	0	0	0	0	0	6,5
2009	20	0	0	0	0	0	0
2009	20	0	0	0	0	0	1,5
2009	20	0	0	0	0	0	5,6
2009	20	1	0	0	0	0	40,9
2009	20	1	0	0	0	0	0
2009	20	0	0	0	0	0	1,5

2009	20	0	0	0	0	0	4,2
2009	20	0	0	0	0	0	21,4
2009	20	0	0	0	0	0	18,5
2009	19	0	0	0	0	0	15,1
2009	19	0	0	0	0	0	8,5
2009	19	0	0	0	0	0	1,5
2009	19	0	0	0	0	0	0,9
2009	19	0	0	0	0	0	14
2009	19	0	0	0	0	0	0
2009	19	0	0	0	0	0	1,5
2009	19	0	0	0	0	0	0,8
2009	19	0	0	0	0	0	0
2009	19	0	0	0	0	0	4,8
2009	12	0	0	0	0	0	2,8
2009	12	1	1	0	1	0	5,4
2009	12	0	3	0	1	0	0
2009	12	0	3	0	1	0	3,7
2009	12	0	2	0	1	0	0
2009	12	0	3	0	1	0	0
2009	12	0	0	0	0	0	0
2009	12	0	3	0	1	0	0
2009	12	0	3	0	1	0	0
2009	12	0	0	0	0	0	0
2010	13	0	0	0	0	0	6,7
2010	13	0	0	0	0	0	15,7
2010	13	0	0	0	0	0	8,8
2010	13	1	0	0	0	0	9,4
2010	13	1	0	0	0	0	2,7
2010	13	1	0	0	0	0	11
2010	13	0	0	0	0	0	0
2010	13	0	0	0	0	0	2,9
2010	13	0	0	0	0	0	1,2
2010	13	1	0	0	0	0	16,7
2010	14	0	0	0	0	0	2,5
2010	14	0	0	0	0	0	1,4
2010	14	0	0	0	0	0	5,3
2010	14	0	0	0	0	0	14,2
2010	14	0	0	0	0	0	0
2010	14	0	0	0	0	0	8,8
2010	14	0	2	0	1	0	4,5
2010	14	0	0	0	0	0	0
2010	14	0	2	1	0	0	35,6
2010	14	0	0	0	0	0	9,2
2010	1	0	0	0	0	0	15,9
2010	1	0	0	0	0	0	0,9
2010	1	0	0	0	0	0	0
2010	1	0	0	0	0	0	0
2010	1	0	0	0	0	0	0
2010	1	0	0	0	0	0	0,9
2010	1	0	0	0	0	0	0
2010	1	0	0	0	0	0	0,9
2010	1	0	0	0	0	0	0
2010	1	0	0	0	0	0	2,8

2010	9	0	0	0	0	0	0
2010	9	0	0	0	0	0	0
2010	9	1	0	0	0	0	1,6
2010	9	0	0	0	0	0	0
2010	9	0	0	0	0	0	0,7
2010	9	0	0	0	0	0	0
2010	9	0	0	0	0	0	0
2010	9	0	0	0	0	0	0
2010	9	0	0	0	0	0	0
2010	9	0	0	0	0	0	0
2010	27	0	0	0	0	0	5,4
2010	27	1	0	0	0	0	7,3
2010	27	1	0	0	0	0	5,2
2010	27	1	0	0	0	0	8,1
2010	27	0	2	0	1	0	1,9
2010	27	0	2	0	1	0	1,8
2010	27	1	2	0	1	0	9,5
2010	27	1	2	0	1	0	1,1
2010	27	0	2	0	1	0	2,7
2010	27	1	0	0	0	0	3,1
2010	5	1	0	0	0	0	19,6
2010	5	0	0	0	0	0	5,4
2010	5	1	0	0	0	0	5,1
2010	5	0	1	0	1	0	0
2010	5	1	2	0	1	0	6,7
2010	5	0	0	0	0	0	3,1
2010	5	0	0	0	0	0	2,2
2010	5	0	0	0	0	0	0
2010	5	0	0	0	0	0	1,1
2010	5	0	0	0	0	0	0
2010	3	0	0	0	0	0	2,6
2010	3	0	0	0	0	0	4
2010	3	0	0	0	0	0	1,3
2010	3	0	0	0	0	0	0,9
2010	3	0	0	0	0	0	5,7
2010	3	0	0	0	0	0	1,6
2010	3	0	0	0	0	0	1,3
2010	3	0	0	0	0	0	0
2010	3	0	0	0	0	0	2,7
2010	3	0	0	0	0	0	2,8
2010	7	0	1	0	1	0	0
2010	7	0	2	0	1	0	2,8
2010	7	0	0	0	0	0	0
2010	7	0	0	0	0	0	0
2010	7	0	0	0	0	0	1,3
2010	7	0	0	0	0	0	0
2010	7	0	0	0	0	0	0
2010	7	0	0	0	0	0	0
2010	7	0	0	0	0	0	0
2010	7	0	3	0	1	0	0
2010	18	0	0	0	0	0	0
2010	18	0	0	0	0	0	0
2010	18	0	0	0	0	0	0,9

2010	18	0	0	0	0	0	0
2010	18	0	0	0	0	0	0
2010	18	0	0	0	0	0	0
2010	18	0	0	0	0	0	0
2010	18	0	0	0	0	0	0
2010	18	0	0	0	0	0	0
2010	18	0	0	0	0	0	0
2010	38	0	0	0	0	0	3,6
2010	38	0	0	0	0	0	7,5
2010	38	0	0	0	0	0	3,8
2010	38	1	0	0	0	0	25,6
2010	38	0	0	0	0	0	0
2010	38	0	0	0	0	0	2,1
2010	38	0	2	1	0	0	3,4
2010	38	0	0	0	0	0	1,2
2010	38	0	0	0	0	0	3,2
2010	38	0	0	0	0	0	1
2010	42	0	0	0	0	0	1,5
2010	42	0	0	0	0	0	0
2010	42	1	0	0	0	0	1,1
2010	42	1	0	0	0	0	2,3
2010	42	0	2	1	0	0	2,7
2010	42	0	0	0	0	0	0
2010	42	0	0	0	0	0	0
2010	42	0	0	0	0	0	1,4
2010	42	0	0	0	0	0	1,4
2010	42	1	0	0	0	0	0
2010	11	0	0	0	0	0	0
2010	11	0	0	0	0	0	1,2
2010	11	0	0	0	0	0	10,3
2010	11	0	0	0	0	0	8,3
2010	11	1	0	0	0	0	15,3
2010	11	1	0	0	0	0	12,4
2010	11	1	0	0	0	0	1,3
2010	11	0	0	0	0	0	1,3
2010	11	1	0	0	0	0	3,1
2010	11	0	0	0	0	0	6,8
2010	37	0	0	0	0	0	0
2010	37	0	0	0	0	0	0
2010	37	0	0	0	0	0	0
2010	37	0	0	0	0	0	0
2010	37	0	0	0	0	0	0
2010	37	0	0	0	0	0	0
2010	37	0	0	0	0	0	0
2010	37	0	0	0	0	0	0
2010	37	0	0	0	0	0	6,1
2010	37	0	0	0	0	0	0
2010	8	0	0	0	0	0	0
2010	8	0	0	0	0	0	0
2010	8	0	0	0	0	0	0
2010	8	0	0	0	0	0	2,2
2010	8	0	0	0	0	0	0
2010	8	0	0	0	0	0	0

2010	8	0	0	0	0	0	0
2010	8	0	0	0	0	0	0
2010	8	0	0	0	0	0	0
2010	8	0	0	0	0	0	5,7
2010	16	0	0	0	0	0	0
2010	16	0	0	0	0	0	0
2010	16	0	0	0	0	0	0
2010	16	0	0	0	0	0	1,8
2010	16	0	0	0	0	0	0
2010	16	0	0	0	0	0	0
2010	16	0	0	0	0	0	1
2010	16	0	0	0	0	0	0
2010	16	0	0	0	0	0	0
2010	16	0	0	0	0	0	0,9
2010	31	0	2	0	1	0	0
2010	31	0	0	0	0	0	0
2010	31	0	0	0	0	0	0
2010	31	0	0	0	0	0	0
2010	31	0	0	0	0	0	0
2010	31	0	0	0	0	0	0
2010	31	0	0	0	0	0	0
2010	31	0	0	0	0	0	0
2010	31	0	0	0	0	0	0
2010	31	0	1	0	1	0	0
2010	26	0	0	0	0	0	0
2010	26	0	0	0	0	0	0
2010	26	0	0	0	0	0	0
2010	26	0	0	0	0	0	1,4
2010	26	0	0	0	0	0	0,8
2010	26	0	0	0	0	0	0
2010	26	0	0	0	0	0	0
2010	26	0	0	0	0	0	0,8
2010	26	0	0	0	0	0	0
2010	26	0	0	0	0	0	0
2010	24	0	2	1	0	0	5,4
2010	24	1	0	0	0	0	18,7
2010	24	1	1	0	1	0	50
2010	24	0	0	0	0	0	1,6
2010	24	1	0	0	0	0	38,7
2010	24	0	0	0	0	0	15,7
2010	24	0	0	0	0	0	6,3
2010	24	0	0	0	0	0	2,9
2010	24	0	0	0	0	0	9,1
2010	15	0	0	0	0	0	5,7
2010	15	1	0	0	0	0	0
2010	15	0	0	0	0	0	0
2010	15	0	3	1	0	0	0
2010	15	0	2	1	0	0	1,9
2010	15	0	0	0	0	0	1,6
2010	15	0	2	1	0	0	3,9
2010	15	0	1	1	0	0	0
2010	15	0	0	0	0	0	5,2
2010	15	0	1	1	0	0	2,9

2010	43	0	0	0	0	0	1,2
2010	43	0	0	0	0	0	2,1
2010	43	0	0	0	0	0	1
2010	43	0	0	0	0	0	0
2010	43	0	0	0	0	0	0
2010	43	0	0	0	0	0	0
2010	43	0	0	0	0	0	0
2010	43	0	0	0	0	0	0
2010	43	0	0	0	0	0	1,1
2010	43	0	3	0	1	0	0
2010	17	1	0	0	0	0	32,2
2010	17	1	0	0	0	0	20,8
2010	17	0	0	0	0	0	9,5
2010	17	1	0	0	0	0	25,9
2010	17	1	0	0	0	0	27,3
2010	17	0	0	0	0	0	2,5
2010	17	0	0	0	0	0	0
2010	17	0	0	0	0	0	4,1
2010	32	0	0	0	0	0	2,5
2010	32	0	0	0	0	0	11,3
2010	32	0	0	0	0	0	6,3
2010	32	0	2	0	1	0	7,5
2010	32	0	0	0	0	0	23,1
2010	32	0	0	0	0	0	1,4
2010	32	0	0	0	0	0	10,4
2010	32	0	0	0	0	0	8,6
2010	32	0	0	0	0	0	1,3
2010	32	1	0	0	0	0	21,4
2010	20	0	0	0	0	0	11,8
2010	20	0	0	0	0	0	2,8
2010	20	0	0	0	0	0	2,9
2010	20	0	0	0	0	0	0
2010	20	0	0	0	0	0	0
2010	20	0	0	0	0	0	9
2010	20	0	0	0	0	0	1,4
2010	20	1	2	1	0	0	23,2
2010	20	0	0	0	0	0	4
2010	20	1	0	0	0	0	23,8
2010	19	1	0	0	0	0	0
2010	19	0	0	0	0	0	0,7
2010	19	0	0	0	0	0	0
2010	19	0	0	0	0	0	0,8
2010	19	0	0	0	0	0	0
2010	19	0	0	0	0	0	1,6
2010	19	0	0	0	0	0	0
2010	19	0	0	0	0	0	0,7
2010	19	0	0	0	0	0	0,7
2010	19	0	0	0	0	0	1,7
2010	12	0	0	0	0	0	0
2010	12	0	0	0	0	0	1,6
2010	12	0	0	0	0	0	5,1
2010	12	0	0	0	0	0	0
2010	12	0	0	0	0	0	6,7

2010	12	0	0	0	0	0	0
2010	12	0	0	0	0	0	0
2010	12	0	0	0	0	0	0
2010	12	0	0	0	0	0	0
2010	12	0	0	0	0	0	3,7
2011	13	0	0	0	0	0	8
2011	13	0	0	0	0	0	0,9
2011	13	0	0	0	0	0	0
2011	13	0	0	0	0	0	3,7
2011	13	0	1	1	0	0	3,7
2011	13	0	0	0	0	0	3,3
2011	13	0	0	0	0	0	4,7
2011	13	0	0	0	0	0	0
2011	13	0	0	0	0	0	2,8
2011	13	0	0	0	0	0	4,4
2011	14	1	0	0	0	0	1,6
2011	14	0	0	0	0	0	6,9
2011	14	0	2	0	0	1	0,8
2011	14	1	2	0	0	1	1,9
2011	14	0	0	0	0	0	0
2011	14	1	0	0	0	0	7,8
2011	14	0	0	0	0	0	0,7
2011	14	1	2	0	1	0	45,8
2011	14	0	1	1	0	0	0
2011	14	1	1	1	0	0	0,7
2011	1	1	0	0	0	0	15,9
2011	1	1	0	0	0	0	40,3
2011	1	0	0	0	0	0	1,8
2011	1	1	0	0	0	0	29,4
2011	1	0	0	0	0	0	2,9
2011	1	1	0	0	0	0	5,6
2011	1	1	0	0	0	0	7,8
2011	1	1	0	0	0	0	40
2011	1	1	0	0	0	0	2,8
2011	1	0	0	0	0	0	5,9
2011	9	0	0	0	0	0	0,7
2011	9	0	0	0	0	0	0,8
2011	9	0	0	0	0	0	0
2011	9	0	0	0	0	0	0,8
2011	9	0	0	0	0	0	0
2011	9	0	0	0	0	0	0
2011	9	0	2	1	0	0	0
2011	9	0	0	0	0	0	0
2011	9	0	0	0	0	0	0
2011	27	0	1	0	1	0	7,3
2011	27	0	0	0	0	0	2
2011	27	0	0	0	0	0	0,9
2011	27	0	0	0	0	0	10,6
2011	27	1	0	0	0	0	5
2011	27	0	3	0	1	0	0
2011	27	0	0	0	0	0	2,9
2011	27	0	0	0	0	0	3,1
2011	27	0	0	0	0	0	13,5

2011	27	1	0	0	0	0	3,4
2011	30	0	0	0	0	0	1,4
2011	30	0	0	0	0	0	0
2011	30	1	0	0	0	0	7,5
2011	30	0	0	0	0	0	0
2011	30	0	0	0	0	0	2,1
2011	30	1	0	0	0	0	1,7
2011	30	0	1	0	1	0	40,7
2011	30	0	0	0	0	0	20,9
2011	30	0	0	0	0	0	1,6
2011	30	0	0	0	0	0	1,7
2011	5	0	0	0	0	0	12,2
2011	5	0	0	0	0	0	4,5
2011	5	0	0	0	0	0	3,3
2011	5	0	0	0	0	0	5,2
2011	5	0	0	0	0	0	5,4
2011	5	1	0	0	0	0	11,1
2011	5	0	0	0	0	0	47
2011	5	1	0	0	0	0	3,1
2011	5	0	0	0	0	0	10,3
2011	5	1	0	0	0	0	4,5
2011	3	0	0	0	0	0	1,7
2011	3	0	0	0	0	0	4,2
2011	3	0	0	0	0	0	0
2011	3	1	0	0	0	0	22,8
2011	3	0	0	0	0	0	1,6
2011	3	0	0	0	0	0	4
2011	3	1	0	0	0	0	5,7
2011	3	0	2	0	0	1	0
2011	3	0	0	0	0	0	0
2011	3	0	0	0	0	0	22,9
2011	7	1	0	0	0	0	0,9
2011	7	0	0	0	0	0	0,8
2011	7	0	0	0	0	0	1,8
2011	7	0	0	0	0	0	6,1
2011	7	0	0	0	0	0	1
2011	7	0	0	0	0	0	0
2011	7	0	0	0	0	0	2
2011	7	1	3	0	1	0	1,8
2011	7	0	0	0	0	0	0
2011	7	0	0	0	0	0	0,9
2011	18	0	0	0	0	0	0
2011	18	0	0	0	0	0	1,4
2011	18	0	0	0	0	0	0,7
2011	18	0	0	0	0	0	2,7
2011	18	1	0	0	0	0	3,2
2011	18	0	0	0	0	0	2
2011	18	0	0	0	0	0	6,2
2011	18	0	0	0	0	0	2,4
2011	18	1	0	0	0	0	2,7
2011	18	0	0	0	0	0	0,7
2011	38	1	0	0	0	0	20
2011	38	1	0	0	0	0	27,8

2011	38	1	0	0	0	0	36,7
2011	38	1	0	0	0	0	69,3
2011	38	1	0	0	0	0	53
2011	38	1	0	0	0	0	28,6
2011	38	0	0	0	0	0	0,7
2011	38	1	0	0	0	0	48,3
2011	38	1	0	0	0	0	8,8
2011	38	1	0	0	0	0	20,9
2011	42	0	0	0	0	0	4,5
2011	42	0	0	0	0	0	13,3
2011	42	0	0	0	0	0	0
2011	42	0	0	0	0	0	0
2011	42	0	0	0	0	0	1,6
2011	42	0	0	0	0	0	6,5
2011	42	0	0	0	0	0	0,7
2011	42	0	0	0	0	0	0
2011	42	0	0	0	0	0	2,9
2011	42	0	0	0	0	0	0
2011	11	0	0	0	0	0	0
2011	11	1	0	0	0	0	21,6
2011	11	0	0	0	0	0	4,4
2011	11	0	0	0	0	0	0,8
2011	11	0	0	0	0	0	2,2
2011	11	0	0	0	0	0	2,5
2011	11	1	0	0	0	0	2,1
2011	11	0	0	0	0	0	4,9
2011	11	0	0	0	0	0	1,7
2011	11	1	0	0	0	0	3,4
2011	37	0	0	0	0	0	0
2011	37	0	0	0	0	0	0,7
2011	37	1	0	0	0	0	2,3
2011	37	0	0	0	0	0	2,4
2011	37	0	0	0	0	0	1,5
2011	37	0	0	0	0	0	2
2011	37	1	0	0	0	0	7,4
2011	37	0	0	0	0	0	0
2011	37	1	0	0	0	0	2,8
2011	37	0	0	0	0	0	5,4
2011	8	0	0	0	0	0	0
2011	8	0	0	0	0	0	1,1
2011	8	0	0	0	0	0	0
2011	8	0	2	0	1	0	0
2011	8	0	1	0	1	0	0
2011	8	0	0	0	0	0	0
2011	8	0	0	0	0	0	0
2011	8	0	0	0	0	0	0,8
2011	8	0	0	0	0	0	2,1
2011	8	0	0	0	0	0	0
2011	16	0	0	0	0	0	1,6
2011	16	0	0	0	0	0	0,9
2011	16	0	0	0	0	0	3,6
2011	16	0	0	0	0	0	3
2011	16	0	0	0	0	0	0

2011	16	0	0	0	0	0	0
2011	16	1	0	0	0	0	4
2011	16	1	0	0	0	0	9,3
2011	16	0	0	0	0	0	1,7
2011	16	0	0	0	0	0	1
2011	31	0	2	0	0	1	0
2011	31	0	0	0	0	0	0
2011	31	0	0	0	0	0	1
2011	31	0	0	0	0	0	0,7
2011	31	0	0	0	0	0	0
2011	31	0	0	0	0	0	6,7
2011	31	0	0	0	0	0	0
2011	31	0	0	0	0	0	0
2011	31	0	0	0	0	0	2,5
2011	31	0	0	0	0	0	2,5
2011	26	0	0	0	0	0	0
2011	26	0	0	0	0	0	0
2011	26	0	0	0	0	0	2,1
2011	26	0	0	0	0	0	2
2011	26	0	0	0	0	0	0
2011	26	0	0	0	0	0	1,1
2011	26	0	0	0	0	0	1,7
2011	26	0	0	0	0	0	2,7
2011	26	0	0	0	0	0	0
2011	26	1	0	0	0	0	0,9
2011	24	1	0	0	0	0	5,9
2011	24	0	0	0	0	0	0,8
2011	24	0	0	0	0	0	1,1
2011	24	0	0	0	0	0	0
2011	24	1	0	0	0	0	10,8
2011	24	0	2	1	0	0	0
2011	24	0	0	0	0	0	3,4
2011	24	0	0	0	0	0	18
2011	24	0	0	0	0	0	0,8
2011	24	0	0	0	0	0	3,9
2011	15	1	0	0	0	0	3,8
2011	15	0	2	0	0	1	0
2011	15	0	0	0	0	0	0
2011	15	0	0	0	0	0	0
2011	15	0	0	0	0	0	0
2011	15	0	0	0	0	0	0
2011	43	0	0	0	0	0	0
2011	43	0	1	1	0	0	0,9
2011	43	0	0	0	0	0	0
2011	43	0	0	0	0	0	0
2011	43	0	0	0	0	0	1,8
2011	43	0	0	0	0	0	0
2011	43	0	0	0	0	0	1,9
2011	43	0	0	0	0	0	0
2011	43	0	0	0	0	0	0,9
2011	43	0	0	0	0	0	0
2011	17	0	0	0	0	0	10,2
2011	17	0	0	0	0	0	17,7

2011	17	0	0	0	0	0	15,4
2011	17	0	0	0	0	0	11,1
2011	17	1	0	0	0	0	62,7
2011	17	0	0	0	0	0	22,7
2011	17	1	0	0	0	0	59,1
2011	17	0	0	0	0	0	2
2011	17	0	0	0	0	0	0,8
2011	17	1	0	0	0	0	23,4
2011	32	0	0	0	0	0	2,2
2011	32	0	0	0	0	0	1,6
2011	32	0	0	0	0	0	22,5
2011	32	0	0	0	0	0	4,6
2011	32	0	0	0	0	0	0
2011	32	0	0	0	0	0	7,6
2011	32	0	0	0	0	0	26,5
2011	32	0	0	0	0	0	13,8
2011	32	0	0	0	0	0	15,1
2011	32	0	0	0	0	0	6,1
2011	20	0	0	0	0	0	18,3
2011	20	0	0	0	0	0	12
2011	20	0	0	0	0	0	2,7
2011	20	0	0	0	0	0	11,7
2011	20	0	3	1	0	0	7,1
2011	20	0	0	0	0	0	12,1
2011	20	1	0	0	0	0	10,7
2011	20	1	0	0	0	0	5,1
2011	20	0	0	0	0	0	12,3
2011	20	0	0	0	0	0	3,8
2011	19	0	0	0	0	0	10,2
2011	19	0	0	0	0	0	9
2011	19	0	0	0	0	0	2,3
2011	19	0	0	0	0	0	3,5
2011	19	0	0	0	0	0	16,6
2011	19	0	0	0	0	0	7,3
2011	19	0	0	0	0	0	3,4
2011	19	0	0	0	0	0	8,2
2011	19	0	0	0	0	0	0
2011	19	0	0	0	0	0	7,6
2011	12	0	0	0	0	0	7,1
2011	12	1	0	0	0	0	4,8
2011	12	1	0	0	0	0	8,5
2011	12	1	0	0	0	0	6,8
2011	12	1	0	0	0	0	6,1
2011	12	0	0	0	0	0	1,7
2011	12	1	0	0	0	0	3,8
2011	12	0	0	0	0	0	9,4
2011	12	0	0	0	0	0	8
2011	12	0	0	0	0	0	3,4
2012	33	1	0	0	0	0	18,62
2012	33	0	0	0	0	0	1,96
2012	33	1	0	0	0	0	8,84
2012	33	0	1	0	1	0	12,93
2012	33	0	1	0	1	0	15,53

2012	33	0	0	0	0	0	17,54
2012	33	0	0	0	0	0	5,3
2012	33	0	0	0	0	0	27,19
2012	33	0	2	0	1	0	6,6
2012	33	0	0	0	0	0	13,44
2012	13	1	0	0	0	0	22,7
2012	13	1	0	0	0	0	10,4
2012	13	0	0	0	0	0	2,2
2012	13	0	2	1	0	0	4,7
2012	13	1	1	1	0	0	11,1
2012	13	1	0	0	0	0	7,9
2012	13	1	2	1	0	0	6,2
2012	13	1	0	0	0	0	4,7
2012	13	1	0	0	0	0	1,9
2012	13	1	0	0	0	0	11,3
2012	14	0	0	0	0	0	1,11
2012	14	0	0	0	0	0	0
2012	14	0	1	1	0	0	0
2012	14	0	0	0	0	0	2,02
2012	14	0	0	0	0	0	0,75
2012	14	0	0	0	0	0	0
2012	14	0	0	0	0	0	0
2012	14	0	0	0	0	0	1,45
2012	14	0	0	0	0	0	2,83
2012	14	0	0	0	0	0	0
2012	44	0	0	0	0	0	0,96
2012	44	0	0	0	0	0	5,45
2012	44	0	0	0	0	0	0
2012	44	1	0	0	0	0	1,62
2012	44	0	0	0	0	0	0
2012	44	0	0	0	0	0	2,7
2012	44	1	0	0	0	0	0
2012	44	0	0	0	0	0	1,61
2012	44	0	0	0	0	0	1,9
2012	44	1	0	0	0	0	0
2012	1	0	0	0	0	0	0
2012	1	0	0	0	0	0	0,9
2012	1	0	0	0	0	0	0
2012	1	0	0	0	0	0	3,2
2012	1	0	0	0	0	0	1
2012	1	0	0	0	0	0	0
2012	1	0	0	0	0	0	0,8
2012	1	0	0	0	0	0	2,8
2012	1	1	0	0	0	0	2,3
2012	1	0	0	0	0	0	12
2012	9	0	0	0	0	0	1,83
2012	9	1	0	0	0	0	8,77
2012	9	0	0	0	0	0	9,09
2012	9	0	0	0	0	0	1,12
2012	9	0	0	0	0	0	4,54
2012	9	1	2	0	1	0	0
2012	9	1	0	0	0	0	2,27
2012	9	0	0	0	0	0	1,9

2012	9	1	0	0	0	0	0,97
2012	9	0	0	0	0	0	4,54
2012	27	1	0	0	0	0	34,4
2012	27	1	0	0	0	0	63,6
2012	27	1	0	0	0	0	13,9
2012	27	1	0	0	0	0	20,2
2012	27	0	0	0	0	0	12,6
2012	27	1	0	0	0	0	34,7
2012	27	1	0	0	0	0	15,3
2012	27	1	0	0	0	0	23,8
2012	27	1	0	0	0	0	32,4
2012	27	1	0	0	0	0	31,6
2012	30	0	0	0	0	0	0,9
2012	30	1	0	0	0	0	2,77
2012	30	0	0	0	0	0	15,03
2012	30	0	0	0	0	0	4,46
2012	30	0	0	0	0	0	5,82
2012	30	1	0	0	0	0	6,14
2012	30	1	0	0	0	0	7,4
2012	30	0	0	0	0	0	4,44
2012	30	0	0	0	0	0	1,55
2012	30	0	0	0	0	0	3,88
2012	5	0	0	0	0	0	8,97
2012	5	0	0	0	0	0	7,54
2012	5	0	0	0	0	0	3,15
2012	5	0	0	0	0	0	0
2012	5	0	0	0	0	0	1,16
2012	5	0	0	0	0	0	16,48
2012	5	0	0	0	0	0	4,27
2012	5	1	0	0	0	0	6,61
2012	5	0	0	0	0	0	16,12
2012	5	1	0	0	0	0	13,28
2012	3	0	3	0	1	0	6,25
2012	3	1	0	0	0	0	13,26
2012	3	1	0	0	0	0	43,63
2012	3	1	0	0	0	0	55
2012	3	0	0	0	0	0	8,33
2012	3	0	0	0	0	0	11,65
2012	3	0	0	0	0	0	29,9
2012	3	1	0	0	0	0	16,26
2012	3	0	0	0	0	0	35,11
2012	3	0	0	0	0	0	14,14
2012	7	1	0	0	0	0	0,8
2012	7	0	0	0	0	0	6,97
2012	7	0	0	0	0	0	0
2012	7	0	0	0	0	0	0
2012	7	0	0	0	0	0	0
2012	7	0	2	0	1	0	0,78
2012	7	1	0	0	0	0	0
2012	7	0	0	0	0	0	18,29
2012	7	0	0	0	0	0	3,63
2012	7	0	0	0	0	0	1,03
2012	18	0	0	0	0	0	1,04

2012	18	0	0	0	0	0	9,41
2012	18	0	0	0	0	0	0
2012	18	0	0	0	0	0	2,97
2012	18	0	0	0	0	0	0
2012	18	0	0	0	0	0	0,88
2012	18	0	0	0	0	0	0
2012	18	0	0	0	0	0	1,94
2012	18	0	0	0	0	0	0
2012	18	0	0	0	0	0	0,81
2012	38	0	0	0	0	0	2,6
2012	38	0	0	0	0	0	7,3
2012	38	0	0	0	0	0	4,8
2012	38	1	0	0	0	0	10,7
2012	38	0	0	0	0	0	4,3
2012	38	0	0	0	0	0	32,1
2012	38	1	0	0	0	0	3,1
2012	38	0	0	0	0	0	50
2012	38	0	0	0	0	0	16
2012	38	0	0	0	0	0	5,1
2012	42	0	0	0	0	0	0
2012	42	1	0	0	0	0	3,6
2012	42	0	0	0	0	0	11,67
2012	42	0	0	0	0	0	0
2012	42	0	0	0	0	0	16,21
2012	42	0	0	0	0	0	0
2012	42	0	0	0	0	0	17,16
2012	42	0	3	1	0	0	4,62
2012	42	1	0	0	0	0	14,4
2012	42	1	0	0	0	0	8,41
2012	11	0	0	0	0	0	1,8
2012	11	0	0	0	0	0	3,77
2012	11	0	0	0	0	0	1,06
2012	11	0	0	0	0	0	1,94
2012	11	0	0	0	0	0	3,41
2012	11	0	0	0	0	0	0,89
2012	11	0	0	0	0	0	1,81
2012	11	0	0	0	0	0	13,72
2012	11	0	0	0	0	0	7,07
2012	11	0	0	0	0	0	4,5
2012	37	0	0	0	0	0	0
2012	37	0	0	0	0	0	0,94
2012	37	0	0	0	0	0	1,05
2012	37	0	0	0	0	0	0
2012	37	0	0	0	0	0	0
2012	37	0	0	0	0	0	0
2012	37	0	2	0	1	0	1,58
2012	37	0	0	0	0	0	2,97
2012	37	0	0	0	0	0	2,04
2012	37	0	0	0	0	0	0
2012	8	0	0	0	0	0	0
2012	8	0	0	0	0	0	0
2012	8	0	0	0	0	0	2
2012	8	0	0	0	0	0	2

2012	8	0	0	0	0	0	3,3
2012	8	0	0	0	0	0	4,1
2012	8	0	0	0	0	0	8,7
2012	8	0	0	0	0	0	9,2
2012	8	0	0	0	0	0	1,1
2012	8	0	0	0	0	0	4
2012	16	0	0	0	0	0	0
2012	16	0	0	0	0	0	0,9
2012	16	0	0	0	0	0	3,33
2012	16	0	0	0	0	0	0
2012	16	0	0	0	0	0	0
2012	16	0	0	0	0	0	7,4
2012	16	0	0	0	0	0	1,86
2012	16	0	0	0	0	0	0
2012	16	0	0	0	0	0	5,08
2012	16	0	0	0	0	0	1,16
2012	31	0	0	0	0	0	0,96
2012	31	0	0	0	0	0	0
2012	31	1	0	0	0	0	0
2012	31	0	1	0	1	0	0
2012	31	0	0	0	0	0	0
2012	31	0	0	0	0	0	0
2012	31	0	0	0	0	0	0
2012	31	0	1	0	1	0	0
2012	31	0	0	0	0	0	0
2012	31	0	0	0	0	0	0,9
2012	26	0	0	0	0	0	1
2012	26	0	0	0	0	0	0
2012	26	0	0	0	0	0	0
2012	26	0	0	0	0	0	1,5
2012	26	0	0	0	0	0	0,8
2012	26	0	0	0	0	0	2,1
2012	26	0	0	0	0	0	1,1
2012	26	0	0	0	0	0	0
2012	26	0	0	0	0	0	0,9
2012	26	0	0	0	0	0	0,9
2012	24	1	0	0	0	0	12,1
2012	24	0	0	0	0	0	15,9
2012	24	0	0	0	0	0	4,4
2012	24	1	0	0	0	0	3,9
2012	24	0	0	0	0	0	3,3
2012	24	0	0	0	0	0	3,7
2012	24	0	0	0	0	0	3,2
2012	24	0	0	0	0	0	7,1
2012	24	0	0	0	0	0	7,5
2012	24	0	0	0	0	0	1,9
2012	15	0	0	0	0	0	1,05
2012	15	0	0	0	0	0	0
2012	15	0	1	1	0	0	1,01
2012	15	0	0	0	0	0	1,11
2012	15	0	0	0	0	0	0
2012	15	0	0	0	0	0	7,55
2012	15	0	0	0	0	0	7,69

2012	15	0	0	0	0	0	4,76
2012	15	0	0	0	0	0	3,45
2012	15	0	0	0	0	0	7,14
2012	43	0	0	0	0	0	3,09
2012	43	0	0	0	0	0	0
2012	43	0	0	0	0	0	0,92
2012	43	0	0	0	0	0	3,61
2012	43	0	0	0	0	0	8,73
2012	43	0	0	0	0	0	1,75
2012	43	0	0	0	0	0	0,87
2012	43	0	0	0	0	0	9,47
2012	43	0	0	0	0	0	2,5
2012	43	0	0	0	0	0	5,31
2012	17	0	0	0	0	0	8,1
2012	17	0	0	0	0	0	3,7
2012	17	0	0	0	0	0	18,3
2012	17	1	0	0	0	0	15,1
2012	17	0	0	0	0	0	7,95
2012	17	0	0	0	0	0	0,92
2012	17	0	0	0	0	0	6,48
2012	17	0	0	0	0	0	10,87
2012	17	0	0	0	0	0	5,77
2012	17	0	0	0	0	0	0,86
2012	20	0	0	0	0	0	16,35
2012	20	0	0	0	0	0	1,04
2012	20	0	0	0	0	0	3,49
2012	20	0	0	0	0	0	0
2012	20	0	2	0	1	0	1,16
2012	20	0	0	0	0	0	0
2012	20	0	0	0	0	0	6,9
2012	20	0	0	0	0	0	32,61
2012	20	0	0	0	0	0	6,06
2012	20	0	0	0	0	0	13,86
2012	19	0	0	0	0	0	0
2012	19	0	0	0	0	0	3,29
2012	19	0	0	0	0	0	2,67
2012	19	1	0	0	0	0	0
2012	19	0	0	0	0	0	2,04
2012	19	0	0	0	0	0	0
2012	19	0	0	0	0	0	0
2012	19	0	0	0	0	0	0,92
2012	19	0	0	0	0	0	14,13
2012	19	0	0	0	0	0	0,96
2012	12	0	3	0	1	0	22,64
2012	12	0	1	0	1	0	15,68
2012	12	0	0	0	0	0	0
2012	12	0	1	0	1	0	21,42
2012	12	0	2	0	1	0	6,12
2012	12	0	3	0	1	0	0
2012	12	0	1	0	1	0	16,66
2012	12	0	1	0	1	0	26,19
2012	12	0	2	0	1	0	4
2012	12	0	3	0	1	0	0

2013	33	0	0	0	0	0	5,03
2013	33	0	0	0	0	0	2,27
2013	33	0	1	0	1	0	2,4
2013	33	0	1	0	0	1	4,24
2013	33	0	3	0	1	0	10,77
2013	33	0	1	1	0	0	1,74
2013	33	1	0	0	0	0	3,68
2013	33	0	0	0	0	0	8
2013	33	0	1	0	1	0	9,3
2013	33	0	0	0	0	0	11,64
2013	13	0	2	1	0	0	0
2013	13	0	0	0	0	0	1,06
2013	13	0	0	0	0	0	5,61
2013	13	0	2	0	1	0	0,81
2013	13	0	0	0	0	0	0
2013	13	0	0	0	0	0	4,13
2013	13	0	2	1	0	0	1,79
2013	13	0	0	0	0	0	0
2013	13	0	0	0	0	0	0,85
2013	13	0	0	0	0	0	0
2013	14	0	0	0	0	0	1
2013	14	0	0	0	0	0	1,83
2013	14	0	0	0	0	0	1,71
2013	14	0	0	0	0	0	4,72
2013	14	0	0	0	0	0	5,22
2013	14	0	0	0	0	0	4,84
2013	14	0	0	0	0	0	6,57
2013	14	0	0	0	0	0	2,17
2013	14	0	0	0	0	0	2,38
2013	14	0	0	0	0	0	5,51
2013	25	0	0	0	0	0	19,83
2013	25	0	0	0	0	0	2,72
2013	25	0	0	0	0	0	9,15
2013	25	0	0	0	0	0	7,33
2013	25	0	0	0	0	0	1,55
2013	25	0	0	0	0	0	10,81
2013	25	1	0	0	0	0	27,39
2013	25	1	0	0	0	0	13,99
2013	25	0	0	0	0	0	3,23
2013	25	1	0	0	0	0	3,01
2013	44	1	0	0	0	0	3,62
2013	44	0	0	0	0	0	77,91
2013	44	0	0	0	0	0	2,56
2013	44	0	0	0	0	0	5,98
2013	44	0	0	0	0	0	11,85
2013	44	1	1	0	1	0	0,75
2013	44	1	0	0	0	0	0,78
2013	44	0	0	0	0	0	0
2013	44	0	0	0	0	0	2,22
2013	44	0	0	0	0	0	1,15
2013	27	0	0	0	0	0	0,86
2013	27	0	0	0	0	0	0
2013	27	0	0	0	0	0	0

2013	27	0	0	0	0	0	0
2013	27	1	0	0	0	0	0,86
2013	27	0	0	0	0	0	0
2013	27	0	0	0	0	0	0
2013	27	0	0	0	0	0	0
2013	27	0	0	0	0	0	0
2013	30	0	0	0	0	0	3
2013	30	0	0	0	0	0	3,85
2013	30	0	1	0	1	0	4,38
2013	30	0	0	0	0	0	3,26
2013	30	0	0	0	0	0	3,23
2013	30	0	0	0	0	0	6,86
2013	30	0	0	0	0	0	8,26
2013	30	0	0	0	0	0	4,1
2013	30	0	0	0	0	0	4,65
2013	30	0	0	0	0	0	1,4
2013	3	0	0	0	0	0	1,79
2013	3	0	0	0	0	0	8,65
2013	3	0	0	0	0	0	1,08
2013	3	0	0	0	0	0	2,4
2013	3	0	0	0	0	0	22,72
2013	3	0	0	0	0	0	0,95
2013	3	0	2	0	1	0	2,78
2013	3	0	1	0	1	0	2,38
2013	3	0	0	0	0	0	0,78
2013	3	0	0	0	0	0	1,16
2013	28	0	0	0	0	0	0
2013	28	0	3	0	1	0	0,79
2013	28	0	3	0	0	1	0
2013	28	0	0	0	0	0	0
2013	28	0	0	0	0	0	2,31
2013	28	0	1	1	0	0	0
2013	28	1	0	0	0	0	0
2013	28	0	1	1	0	0	0
2013	28	0	0	0	0	0	0,97
2013	28	0	0	0	0	0	1,69
2013	7	0	0	0	0	0	0
2013	7	0	0	0	0	0	0
2013	7	0	0	0	0	0	0
2013	7	0	1	0	1	0	0
2013	7	0	0	0	0	0	2,11
2013	7	1	1	0	1	0	0,99
2013	7	0	1	0	1	0	0
2013	7	1	0	0	0	0	0
2013	7	0	0	0	0	0	0,88
2013	7	0	0	0	0	0	0
2013	18	0	0	0	0	0	4,12
2013	18	0	1	1	0	0	6,09
2013	18	0	0	0	0	0	4,79
2013	18	0	0	0	0	0	0
2013	18	0	0	0	0	0	5,83
2013	18	0	0	0	0	0	5,67
2013	18	0	0	0	0	0	5,3

2013	18	0	1	0	0	1	0,86
2013	18	0	1	1	0	0	0
2013	18	0	0	0	0	0	14,29
2013	40	0	0	0	0	0	3,28
2013	40	0	1	1	0	0	8,4
2013	40	0	0	0	0	0	21,43
2013	40	1	0	0	0	0	28,44
2013	40	1	0	0	0	0	0
2013	40	0	0	0	0	0	7,25
2013	40	1	0	0	0	0	2,75
2013	40	0	1	1	0	0	7,08
2013	42	0	0	0	0	0	6,25
2013	42	0	0	0	0	0	3,5
2013	42	0	0	0	0	0	1,03
2013	42	0	0	0	0	0	1,65
2013	42	0	1	0	1	0	2,34
2013	42	0	0	0	0	0	0
2013	42	0	2	0	1	0	0
2013	42	0	0	0	0	0	2,34
2013	42	0	0	0	0	0	0
2013	42	0	0	0	0	0	1,65
2013	11	0	0	0	0	0	0,96
2013	11	0	0	0	0	0	0
2013	11	0	0	0	0	0	1,69
2013	11	0	0	0	0	0	0
2013	11	0	0	0	0	0	0,88
2013	11	0	0	0	0	0	0
2013	11	0	0	0	0	0	0,97
2013	11	0	0	0	0	0	0
2013	11	1	0	0	0	0	1,87
2013	11	0	0	0	0	0	0,87
2013	8	0	0	0	0	0	0
2013	8	0	0	0	0	0	0
2013	8	0	0	0	0	0	0,82
2013	8	0	0	0	0	0	0
2013	8	0	0	0	0	0	0
2013	8	0	0	0	0	0	0
2013	8	0	0	0	0	0	0
2013	8	0	0	0	0	0	0
2013	8	0	0	0	0	0	0
2013	8	0	0	0	0	0	0
2013	31	0	0	0	0	0	0
2013	31	0	0	0	0	0	0
2013	31	0	0	0	0	0	8,4
2013	31	0	0	0	0	0	2,48
2013	31	0	0	0	0	0	0
2013	31	0	0	0	0	0	0
2013	31	0	0	0	0	0	0
2013	31	0	0	0	0	0	0
2013	31	0	0	0	0	0	0
2013	31	0	0	0	0	0	0
2013	26	0	1	0	0	1	0
2013	26	0	0	0	0	0	1,74

2013	26	1	1	1	0	0	8
2013	26	0	1	1	0	0	6,38
2013	26	0	0	0	0	0	0
2013	26	0	0	0	0	0	0
2013	26	0	0	0	0	0	4,27
2013	26	0	0	0	0	0	0,82
2013	26	0	1	1	0	0	0
2013	26	0	0	0	0	0	0
2013	24	1	2	0	1	0	15,63
2013	24	1	0	0	0	0	30,47
2013	24	0	0	0	0	0	24,44
2013	24	0	0	0	0	0	22,22
2013	24	0	0	0	0	0	12,1
2013	24	0	0	0	0	0	11
2013	24	1	2	0	1	0	5,62
2013	24	0	3	0	1	0	12,05
2013	24	1	1	0	1	0	54,17
2013	24	0	0	0	0	0	10,1
2013	21	0	0	0	0	0	0
2013	21	0	0	0	0	0	0
2013	21	0	1	1	0	0	0
2013	21	0	0	0	0	0	0
2013	21	0	3	1	0	0	2,88
2013	21	0	2	1	0	0	1
2013	21	0	0	0	0	0	0
2013	21	1	0	0	0	0	0
2013	21	0	0	0	0	0	0
2013	15	1	0	0	0	0	3,92
2013	15	0	0	0	0	0	2,04
2013	15	0	0	0	0	0	6,73
2013	15	0	0	0	0	0	6,02
2013	15	0	0	0	0	0	10,64
2013	15	0	0	0	0	0	6,78
2013	15	0	0	0	0	0	9,71
2013	15	1	0	0	0	0	5,31
2013	15	0	0	0	0	0	3,85
2013	15	0	0	0	0	0	1,89
2013	43	0	0	0	0	0	0,89
2013	43	0	0	0	0	0	0,97
2013	43	0	0	0	0	0	0
2013	43	0	0	0	0	0	3,13
2013	43	0	0	0	0	0	2,44
2013	43	0	0	0	0	0	1,1
2013	43	0	0	0	0	0	0
2013	43	0	0	0	0	0	0
2013	43	0	2	1	0	0	0
2013	43	0	0	0	0	0	0
2013	17	0	0	0	0	0	4,95
2013	17	0	0	0	0	0	3,74
2013	17	0	0	0	0	0	2,83
2013	17	0	0	0	0	0	27,37
2013	17	0	0	0	0	0	1,56
2013	17	0	0	0	0	0	2,65

2013	17	0	0	0	0	0	0,78
2013	17	0	0	0	0	0	3,15
2013	17	0	1	1	0	0	29,85
2013	17	1	0	0	0	0	15,2
2013	19	0	0	0	0	0	0
2013	19	0	0	0	0	0	0
2013	19	0	0	0	0	0	0
2013	19	0	0	0	0	0	0
2013	19	0	0	0	0	0	0
2013	19	0	0	0	0	0	0
2013	19	0	0	0	0	0	0
2013	19	0	0	0	0	0	0
2013	19	0	0	0	0	0	0
2013	19	0	0	0	0	0	0
2014	13	0	0	0	0	0	0
2014	13	0	0	0	0	0	1,28
2014	13	0	0	0	0	0	2,15
2014	13	0	1	1	0	0	5,22
2014	13	0	0	0	0	0	1,14
2014	13	0	0	0	0	0	1,32
2014	13	0	0	0	0	0	6
2014	13	0	0	0	0	0	1,18
2014	13	0	0	0	0	0	5,6
2014	13	0	0	0	0	0	3,66
2014	14	1	0	0	0	0	4,1
2014	14	0	0	0	0	0	2,48
2014	14	0	0	0	0	0	0,81
2014	14	1	0	0	0	0	33,93
2014	14	1	0	0	0	0	7,22
2014	14	0	0	0	0	0	3,64
2014	14	1	0	0	0	0	0
2014	14	0	0	0	0	0	2,77
2014	14	0	0	0	0	0	1,8
2014	14	0	0	0	0	0	0
2014	25	1	0	0	0	0	3,94
2014	25	0	0	0	0	0	0
2014	25	0	0	0	0	0	3,2
2014	25	1	0	0	0	0	4,35
2014	25	1	0	0	0	0	3,54
2014	25	0	0	0	0	0	5,69
2014	25	1	0	0	0	0	18,85
2014	25	0	0	0	0	0	4,93
2014	25	0	0	0	0	0	6,8
2014	25	0	0	0	0	0	0
2014	46	0	0	0	0	0	10,09
2014	46	0	0	0	0	0	3,45
2014	46	0	0	0	0	0	3,54
2014	46	1	0	0	0	0	7,79
2014	46	0	0	0	0	0	5,88
2014	46	0	0	0	0	0	0
2014	46	0	0	0	0	0	8,13
2014	46	0	0	0	0	0	1,47
2014	46	1	0	0	0	0	0

2014	46	0	0	0	0	0	5,36
2014	44	0	0	0	0	0	0
2014	44	0	0	0	0	0	1,49
2014	44	0	0	0	0	0	0
2014	44	0	0	0	0	0	0
2014	44	0	0	0	0	0	2,42
2014	44	0	0	0	0	0	3,33
2014	44	0	0	0	0	0	0
2014	44	0	0	0	0	0	0
2014	44	0	0	0	0	0	0
2014	44	0	0	0	0	0	0
2014	27	0	0	0	0	0	0
2014	27	1	0	0	0	0	3,78
2014	27	0	0	0	0	0	1,18
2014	27	0	0	0	0	0	2,7
2014	27	0	0	0	0	0	1,37
2014	27	1	0	0	0	0	6,58
2014	27	0	0	0	0	0	1,85
2014	27	1	0	0	0	0	2,3
2014	27	0	0	0	0	0	4,14
2014	27	1	0	0	0	0	7,53
2014	47	0	0	0	0	0	32,58
2014	47	1	0	0	0	0	13,33
2014	47	1	0	0	0	0	4,35
2014	47	0	0	0	0	0	1,16
2014	47	0	0	0	0	0	1,3
2014	47	0	0	0	0	0	9,1
2014	47	0	0	0	0	0	2
2014	47	0	1	0	1	0	15,04
2014	47	0	0	0	0	0	13,7
2014	47	0	0	0	0	0	2,75
2014	30	1	0	0	0	0	10
2014	30	0	0	0	0	0	2,4
2014	30	1	0	0	0	0	15,04
2014	30	0	0	0	0	0	3,9
2014	30	0	0	0	0	0	25,86
2014	30	0	0	0	0	0	14,78
2014	30	0	2	0	1	0	7,6
2014	30	1	0	0	0	0	3,1
2014	30	0	0	0	0	0	4,03
2014	30	0	0	0	0	0	2,1
2014	3	0	1	0	1	0	2,61
2014	3	0	0	0	0	0	7,63
2014	3	0	2	0	1	0	4,9
2014	3	0	0	0	0	0	0,92
2014	3	0	0	0	0	0	0
2014	3	1	0	0	0	0	41,46
2014	3	0	0	0	0	0	5,75
2014	3	0	0	0	0	0	1,9
2014	3	0	0	0	0	0	1,04
2014	3	0	0	0	0	0	1,27
2014	48	0	0	0	0	0	8,4
2014	48	0	0	0	0	0	8,11

2014	48	0	0	0	0	0	3,88
2014	48	0	0	0	0	0	6,41
2014	48	1	0	0	0	0	1,05
2014	48	0	0	0	0	0	6,73
2014	48	0	0	0	0	0	7,76
2014	48	0	0	0	0	0	9,7
2014	48	1	0	0	0	0	5,05
2014	48	0	0	0	0	0	20,6
2014	28	0	0	0	0	0	2,38
2014	28	0	0	0	0	0	0
2014	28	0	0	0	0	0	0
2014	28	1	0	0	0	0	0
2014	28	0	0	0	0	0	0
2014	28	0	0	0	0	0	0
2014	28	0	0	0	0	0	0,98
2014	28	0	0	0	0	0	2,48
2014	28	0	0	0	0	0	0
2014	28	0	0	0	0	0	0,85
2014	18	0	0	0	0	0	1,01
2014	18	0	0	0	0	0	0
2014	18	1	0	0	0	0	8,23
2014	18	0	0	0	0	0	1,4
2014	18	0	0	0	0	0	2,2
2014	18	0	0	0	0	0	1,34
2014	18	0	0	0	0	0	2,1
2014	18	0	0	0	0	0	0
2014	18	0	0	0	0	0	0
2014	18	0	0	0	0	0	4
2014	26	1	0	0	0	0	20,91
2014	26	1	0	0	0	0	139,22
2014	26	1	0	0	0	0	133,33
2014	26	1	0	0	0	0	66,13
2014	26	1	0	0	0	0	49,64
2014	26	0	0	0	0	0	41,11
2014	42	0	0	0	0	0	0,97
2014	42	0	0	0	0	0	0,93
2014	42	0	0	0	0	0	0
2014	42	0	0	0	0	0	0
2014	42	0	0	0	0	0	0
2014	42	0	0	0	0	0	0
2014	42	0	0	0	0	0	0
2014	42	0	0	0	0	0	0
2014	42	0	0	0	0	0	0
2014	42	0	0	0	0	0	0
2014	11	0	0	0	0	0	20,24
2014	11	1	0	0	0	0	4,76
2014	11	1	0	0	0	0	39,77
2014	11	1	1	0	1	0	11,38
2014	11	1	0	0	0	0	11,58
2014	11	1	0	0	0	0	13,44
2014	11	1	0	0	0	0	8,33
2014	11	1	0	0	0	0	2,54
2014	11	1	0	0	0	0	16,26

2014	11	1	0	0	0	0	13,88
2014	8	0	0	0	0	0	0
2014	8	0	0	0	0	0	0
2014	8	0	0	0	0	0	1,07
2014	8	0	0	0	0	0	0
2014	8	0	0	0	0	0	0
2014	8	0	0	0	0	0	0
2014	8	0	0	0	0	0	0,9
2014	8	0	0	0	0	0	0
2014	8	0	0	0	0	0	0,84
2014	8	0	0	0	0	0	0
2014	26	0	0	0	0	0	8,53
2014	26	1	0	0	0	0	2,73
2014	26	0	0	0	0	0	4,17
2014	26	0	0	0	0	0	0
2014	26	0	0	0	0	0	10,08
2014	26	0	0	0	0	0	0
2014	26	0	0	0	0	0	1,01
2014	26	0	0	0	0	0	0
2014	26	1	0	0	0	0	1,06
2014	26	0	0	0	0	0	1,22
2014	24	1	0	0	0	0	6,37
2014	24	1	0	0	0	0	40,27
2014	24	1	1	0	1	0	4,65
2014	24	1	3	0	1	0	11,46
2014	24	1	0	0	0	0	31,2
2014	24	1	0	0	0	0	18,6
2014	21	0	0	0	0	0	0
2014	21	1	0	0	0	0	0
2014	21	0	0	0	0	0	0,81
2014	21	0	0	0	0	0	0,97
2014	21	0	0	0	0	0	0
2014	21	0	0	0	0	0	0
2014	21	0	0	0	0	0	0
2014	21	0	0	0	0	0	0
2014	21	0	0	0	0	0	0
2014	21	0	0	0	0	0	0
2014	15	0	0	0	0	0	19,19
2014	15	0	0	0	0	0	11,2
2014	15	0	0	0	0	0	4,51
2014	15	0	2	1	0	0	0
2014	15	0	0	0	0	0	0
2014	15	0	1	1	0	0	14
2014	15	0	0	0	0	0	2,4
2014	15	0	0	0	0	0	3,88
2014	15	0	0	0	0	0	1,8
2014	15	1	0	0	0	0	4,29
2014	43	0	0	0	0	0	0
2014	43	0	3	0	1	0	0
2014	43	0	3	1	0	0	0
2014	43	0	0	0	0	0	1,1
2014	43	0	0	0	0	0	4
2014	43	0	0	0	0	0	0

2014	43	0	0	0	0	0	0,76
2014	43	0	0	0	0	0	1,07
2014	43	0	0	0	0	0	3,06
2014	43	0	0	0	0	0	1,72
2014	16	0	0	0	0	0	0,83
2014	16	1	0	0	0	0	0
2014	16	0	0	0	0	0	6,06
2014	16	0	0	0	0	0	0
2014	16	0	0	0	0	0	0,89
2014	16	1	0	0	0	0	2,94
2014	16	0	0	0	0	0	1,63
2014	16	0	0	0	0	0	0,97
2014	16	0	0	0	0	0	4,8
2014	16	0	0	0	0	0	7,28
2014	19	0	0	0	0	0	2,08
2014	19	0	0	0	0	0	2,3
2014	19	0	0	0	0	0	1,02
2014	19	0	0	0	0	0	0
2014	19	0	0	0	0	0	3,96
2014	19	0	0	0	0	0	0
2014	19	0	0	0	0	0	0
2014	19	0	0	0	0	0	0
2014	19	0	0	0	0	0	1,18
2014	19	0	0	0	0	0	1,04
2015	33	0	0	0	0	0	15,1
2015	33	1	0	0	0	0	10,84
2015	33	0	0	0	0	0	3,2
2015	33	0	0	0	0	0	1,9
2015	33	0	0	0	0	0	4,1
2015	33	0	0	0	0	0	6,6
2015	33	0	0	0	0	0	1,8
2015	33	0	0	0	0	0	1,9
2015	33	0	0	0	0	0	12
2015	33	0	0	0	0	0	13,4
2015	13	1	0	0	0	0	0
2015	13	0	0	0	0	0	0
2015	13	1	0	0	0	0	0
2015	13	1	0	0	0	0	0
2015	13	1	0	0	0	0	0
2015	13	1	0	0	0	0	0,8
2015	13	1	0	0	0	0	0
2015	13	0	0	0	0	0	0
2015	13	0	0	0	0	0	0
2015	13	1	0	0	0	0	2,05
2015	14	0	0	0	0	0	0
2015	14	0	0	0	0	0	2,6
2015	14	0	0	0	0	0	0
2015	14	0	0	0	0	0	0
2015	14	0	0	0	0	0	0
2015	14	0	0	0	0	0	2,3
2015	14	0	0	0	0	0	0
2015	14	0	0	0	0	0	0
2015	14	0	0	0	0	0	0

2015	14	0	0	0	0	0	0
2015	46	0	0	0	0	0	2
2015	46	0	0	0	0	0	12,8
2015	46	0	0	0	0	0	14,1
2015	46	0	0	0	0	0	12,3
2015	46	0	0	0	0	0	45,2
2015	46	1	0	0	0	0	1,44
2015	46	1	0	0	0	0	9,9
2015	46	0	0	0	0	0	20,8
2015	46	0	0	0	0	0	8,6
2015	46	1	0	0	0	0	8,97
2015	44	0	0	0	0	0	0
2015	44	0	0	0	0	0	0
2015	44	0	0	0	0	0	0
2015	44	0	0	0	0	0	0
2015	44	0	0	0	0	0	0
2015	44	0	0	0	0	0	0
2015	44	0	0	0	0	0	0
2015	44	0	0	0	0	0	1,4
2015	44	0	0	0	0	0	0
2015	44	1	0	0	0	0	1,4
2015	51	0	0	0	0	0	0
2015	51	0	0	0	0	0	0
2015	51	0	0	0	0	0	1,7
2015	51	0	0	0	0	0	0,8
2015	51	0	0	0	0	0	0
2015	51	0	0	0	0	0	0
2015	51	0	0	0	0	0	0
2015	51	0	0	0	0	0	0
2015	51	0	0	0	0	0	0
2015	51	0	0	0	0	0	0
2015	27	0	0	0	0	0	0
2015	27	0	0	0	0	0	0
2015	27	0	0	0	0	0	0
2015	27	0	0	0	0	0	0
2015	27	1	0	0	0	0	0
2015	27	0	0	0	0	0	0
2015	27	0	0	0	0	0	0
2015	27	0	0	0	0	0	0
2015	27	0	0	0	0	0	0
2015	27	0	0	0	0	0	0
2015	47	0	0	0	0	0	0
2015	47	0	0	0	0	0	0,7
2015	47	0	0	0	0	0	0
2015	47	0	0	0	0	0	0
2015	47	0	0	0	0	0	0,9
2015	47	0	0	0	0	0	0
2015	47	0	0	0	0	0	2,3
2015	47	0	0	0	0	0	0,8
2015	47	0	0	0	0	0	0
2015	47	0	0	0	0	0	0,6
2015	30	0	0	0	0	0	0,9
2015	30	0	0	0	0	0	1,8

2015	30	0	0	0	0	0	0,8
2015	30	0	0	0	0	0	2
2015	30	0	0	0	0	0	3,3
2015	30	0	0	0	0	0	1,9
2015	30	0	0	0	0	0	0
2015	30	0	0	0	0	0	2,6
2015	30	0	0	0	0	0	0,9
2015	30	0	0	0	0	0	1,5
2015	3	0	0	0	0	0	8,3
2015	3	0	0	0	0	0	16,5
2015	3	0	0	0	0	0	0,8
2015	3	0	0	0	0	0	8,1
2015	3	0	0	0	0	0	9,3
2015	3	0	0	0	0	0	1,5
2015	3	1	0	0	0	0	0,8
2015	3	0	0	0	0	0	7
2015	3	0	0	0	0	0	0,8
2015	3	0	0	0	0	0	3,3
2015	48	0	0	0	0	0	0
2015	48	0	0	0	0	0	0
2015	48	0	0	0	0	0	0
2015	48	0	0	0	0	0	0
2015	48	0	0	0	0	0	4,3
2015	48	0	1	1	0	0	1,8
2015	48	0	0	0	0	0	0
2015	48	0	0	0	0	0	0
2015	48	0	0	0	0	0	0,8
2015	48	1	2	0	1	0	0
2015	28	0	0	0	0	0	5,1
2015	28	0	0	0	0	0	14,5
2015	28	0	0	0	0	0	1,4
2015	28	0	0	0	0	0	3
2015	28	0	0	0	0	0	1,9
2015	28	0	0	0	0	0	0
2015	28	0	0	0	0	0	0,6
2015	28	0	0	0	0	0	8,3
2015	28	0	0	0	0	0	0
2015	28	0	0	0	0	0	3,6
2015	52	0	0	0	0	0	0
2015	52	0	0	0	0	0	0
2015	52	0	0	0	0	0	0
2015	52	0	0	0	0	0	0
2015	52	0	0	0	0	0	0
2015	52	0	0	0	0	0	0
2015	52	0	0	0	0	0	0
2015	52	0	0	0	0	0	0
2015	52	0	0	0	0	0	0
2015	52	0	0	0	0	0	0
2015	18	0	0	0	0	0	0
2015	18	0	0	0	0	0	0
2015	18	0	0	0	0	0	0
2015	18	0	0	0	0	0	0
2015	18	0	0	0	0	0	0

2015	18	0	0	0	0	0	0
2015	18	0	0	0	0	0	0
2015	18	0	0	0	0	0	1,5
2015	18	0	0	0	0	0	0
2015	18	0	0	0	0	0	0
2015	53	0	0	0	0	0	0
2015	53	0	0	0	0	0	0
2015	53	0	0	0	0	0	0,7
2015	53	1	0	0	0	0	1,15
2015	53	0	0	0	0	0	5,1
2015	53	0	0	0	0	0	0
2015	53	0	0	0	0	0	2,5
2015	53	0	0	0	0	0	0
2015	53	0	0	0	0	0	0,8
2015	53	0	0	0	0	0	0
2015	42	0	0	0	0	0	0
2015	42	0	0	0	0	0	0
2015	42	0	0	0	0	0	0
2015	42	0	0	0	0	0	0
2015	42	0	0	0	0	0	0
2015	42	0	0	0	0	0	0
2015	42	0	1	0	1	0	0
2015	42	0	0	0	0	0	0
2015	42	0	0	0	0	0	0
2015	42	0	0	0	0	0	0
2015	8	0	0	0	0	0	1,4
2015	8	0	0	0	0	0	0
2015	8	0	0	0	0	0	0
2015	8	0	0	0	0	0	2
2015	8	0	0	0	0	0	0
2015	8	0	0	0	0	0	0
2015	8	0	0	0	0	0	0,7
2015	8	0	0	0	0	0	0
2015	8	0	0	0	0	0	0
2015	8	0	0	0	0	0	0
2015	26	0	0	0	0	0	10,1
2015	26	0	0	0	0	0	0
2015	26	0	0	0	0	0	0
2015	26	0	0	0	0	0	0
2015	26	0	0	0	0	0	2,1
2015	26	0	0	0	0	0	4,8
2015	26	0	0	0	0	0	3,8
2015	21	0	3	1	0	0	25,8
2015	21	0	2	1	0	0	0
2015	21	0	0	0	0	0	1,2
2015	21	0	0	0	0	0	0
2015	21	0	0	0	0	0	0
2015	21	0	2	1	0	0	0
2015	21	0	0	0	0	0	0
2015	21	0	0	0	0	0	0
2015	21	0	1	1	0	0	0
2015	21	0	0	0	0	0	0
2015	15	0	0	0	0	0	10

2015	15	0	0	0	0	0	5,8
2015	15	0	0	0	0	0	2,7
2015	15	0	0	0	0	0	0
2015	15	0	0	0	0	0	0
2015	15	1	0	0	0	0	1
2015	15	0	0	0	0	0	0
2015	15	0	0	0	0	0	0,7
2015	15	0	0	0	0	0	0,7
2015	15	0	0	0	0	0	0,7
2015	43	0	0	0	0	0	0,7
2015	43	0	0	0	0	0	0
2015	43	0	0	0	0	0	0
2015	43	0	0	0	0	0	0
2015	43	0	0	0	0	0	3,2
2015	43	0	0	0	0	0	3,9
2015	43	0	0	0	0	0	0
2015	43	0	0	0	0	0	0,9
2015	43	0	0	0	0	0	2,9
2015	43	0	0	0	0	0	5,8
2015	17	0	0	0	0	0	1,6
2015	17	0	0	0	0	0	8,8
2015	17	0	0	0	0	0	5,3
2015	17	0	0	0	0	0	1,6
2015	17	0	0	0	0	0	1,8
2015	17	0	0	0	0	0	0
2015	17	0	0	0	0	0	0
2015	17	0	0	0	0	0	8,8
2015	17	0	0	0	0	0	0
2015	17	0	0	0	0	0	2
2015	19	0	0	0	0	0	0
2015	19	0	0	0	0	0	0
2015	19	0	1	0	1	0	0
2015	19	0	0	0	0	0	0
2015	19	0	0	0	0	0	0
2015	19	0	0	0	0	0	0
2015	19	0	0	0	0	0	0
2015	19	0	0	0	0	0	0
2015	19	0	0	0	0	0	0
2015	19	0	0	0	0	0	0
2016	33	1	0	0	0	0	10,8
2016	33	1	0	0	0	0	2,6
2016	33	1	0	0	0	0	0,6
2016	33	1	0	0	0	0	12,1
2016	33	1	0	0	0	0	5,4
2016	33	1	2	0	1	0	9,3
2016	33	1	0	0	0	0	11,5
2016	33	1	0	0	0	0	27,7
2016	33	1	0	0	0	0	9,9
2016	33	1	3	0	1	0	5,3
2016	49	1	0	0	0	0	13,2
2016	49	0	0	0	0	0	4,4
2016	49	1	0	0	0	0	48,5
2016	49	0	0	0	0	0	1,8

2016	49	1	0	0	0	0	12,7
2016	49	0	0	0	0	0	0,7
2016	49	1	0	0	0	0	22,4
2016	49	1	0	0	0	0	15,3
2016	49	1	0	0	0	0	1
2016	49	0	0	0	0	0	14,9
2016	13	0	0	0	0	0	0
2016	13	0	0	0	0	0	0
2016	13	0	3	1	0	0	0
2016	13	0	3	1	0	0	0
2016	13	1	0	0	0	0	0
2016	13	1	3	1	0	0	0
2016	13	0	0	0	0	0	0
2016	13	0	0	0	0	0	0,7
2016	13	0	0	0	0	0	0
2016	13	0	3	1	0	0	0
2016	14	1	0	0	0	0	5,7
2016	14	1	0	0	0	0	12,9
2016	14	0	0	0	0	0	1,3
2016	14	1	0	0	0	0	18,2
2016	14	0	0	0	0	0	15,3
2016	14	0	0	0	0	0	1,9
2016	14	0	0	0	0	0	7,4
2016	14	1	0	0	0	0	15,3
2016	14	1	0	0	0	0	24,7
2016	14	0	0	0	0	0	4,8
2016	55	0	0	0	0	0	5,3
2016	55	0	0	0	0	0	1,5
2016	55	0	0	0	0	0	3,2
2016	55	0	0	0	0	0	4,6
2016	55	1	0	0	0	0	8,7
2016	55	0	0	0	0	0	0
2016	55	0	0	0	0	0	1,9
2016	55	0	0	0	0	0	7,6
2016	55	0	0	0	0	0	0
2016	55	0	0	0	0	0	1,8
2016	56	0	0	0	0	0	0
2016	56	0	0	0	0	0	1,1
2016	56	0	0	0	0	0	0
2016	56	0	0	0	0	0	1,7
2016	56	0	0	0	0	0	0,6
2016	56	0	0	0	0	0	1,2
2016	56	0	0	0	0	0	0
2016	56	0	0	0	0	0	0,7
2016	56	0	0	0	0	0	1
2016	56	0	0	0	0	0	0,6
2016	46	0	0	0	0	0	4,5
2016	46	0	0	0	0	0	4,4
2016	46	1	0	0	0	0	0,9
2016	46	0	0	0	0	0	20,5
2016	46	1	0	0	0	0	8,5
2016	46	1	0	0	0	0	9,5
2016	46	0	0	0	0	0	0

2016	46	0	0	0	0	0	9,1
2016	46	0	0	0	0	0	6,6
2016	46	1	0	0	0	0	35,2
2016	44	0	0	0	0	0	5,3
2016	44	0	0	0	0	0	0,9
2016	44	1	2	0	1	0	0
2016	44	0	2	0	1	0	4,4
2016	44	0	0	0	0	0	1,7
2016	44	0	0	0	0	0	3,3
2016	44	0	3	0	1	0	0
2016	44	1	0	0	0	0	0,7
2016	44	1	1	0	1	0	0
2016	44	0	0	0	0	0	0
2016	51	1	3	0	1	0	3,5
2016	51	1	0	0	0	0	0,7
2016	51	0	2	0	1	0	0
2016	51	1	0	0	0	0	0
2016	51	1	0	0	0	0	8
2016	51	1	0	0	0	0	1,6
2016	51	1	0	0	0	0	0
2016	51	1	0	0	0	0	0,7
2016	51	1	0	0	0	0	44,7
2016	51	0	0	0	0	0	2,4
2016	27	0	0	0	0	0	0
2016	27	0	0	0	0	0	2,5
2016	27	0	0	0	0	0	1
2016	27	1	0	0	0	0	2,6
2016	27	0	0	0	0	0	1,6
2016	27	0	0	0	0	0	1,6
2016	27	0	0	0	0	0	0,8
2016	27	0	0	0	0	0	1
2016	27	0	0	0	0	0	1,7
2016	27	0	0	0	0	0	0
2016	47	0	1	1	0	0	0,9
2016	47	1	0	0	0	0	1,1
2016	47	0	0	0	0	0	4,9
2016	47	0	0	0	0	0	7,4
2016	47	0	0	0	0	0	4,1
2016	47	0	2	1	0	0	0,9
2016	47	0	0	0	0	0	13,7
2016	47	0	0	0	0	0	17,7
2016	47	0	0	0	0	0	29
2016	47	0	0	0	0	0	1,9
2016	30	0	0	0	0	0	0,7
2016	30	0	0	0	0	0	0
2016	30	0	0	0	0	0	0
2016	30	0	0	0	0	0	0,8
2016	30	0	0	0	0	0	1,3
2016	30	0	0	0	0	0	0
2016	30	0	0	0	0	0	0,8
2016	30	0	0	0	0	0	5,3
2016	30	0	0	0	0	0	5,9
2016	30	0	0	0	0	0	0,8

2016	3	0	0	0	0	0	20,4
2016	3	0	0	0	0	0	2,9
2016	3	0	0	0	0	0	8,9
2016	3	0	0	0	0	0	16,4
2016	3	0	0	0	0	0	8,6
2016	3	0	0	0	0	0	1,1
2016	3	0	0	0	0	0	2,8
2016	3	0	0	0	0	0	2,8
2016	3	0	2	0	1	0	4,2
2016	3	0	0	0	0	0	4
2016	57	1	0	0	0	0	0,6
2016	57	0	0	0	0	0	0
2016	57	0	0	0	0	0	2,4
2016	57	1	0	0	0	0	0
2016	57	0	0	0	0	0	2
2016	57	0	0	0	0	0	0
2016	57	1	0	0	0	0	0,7
2016	57	1	0	0	0	0	1,1
2016	57	0	0	0	0	0	0,7
2016	57	1	0	0	0	0	22,4
2016	28	1	3	0	1	0	23,2
2016	28	1	2	0	1	0	28,1
2016	28	0	0	0	0	0	6,4
2016	28	1	2	0	1	0	15,6
2016	28	1	2	0	1	0	13
2016	28	1	2	0	1	0	36,9
2016	28	1	3	0	1	0	26,5
2016	28	1	0	0	0	0	35,6
2016	28	1	2	0	1	0	44,2
2016	28	1	0	0	0	0	5,6
2016	58	0	1	0	1	0	6,7
2016	58	0	0	0	0	0	7,1
2016	58	0	0	0	0	0	7,3
2016	58	0	0	0	0	0	2,8
2016	58	0	2	0	1	0	0,6
2016	58	0	0	0	0	0	0
2016	58	0	0	0	0	0	1,9
2016	58	0	0	0	0	0	0
2016	58	1	0	0	0	0	16,7
2016	58	0	2	0	1	0	3,4
2016	52	0	0	0	0	0	13,2
2016	52	0	0	0	0	0	4,6
2016	52	0	0	0	0	0	7,5
2016	52	0	0	0	0	0	4,5
2016	52	0	0	0	0	0	18,5
2016	52	0	0	0	0	0	2,5
2016	52	0	0	0	0	0	3
2016	52	0	0	0	0	0	7,9
2016	52	0	0	0	0	0	3
2016	52	0	0	0	0	0	1,2
2016	42	0	1	0	1	0	0
2016	42	1	0	0	0	0	1,6
2016	42	1	0	0	0	0	3,1

2016	42	0	0	0	0	0	0
2016	42	0	0	0	0	0	2
2016	42	0	0	0	0	0	0
2016	42	1	1	0	1	0	0
2016	42	1	0	0	0	0	2,5
2016	42	0	1	0	1	0	0
2016	42	0	0	0	0	0	0
2016	8	0	0	0	0	0	0
2016	8	1	0	0	0	0	0,9
2016	8	0	0	0	0	0	0,9
2016	8	0	0	0	0	0	2,4
2016	8	0	0	0	0	0	0
2016	8	0	0	0	0	0	1,3
2016	8	0	0	0	0	0	0
2016	8	0	0	0	0	0	0
2016	8	0	0	0	0	0	0
2016	8	0	0	0	0	0	0,7
2016	26	0	0	0	0	0	0
2016	26	0	0	0	0	0	1,5
2016	26	0	0	0	0	0	0
2016	26	0	0	0	0	0	0,8
2016	26	0	0	0	0	0	7,4
2016	26	0	0	0	0	0	0,8
2016	26	0	0	0	0	0	10,1
2016	26	0	0	0	0	0	1,6
2016	26	0	0	0	0	0	0
2016	26	0	0	0	0	0	0,6
2016	21	0	0	0	0	0	0
2016	21	0	0	0	0	0	0,8
2016	21	0	0	0	0	0	1
2016	21	0	0	0	0	0	1,6
2016	21	0	0	0	0	0	0
2016	21	0	2	1	0	0	1,2
2016	21	0	2	0	1	0	0
2016	21	0	0	0	0	0	0
2016	21	0	0	0	0	0	0
2016	21	0	0	0	0	0	0,9
2016	15	0	0	0	0	0	0
2016	15	0	0	0	0	0	0
2016	15	1	1	1	0	0	54,7
2016	15	1	0	0	0	0	80
2016	15	1	0	0	0	0	37,1
2016	15	0	0	0	0	0	5
2016	15	0	0	0	0	0	0
2016	15	0	0	0	0	0	1
2016	15	1	0	0	0	0	17,7
2016	15	0	0	0	0	0	3,7
2016	43	0	0	0	0	0	0,9
2016	43	0	0	0	0	0	0
2016	43	0	0	0	0	0	0
2016	43	0	0	0	0	0	0,8
2016	43	0	0	0	0	0	0
2016	43	0	0	0	0	0	1,7

2016	43	0	0	0	0	0	0,6
2016	43	0	0	0	0	0	1,6
2016	43	0	0	0	0	0	0
2016	43	0	0	0	0	0	0
2016	17	0	0	0	0	0	0
2016	17	0	0	0	0	0	1,7
2016	17	0	0	0	0	0	1,6
2016	17	0	0	0	0	0	0
2016	17	0	0	0	0	0	0
2016	17	0	0	0	0	0	0
2016	17	0	0	0	0	0	0
2016	17	0	0	0	0	0	0
2016	17	0	0	0	0	0	6,3
2016	17	0	0	0	0	0	2,7
2016	19	0	2	0	1	0	0
2016	19	0	2	0	1	0	0
2016	19	0	0	0	0	0	0
2016	19	0	0	0	0	0	2,7
2016	19	0	2	0	1	0	0
2016	19	0	0	0	0	0	0
2016	19	0	0	0	0	0	0
2016	19	0	0	0	0	0	4,1
2016	19	0	2	0	1	0	2
2016	19	0	0	0	0	0	0
2017	33	0	1	0	1	0	0,6
2017	33	0	0	0	0	0	0
2017	33	0	0	0	0	0	0,8
2017	33	1	0	0	0	0	1,5
2017	33	0	1	0	1	0	0
2017	33	0	0	0	0	0	6,4
2017	33	0	0	0	0	0	0
2017	33	0	2	0	1	0	1,6
2017	33	0	0	0	0	0	0
2017	33	0	0	0	0	0	0
2017	49	0	0	0	0	0	1,2
2017	49	0	0	0	0	0	5,1
2017	49	1	0	0	0	0	6,1
2017	49	0	0	0	0	0	6
2017	49	0	1	0	1	0	0
2017	49	0	0	0	0	0	5
2017	49	1	0	0	0	0	25,8
2017	49	0	0	0	0	0	2,6
2017	49	0	0	0	0	0	5,6
2017	49	0	0	0	0	0	3,6
2017	13	0	0	0	0	0	0
2017	13	0	0	0	0	0	0,7
2017	13	0	2	1	0	0	1,8
2017	13	0	0	0	0	0	2
2017	13	0	0	0	0	0	0
2017	13	0	0	0	0	0	0
2017	13	0	0	0	0	0	1,3
2017	13	0	0	0	0	0	1,4
2017	13	0	0	0	0	0	0

2017	13	0	0	0	0	0	0,7
2017	14	0	0	0	0	0	0
2017	14	0	0	0	0	0	0,8
2017	14	0	0	0	0	0	2,3
2017	14	0	0	0	0	0	0
2017	14	0	0	0	0	0	0,7
2017	14	0	0	0	0	0	0
2017	14	0	0	0	0	0	2,3
2017	14	0	0	0	0	0	2,8
2017	14	0	0	0	0	0	0
2017	14	0	0	0	0	0	2,9
2017	56	0	0	0	0	0	5,3
2017	56	1	0	0	0	0	34,7
2017	56	1	2	0	0	1	7,9
2017	56	0	0	0	0	0	6,7
2017	56	0	0	0	0	0	2,9
2017	56	1	0	0	0	0	6,4
2017	56	0	0	0	0	0	20,2
2017	56	1	0	0	0	0	0,9
2017	56	0	0	0	0	0	8,4
2017	56	0	0	0	0	0	0
2017	57	0	0	0	0	0	0
2017	57	0	0	0	0	0	0,6
2017	57	0	0	0	0	0	0
2017	57	0	0	0	0	0	0
2017	57	0	1	0	1	0	0
2017	57	0	0	0	0	0	0
2017	57	0	0	0	0	0	0
2017	57	0	0	0	0	0	0
2017	57	0	0	0	0	0	0
2017	57	0	0	0	0	0	0
2017	44	1	0	0	0	0	7,3
2017	44	0	1	0	1	0	1,9
2017	44	0	0	0	0	0	0
2017	44	0	0	0	0	0	1,9
2017	44	0	1	0	1	0	7,3
2017	44	0	0	0	0	0	2,2
2017	44	0	0	0	0	0	2,8
2017	44	0	0	0	0	0	12,3
2017	44	0	0	0	0	0	0
2017	44	0	0	0	0	0	0
2017	58	1	3	0	1	0	0
2017	58	0	0	0	0	0	0,7
2017	58	0	3	0	1	0	0
2017	58	0	1	0	1	0	0,9
2017	58	1	1	0	1	0	0
2017	58	0	2	0	1	0	0
2017	58	0	2	0	1	0	0
2017	58	0	1	0	1	0	0
2017	58	0	2	0	1	0	0,7
2017	58	0	1	0	1	0	0
2017	51	0	0	0	0	0	0
2017	51	0	0	0	0	0	0

2017	51	0	0	0	0	0	0
2017	51	0	0	0	0	0	0,7
2017	51	0	0	0	0	0	0
2017	51	0	0	0	0	0	0
2017	51	1	0	0	0	0	1
2017	51	0	0	0	0	0	0
2017	51	0	0	0	0	0	0
2017	51	0	0	0	0	0	2,1
2017	27	0	0	0	0	0	4,5
2017	27	1	0	0	0	0	13,6
2017	27	0	0	0	0	0	3,2
2017	27	1	1	0	1	0	13,6
2017	27	1	0	0	0	0	12,2
2017	27	0	0	0	0	0	2,3
2017	27	0	0	0	0	0	2,3
2017	27	0	0	0	0	0	3,2
2017	27	1	0	0	0	0	0,8
2017	27	1	1	0	1	0	5,2
2017	47	0	0	0	0	0	1
2017	47	0	0	0	0	0	1,5
2017	47	0	0	0	0	0	0
2017	47	0	0	0	0	0	1
2017	47	0	0	0	0	0	0,9
2017	47	0	0	0	0	0	3,6
2017	47	0	0	0	0	0	0
2017	47	0	0	0	0	0	2,4
2017	47	0	0	0	0	0	5,5
2017	47	0	0	0	0	0	4,3
2017	30	0	0	0	0	0	1,3
2017	30	1	0	0	0	0	2,3
2017	30	0	0	0	0	0	2,3
2017	30	0	0	0	0	0	2,8
2017	30	0	0	0	0	0	0
2017	30	0	0	0	0	0	4,3
2017	30	0	0	0	0	0	1,7
2017	30	0	1	0	1	0	5,1
2017	30	1	0	0	0	0	7,8
2017	30	0	0	0	0	0	3,2
2017	3	0	0	0	0	0	8,1
2017	3	0	0	0	0	0	3,7
2017	3	1	1	0	1	0	1
2017	3	0	0	0	0	0	5,8
2017	3	0	1	0	1	0	4,2
2017	3	1	0	0	0	0	0,9
2017	3	1	0	0	0	0	13,9
2017	3	0	1	0	1	0	5,8
2017	3	1	1	0	1	0	10,3
2017	3	0	2	0	1	0	16,4
2017	60	0	0	0	0	0	0,7
2017	60	0	0	0	0	0	0
2017	60	0	0	0	0	0	0,8
2017	60	0	0	0	0	0	1,8
2017	60	0	0	0	0	0	0

2017	60	0	0	0	0	0	0,7
2017	60	0	0	0	0	0	3,7
2017	60	0	0	0	0	0	2,7
2017	60	0	0	0	0	0	0,7
2017	60	0	0	0	0	0	0
2017	28	1	0	0	0	0	2,1
2017	28	1	0	0	0	0	5,4
2017	28	1	0	0	0	0	1,4
2017	28	0	0	0	0	0	1,7
2017	28	0	0	0	0	0	2
2017	28	0	0	0	0	0	1,9
2017	28	1	0	0	0	0	1,9
2017	28	1	0	0	0	0	1,6
2017	28	1	0	0	0	0	8
2017	28	1	1	0	1	0	0
2017	55	1	1	0	1	0	1,2
2017	55	0	1	0	1	0	0
2017	55	0	0	0	0	0	2,2
2017	55	0	0	0	0	0	0
2017	55	0	1	0	1	0	0
2017	55	0	0	0	0	0	0
2017	55	0	0	0	0	0	0
2017	55	0	0	0	0	0	0
2017	55	0	0	0	0	0	0,8
2017	55	1	2	0	1	0	1,6
2017	52	0	0	0	0	0	0
2017	52	0	0	0	0	0	11,5
2017	52	0	0	0	0	0	3,5
2017	52	0	1	0	1	0	0
2017	52	0	0	0	0	0	8,3
2017	52	0	0	0	0	0	0
2017	52	0	0	0	0	0	4,5
2017	52	0	0	0	0	0	4
2017	52	1	0	0	0	0	4,4
2017	52	0	0	0	0	0	0
2017	59	0	0	0	0	0	3
2017	59	0	0	0	0	0	1,7
2017	59	0	0	0	0	0	0
2017	59	0	0	0	0	0	2,2
2017	59	0	0	0	0	0	0
2017	59	0	0	0	0	0	0
2017	59	0	0	0	0	0	2,1
2017	59	0	0	0	0	0	0
2017	59	0	0	0	0	0	0
2017	59	0	0	0	0	0	1,3
2017	42	0	0	0	0	0	1,2
2017	42	0	0	0	0	0	6,8
2017	42	0	2	0	1	0	0
2017	42	0	0	0	0	0	1,5
2017	42	0	0	0	0	0	0
2017	42	0	0	0	0	0	2
2017	42	0	0	0	0	0	1,4
2017	42	0	0	0	0	0	3

2017	42	0	0	0	0	0	0,7
2017	42	0	0	0	0	0	0
2017	8	0	0	0	0	0	0
2017	8	0	0	0	0	0	1,3
2017	8	0	0	0	0	0	0,7
2017	8	0	0	0	0	0	0
2017	8	0	0	0	0	0	3,3
2017	8	0	0	0	0	0	0
2017	8	1	0	0	0	0	2,3
2017	8	0	0	0	0	0	0
2017	8	0	0	0	0	0	0
2017	8	0	0	0	0	0	0,7
2017	26	1	0	0	0	0	0,9
2017	26	1	0	0	0	0	0,7
2017	26	0	1	1	0	0	6,6
2017	26	0	0	0	0	0	0
2017	26	0	0	0	0	0	4,3
2017	26	0	1	1	0	0	1
2017	26	0	0	0	0	0	5
2017	26	0	0	0	0	0	11,8
2017	26	0	0	0	0	0	2,4
2017	26	0	0	0	0	0	0,9
2017	21	0	0	0	0	0	0,8
2017	21	0	0	0	0	0	0
2017	21	0	0	0	0	0	0
2017	21	0	0	0	0	0	0
2017	21	0	0	0	0	0	0
2017	21	0	0	0	0	0	0
2017	21	0	0	0	0	0	1,4
2017	21	0	1	1	0	0	0
2017	21	0	0	0	0	0	2,1
2017	21	0	1	1	0	0	0
2017	15	0	0	0	0	0	0
2017	15	1	3	0	0	1	7,1
2017	15	0	0	0	0	0	2,1
2017	15	0	0	0	0	0	7
2017	15	0	0	0	0	0	1,9
2017	15	1	1	1	0	0	0
2017	15	0	0	0	0	0	0,7
2017	15	0	2	0	0	1	1,4
2017	15	0	0	0	0	0	0,7
2017	15	0	1	1	0	0	1,5
2017	17	0	0	0	0	0	4,5
2017	17	0	0	0	0	0	0
2017	17	0	0	0	0	0	1,8
2017	17	1	0	0	0	0	2,3
2017	17	0	0	0	0	0	2,7
2017	17	0	0	0	0	0	5,8
2017	17	0	0	0	0	0	4,4
2017	17	1	0	0	0	0	21,4
2017	17	1	0	0	0	0	27,9
2017	17	0	0	0	0	0	7,9
2017	19	0	0	0	0	0	1

2017	19	0	0	0	0	0	0
2017	19	0	0	0	0	0	0,7
2017	19	1	2	0	1	0	0
2017	19	0	2	0	1	0	0
2017	19	0	0	0	0	0	0
2017	19	0	2	0	1	0	2,4
2017	19	0	0	0	0	0	0
2017	19	0	0	0	0	0	0,7
2017	19	0	0	0	0	0	0
2018	33	1	0	0	0	0	11,9
2018	33	1	0	0	0	0	6,5
2018	33	0	0	0	0	0	1,8
2018	33	1	0	0	0	0	4,5
2018	33	1	0	0	0	0	16,6
2018	33	1	0	0	0	0	53,1
2018	33	1	0	0	0	0	2,8
2018	33	0	0	0	0	0	5,6
2018	33	1	0	0	0	0	18
2018	33	1	0	0	0	0	9,9
2018	49	1	0	0	0	0	21,1
2018	49	1	0	0	0	0	51,6
2018	49	1	0	0	0	0	59,3
2018	49	1	0	0	0	0	124,7
2018	49	1	0	0	0	0	53,8
2018	49	1	0	0	0	0	117,9
2018	49	1	0	0	0	0	35,5
2018	13	1	3	0	0	1	2,5
2018	13	0	0	0	0	0	1,7
2018	13	0	0	0	0	0	1,4
2018	13	0	0	0	0	0	2,4
2018	13	0	0	0	0	0	3,3
2018	13	0	0	0	0	0	1,6
2018	13	0	0	0	0	0	0,8
2018	13	0	0	0	0	0	0,6
2018	13	0	0	0	0	0	5,4
2018	13	0	0	0	0	0	0,7
2018	14	0	0	0	0	0	3,9
2018	14	0	0	0	0	0	9,6
2018	14	0	0	0	0	0	3,3
2018	14	0	0	0	0	0	0
2018	14	0	0	0	0	0	1,3
2018	14	0	0	0	0	0	13,6
2018	14	0	0	0	0	0	8,6
2018	14	0	0	0	0	0	13,5
2018	14	1	0	0	0	0	1,8
2018	14	0	0	0	0	0	3,2
2018	56	0	0	0	0	0	1,8
2018	56	0	0	0	0	0	7,8
2018	56	0	0	0	0	0	5
2018	56	0	0	0	0	0	5,5
2018	56	1	0	0	0	0	14,6
2018	56	0	0	0	0	0	0,8
2018	56	0	0	0	0	0	1,7

2018	56	0	0	0	0	0	1,4
2018	56	0	0	0	0	0	1,2
2018	56	0	0	0	0	0	2,5
2018	57	0	0	0	0	0	23,5
2018	57	0	0	0	0	0	5,7
2018	57	0	0	0	0	0	9,9
2018	57	0	0	0	0	0	9,1
2018	57	0	0	0	0	0	9,2
2018	57	0	0	0	0	0	19,4
2018	57	1	0	0	0	0	32,3
2018	57	0	0	0	0	0	60,8
2018	57	1	0	0	0	0	17,9
2018	57	1	0	0	0	0	34,8
2018	44	0	0	0	0	0	0
2018	44	0	0	0	0	0	0
2018	44	0	0	0	0	0	0
2018	44	0	0	0	0	0	0
2018	44	0	0	0	0	0	0,6
2018	44	0	0	0	0	0	0
2018	44	1	0	0	0	0	0
2018	44	0	0	0	0	0	0
2018	58	0	0	0	0	0	5,8
2018	58	0	0	0	0	0	1,9
2018	58	0	0	0	0	0	3,3
2018	58	0	0	0	0	0	13,9
2018	58	0	0	0	0	0	0,7
2018	58	0	0	0	0	0	20,1
2018	58	0	0	0	0	0	8
2018	58	0	0	0	0	0	5,1
2018	58	0	0	0	0	0	17,4
2018	58	0	0	0	0	0	1,1
2018	47	0	0	0	0	0	1,2
2018	47	0	0	0	0	0	0,6
2018	47	1	0	0	0	0	0,7
2018	47	0	0	0	0	0	0
2018	47	1	0	0	0	0	25,6
2018	47	0	0	0	0	0	6,4
2018	47	0	0	0	0	0	3
2018	47	0	0	0	0	0	0
2018	47	0	0	0	0	0	1,4
2018	47	0	0	0	0	0	3,7
2018	30	0	0	0	0	0	0
2018	30	1	0	0	0	0	11,5
2018	30	0	0	0	0	0	0,6
2018	30	0	0	0	0	0	0
2018	30	0	2	0	1	0	0
2018	30	0	0	0	0	0	0,6
2018	30	0	0	0	0	0	0,6
2018	30	0	0	0	0	0	0,6
2018	30	0	0	0	0	0	1,1
2018	30	0	0	0	0	0	1,8
2018	3	0	0	0	0	0	0,6
2018	3	0	0	0	0	0	1,5

2018	3	0	0	0	0	0	3,4
2018	3	0	0	0	0	0	5,8
2018	3	0	0	0	0	0	4,5
2018	3	0	0	0	0	0	5,7
2018	3	0	0	0	0	0	1
2018	3	0	0	0	0	0	1,7
2018	3	0	0	0	0	0	0,7
2018	3	0	0	0	0	0	0
2018	60	0	0	0	0	0	2,3
2018	60	0	0	0	0	0	32,8
2018	60	0	0	0	0	0	0
2018	60	0	0	0	0	0	6,2
2018	60	0	2	0	1	0	0
2018	60	0	0	0	0	0	33,8
2018	60	0	0	0	0	0	3,9
2018	60	0	0	0	0	0	0
2018	60	0	0	0	0	0	0
2018	60	0	0	0	0	0	0
2018	28	0	0	0	0	0	0,7
2018	28	0	0	0	0	0	3,3
2018	28	0	0	0	0	0	1,2
2018	28	0	0	0	0	0	1,1
2018	28	0	0	0	0	0	0,6
2018	28	0	0	0	0	0	0,7
2018	28	0	0	0	0	0	0
2018	28	0	0	0	0	0	0
2018	28	0	0	0	0	0	0
2018	28	0	0	0	0	0	8,3
2018	55	0	0	0	0	0	0,7
2018	55	0	0	0	0	0	4,2
2018	55	0	0	0	0	0	0,7
2018	55	0	0	0	0	0	0
2018	55	0	0	0	0	0	0
2018	55	0	0	0	0	0	0
2018	55	0	0	0	0	0	3,6
2018	55	0	1	0	1	0	1,3
2018	55	0	0	0	0	0	0,7
2018	55	0	0	0	0	0	0
2018	52	0	0	0	0	0	0
2018	52	0	0	0	0	0	0
2018	52	0	0	0	0	0	0,6
2018	52	0	0	0	0	0	0
2018	52	0	0	0	0	0	0
2018	52	0	0	0	0	0	0
2018	52	0	0	0	0	0	0
2018	52	0	0	0	0	0	0
2018	52	0	0	0	0	0	0
2018	52	0	0	0	0	0	0
2018	59	0	0	0	0	0	0
2018	59	0	0	0	0	0	1,4
2018	59	0	0	0	0	0	0
2018	59	0	0	0	0	0	1,3
2018	59	0	0	0	0	0	0,6

2018	59	0	0	0	0	0	0,7
2018	59	0	0	0	0	0	2,8
2018	59	0	0	0	0	0	6,4
2018	59	0	0	0	0	0	0
2018	59	1	0	0	0	0	0
2018	61	0	0	0	0	0	0
2018	61	0	0	0	0	0	0,5
2018	61	0	0	0	0	0	4,7
2018	61	0	0	0	0	0	0
2018	61	0	0	0	0	0	0
2018	61	0	0	0	0	0	0
2018	61	0	0	0	0	0	0
2018	61	0	0	0	0	0	0,6
2018	61	0	0	0	0	0	0
2018	61	0	0	0	0	0	0
2018	42	0	0	0	0	0	7,7
2018	42	1	0	0	0	0	6,7
2018	42	1	0	0	0	0	6
2018	42	0	0	0	0	0	0
2018	42	0	0	0	0	0	1,5
2018	42	1	0	0	0	0	16,2
2018	42	0	0	0	0	0	4,8
2018	42	0	0	0	0	0	1,4
2018	42	1	0	0	0	0	0
2018	42	0	0	0	0	0	0
2018	8	0	0	0	0	0	0,6
2018	8	0	0	0	0	0	0,7
2018	8	0	0	0	0	0	0,6
2018	8	0	0	0	0	0	0,8
2018	8	0	0	0	0	0	11,8
2018	8	0	0	0	0	0	5,2
2018	8	0	0	0	0	0	0,6
2018	8	0	0	0	0	0	11,7
2018	8	0	0	0	0	0	0,6
2018	8	0	0	0	0	0	1,5
2018	21	0	0	0	0	0	1,2
2018	21	0	0	0	0	0	0
2018	21	0	0	0	0	0	0
2018	21	0	0	0	0	0	0
2018	21	0	0	0	0	0	1,8
2018	21	0	0	0	0	0	0,5
2018	21	0	0	0	0	0	0
2018	21	0	2	0	1	0	0
2018	21	0	2	1	0	0	1,9
2018	21	0	0	0	0	0	0
2018	15	1	2	1	0	0	9,5
2018	15	1	0	0	0	0	1,4
2018	15	0	0	0	0	0	0,7
2018	15	1	0	0	0	0	2,1
2018	15	1	1	1	0	0	6,6
2018	15	1	0	0	0	0	18,2
2018	15	1	0	0	0	0	9,3
2018	15	1	2	0	1	0	0,8

2018	15	1	2	0	1	0	8
2018	15	0	0	0	0	0	0
2018	17	0	0	0	0	0	2
2018	17	0	0	0	0	0	1,7
2018	17	0	0	0	0	0	5
2018	17	0	0	0	0	0	2,5
2018	17	0	0	0	0	0	0,7
2018	17	0	0	0	0	0	10,9
2018	17	1	0	0	0	0	1,2
2018	17	0	0	0	0	0	0,6
2018	17	0	0	0	0	0	0,6
2018	17	1	0	0	0	0	6,8
2018	62	0	0	0	0	0	0,7
2018	62	0	0	0	0	0	1,6
2018	62	0	0	0	0	0	0
2018	62	1	0	0	0	0	9
2018	62	0	0	0	0	0	1,7
2018	62	0	0	0	0	0	0
2018	62	0	0	0	0	0	0
2018	62	0	0	0	0	0	0
2018	62	1	0	0	0	0	0
2018	62	0	0	0	0	0	1,7
2018	19	0	0	0	0	0	0
2018	19	1	1	0	1	0	0,8
2018	19	0	0	0	0	0	0
2018	19	0	2	0	1	0	0,7
2018	19	1	1	0	1	0	0
2018	19	0	0	0	0	0	0,6
2018	19	0	0	0	0	0	0,6
2018	19	0	0	0	0	0	1,9
2018	19	0	0	0	0	0	1,5
2018	19	0	0	0	0	0	14,5
2019	33	1	0	0	0	0	9,2
2019	33	0	0	0	0	0	3,3
2019	33	0	0	0	0	0	1,7
2019	33	0	0	0	0	0	3,4
2019	33	0	0	0	0	0	8,6
2019	33	0	0	0	0	0	3,9
2019	33	0	0	0	0	0	4,4
2019	33	1	0	0	0	0	13,3
2019	33	1	0	0	0	0	0
2019	33	1	0	0	0	0	0
2019	49	0	0	0	0	0	8
2019	49	0	0	0	0	0	2,1
2019	49	0	0	0	0	0	6,3
2019	49	0	0	0	0	0	38,5
2019	49	1	0	0	0	0	0
2019	49	0	0	0	0	0	13,6
2019	49	0	0	0	0	0	17,2
2019	49	1	0	0	0	0	147,4
2019	49	1	0	0	0	0	9,9
2019	49	1	0	0	0	0	21
2019	13	0	0	0	0	0	0,6

2019	13	0	0	0	0	0	2,5
2019	13	0	0	0	0	0	1,6
2019	13	0	1	0	1	0	2,3
2019	13	0	0	0	0	0	4,6
2019	13	0	0	0	0	0	0
2019	13	0	1	1	0	0	0
2019	13	0	0	0	0	0	1,9
2019	13	1	0	0	0	0	12,3
2019	13	0	0	0	0	0	1,6
2019	14	1	0	0	0	0	1,3
2019	14	0	0	0	0	0	2,3
2019	14	1	0	0	0	0	15,4
2019	14	0	0	0	0	0	3,9
2019	14	1	0	0	0	0	9
2019	14	0	0	0	0	0	8,3
2019	14	0	0	0	0	0	1,4
2019	14	0	0	0	0	0	0
2019	14	0	0	0	0	0	2,8
2019	14	1	0	0	0	0	1,8
2019	56	0	0	0	0	0	0,6
2019	56	1	0	0	0	0	2,3
2019	56	1	0	0	0	0	1,9
2019	56	1	0	0	0	0	1,8
2019	56	1	0	0	0	0	3,6
2019	56	0	0	0	0	0	0,8
2019	56	1	0	0	0	0	12,2
2019	56	0	0	0	0	0	0,7
2019	56	1	0	0	0	0	10,6
2019	57	0	0	0	0	0	10,7
2019	57	0	0	0	0	0	11,1
2019	57	0	0	0	0	0	14,2
2019	57	0	0	0	0	0	5,1
2019	57	0	0	0	0	0	30,9
2019	57	0	0	0	0	0	26,2
2019	57	0	0	0	0	0	19,8
2019	57	0	0	0	0	0	15,8
2019	57	1	0	0	0	0	12,2
2019	57	0	0	0	0	0	21,2
2019	44	0	0	0	0	0	1,2
2019	44	1	0	0	0	0	1,8
2019	44	0	0	0	0	0	2,7
2019	44	1	0	0	0	0	8,7
2019	44	1	0	0	0	0	0
2019	44	0	0	0	0	0	0
2019	44	0	0	0	0	0	0
2019	44	1	0	0	0	0	8,9
2019	44	1	2	0	1	0	0
2019	58	0	0	0	0	0	0
2019	58	0	3	0	1	0	0
2019	58	0	2	0	1	0	3,6
2019	58	0	3	0	1	0	0,7
2019	58	0	2	0	1	0	0
2019	58	0	0	0	0	0	0

2019	58	0	0	0	0	0	1,4
2019	58	0	1	0	1	0	0
2019	58	0	0	0	0	0	0
2019	58	0	3	0	1	0	0
2019	47	0	1	0	1	0	27,1
2019	47	0	0	0	0	0	3,4
2019	47	0	0	0	0	0	17,9
2019	47	0	0	0	0	0	3
2019	47	0	0	0	0	0	0
2019	47	0	0	0	0	0	23,8
2019	47	1	0	0	0	0	2,4
2019	47	1	0	0	0	0	28,3
2019	47	1	0	0	0	0	21,1
2019	47	0	0	0	0	0	1,5
2019	30	0	0	0	0	0	6,1
2019	30	1	0	0	0	0	12,1
2019	30	0	0	0	0	0	9,7
2019	30	0	0	0	0	0	1,8
2019	30	1	0	0	0	0	5,8
2019	30	1	0	0	0	0	15,6
2019	30	1	0	0	0	0	34,4
2019	30	0	0	0	0	0	2,9
2019	30	0	0	0	0	0	2
2019	30	0	0	0	0	0	0,6
2019	3	1	0	0	0	0	2
2019	3	0	0	0	0	0	9,7
2019	3	0	0	0	0	0	6,5
2019	3	0	0	0	0	0	5,6
2019	3	0	0	0	0	0	4,8
2019	3	1	0	0	0	0	20
2019	3	0	0	0	0	0	6,8
2019	3	1	0	0	0	0	9,7
2019	3	0	2	0	1	0	3
2019	3	1	0	0	0	0	18,8
2019	60	0	0	0	0	0	1,1
2019	60	0	1	0	1	0	25
2019	60	0	1	0	1	0	3,8
2019	60	0	0	0	0	0	6,5
2019	60	0	0	0	0	0	14,5
2019	60	0	0	0	0	0	4,8
2019	60	0	0	0	0	0	4,7
2019	60	0	0	0	0	0	19,4
2019	60	0	0	0	0	0	2
2019	60	0	1	0	1	0	2,9
2019	28	0	0	0	0	0	4,9
2019	28	0	0	0	0	0	5
2019	28	0	0	0	0	0	1,9
2019	28	0	0	0	0	0	0
2019	28	0	0	0	0	0	2,8
2019	28	0	2	0	1	0	12,2
2019	28	0	0	0	0	0	0,8
2019	28	0	1	0	1	0	2,6
2019	28	1	0	0	0	0	16,7

2019	28	1	0	0	0	0	5,7
2019	55	0	0	0	0	0	1,7
2019	55	0	0	0	0	0	1,8
2019	55	0	0	0	0	0	3,6
2019	55	0	0	0	0	0	0
2019	55	1	0	0	0	0	0
2019	55	1	0	0	0	0	0
2019	55	1	0	0	0	0	0,7
2019	55	0	1	0	1	0	0
2019	55	0	1	0	1	0	0,8
2019	55	1	0	0	0	0	0
2019	52	0	2	0	0	1	0
2019	52	0	0	0	0	0	0,7
2019	52	0	0	0	0	0	0
2019	52	0	0	0	0	0	0,8
2019	52	0	0	0	0	0	0
2019	52	0	0	0	0	0	0
2019	52	0	0	0	0	0	0,7
2019	52	0	0	0	0	0	0
2019	52	0	2	0	1	0	0,7
2019	52	0	0	0	0	0	0
2019	63	0	0	0	0	0	1,6
2019	63	0	0	0	0	0	0,7
2019	63	0	0	0	0	0	2,9
2019	63	0	0	0	0	0	1,1
2019	63	0	0	0	0	0	0
2019	63	0	0	0	0	0	0
2019	63	0	0	0	0	0	0,9
2019	63	0	0	0	0	0	0
2019	63	0	0	0	0	0	0
2019	63	0	0	0	0	0	1,7
2019	59	0	0	0	0	0	1,4
2019	59	0	0	0	0	0	1,8
2019	59	0	0	0	0	0	0
2019	59	0	0	0	0	0	2,1
2019	59	0	0	0	0	0	0,8
2019	59	0	0	0	0	0	0
2019	59	0	0	0	0	0	1,9
2019	59	0	0	0	0	0	0
2019	59	1	0	0	0	0	0,7
2019	59	0	0	0	0	0	2,8
2019	61	0	0	0	0	0	5,8
2019	61	1	0	0	0	0	2
2019	61	0	0	0	0	0	1,9
2019	61	0	0	0	0	0	0
2019	61	0	0	0	0	0	0,9
2019	61	0	0	0	0	0	0
2019	61	0	0	0	0	0	4,3
2019	61	0	0	0	0	0	0
2019	61	0	0	0	0	0	0
2019	61	0	0	0	0	0	3
2019	42	1	0	0	0	0	4,5
2019	42	0	0	0	0	0	2,8

2019	42	1	0	0	0	0	6,6
2019	42	1	0	0	0	0	1,2
2019	42	1	0	0	0	0	8,5
2019	42	0	1	0	1	0	1,4
2019	42	1	0	0	0	0	0
2019	42	0	1	0	1	0	0,7
2019	42	0	0	0	0	0	25,4
2019	42	1	0	0	0	0	3
2019	54	0	0	0	0	0	0
2019	54	0	3	0	1	0	0
2019	54	0	2	0	1	0	0
2019	54	0	0	0	0	0	0,7
2019	54	0	0	0	0	0	0
2019	54	0	0	0	0	0	1,1
2019	54	0	1	0	1	0	3,1
2019	54	0	1	0	1	0	0,8
2019	54	0	0	0	0	0	0
2019	54	0	0	0	0	0	0,9
2019	64	0	0	0	0	0	0,8
2019	64	0	0	0	0	0	0
2019	64	0	0	0	0	0	0
2019	64	0	0	0	0	0	0
2019	64	0	0	0	0	0	0
2019	64	0	0	0	0	0	0
2019	64	0	0	0	0	0	4,1
2019	64	0	0	0	0	0	0,6
2019	64	0	0	0	0	0	0
2019	64	0	0	0	0	0	0
2019	15	1	0	0	0	0	0
2019	15	0	0	0	0	0	2,2
2019	15	0	0	0	0	0	0,8
2019	15	0	0	0	0	0	1
2019	15	0	0	0	0	0	3,2
2019	15	0	0	0	0	0	3,6
2019	15	1	0	0	0	0	4,6
2019	15	0	0	0	0	0	3,7
2019	15	0	0	0	0	0	2,3
2019	15	0	3	1	0	0	3,2
2019	17	0	1	0	1	0	2,2
2019	17	1	0	0	0	0	25,2
2019	17	0	0	0	0	0	4,3
2019	17	0	0	0	0	0	17,4
2019	17	1	0	0	0	0	3,9
2019	17	0	0	0	0	0	3,1
2019	17	0	0	0	0	0	5
2019	17	0	0	0	0	0	1,9
2019	17	1	0	0	0	0	1,3
2019	17	1	0	0	0	0	23,2
2019	62	0	0	0	0	0	0,6
2019	62	0	0	0	0	0	6,7
2019	62	0	0	0	0	0	0
2019	62	0	0	0	0	0	0
2019	62	0	0	0	0	0	0,6

2019	62	0	0	0	0	0	0
2019	62	0	0	0	0	0	2,5
2019	62	0	0	0	0	0	0
2019	62	0	0	0	0	0	0
2019	62	0	0	0	0	0	0
2019	19	0	0	0	0	0	3,8
2019	19	0	0	0	0	0	1,8
2019	19	0	0	0	0	0	2
2019	19	0	0	0	0	0	1,6
2019	19	0	0	0	0	0	1,4
2019	19	0	0	0	0	0	8,7
2019	19	0	0	0	0	0	3,4
2019	19	0	0	0	0	0	3
2019	19	0	0	0	0	0	7,3
2019	19	0	0	0	0	0	0,7

Notes: colony loss, 1=yes, 0=no; Nosema infection category, 0=no spores, 1=1-10 spores, 2=11-100 spores, 3= more than 100 spores; *N. apis*, 1=yes, 0=no; *N. ceranae*, 1=yes, 0=no; *Nosema* mix, 1=yes, 0=no
