# Supplementary material for: Significant, but not biologically relevant: Nosema ceranae infections and winter losses of honey bee colonies
Source: Commun Biol. 2023 Mar 1;6:229. doi: 10.1038/s42003-023-04587-7 (PMC9977864; doi:10.1038/s42003-023-04587-7)
Supplement: Supplementary file 2 — Reporting Summary [file 42003_2023_4587_MOESM2_ESM.pdf]

nature portfolio

Corresponding author(s): Elke Genersch

Last updated by author(s): Feb 1, 2023

Reporting Summary

Nature Portfolio wishes to improve the reproducibility of the work that we publish. This form provides structure for consistency and transparency in reporting. For further information on Nature Portfolio policies, see our Editorial Policies and the Editorial Policy Checklist.

Statistics

For all statistical analyses, confirm that the following items are present in the figure legend, table legend, main text, or Methods section.

n/a	Confirmed	
☐	☒	The exact sample size (*n*) for each experimental group/condition, given as a discrete number and unit of measurement
☐	☒	A statement on whether measurements were taken from distinct samples or whether the same sample was measured repeatedly
☐	☒	The statistical test(s) used AND whether they are one- or two-sided<br>*Only common tests should be described solely by name; describe more complex techniques in the Methods section.*
☐	☒	A description of all covariates tested
☐	☒	A description of any assumptions or corrections, such as tests of normality and adjustment for multiple comparisons
☐	☒	A full description of the statistical parameters including central tendency (e.g. means) or other basic estimates (e.g. regression coefficient) AND variation (e.g. standard deviation) or associated estimates of uncertainty (e.g. confidence intervals)
☐	☒	For null hypothesis testing, the test statistic (e.g. *F*, *t*, *r*) with confidence intervals, effect sizes, degrees of freedom and *P* value noted<br>*Give P values as exact values whenever suitable.*
☒	☐	For Bayesian analysis, information on the choice of priors and Markov chain Monte Carlo settings
☐	☒	For hierarchical and complex designs, identification of the appropriate level for tests and full reporting of outcomes
☐	☒	Estimates of effect sizes (e.g. Cohen's *d*, Pearson's *r*), indicating how they were calculated

Our web collection on statistics for biologists contains articles on many of the points above.

Software and code

Policy information about availability of computer code

Data collection	No software was used to collect the data.
Data analysis	No custom code or mathematical algorithms or software was used. Packages which were used for statistics in R are listed in the Statistical Analysis section:<br>Data was curated, transformed and presented in spreadsheets for the analysis with the statistic software R using the package openxlsx. For the dynamics of winter losses, mean mite infestation levels, and N. ceranae infection prevalence, we performed linear regression models using R base package stats. We constructed the decision tree with default settings using rpart and rattle. Two-way association plots were created by vcd. The map showing the sample collection sites and the boundaries of the Northeastern Federal States of Germany was sourced from the Database of Global Administrative Areas (http://GADM.org). Data on the location of the apiaries and the duration of participation were inserted with R by the use of the following packages: rnaturalearth, raster, ggplot2, sf, sp, rgeos, and reshape.

For manuscripts utilizing custom algorithms or software that are central to the research but not yet described in published literature, software must be made available to editors and reviewers. We strongly encourage code deposition in a community repository (e.g. GitHub). See the Nature Portfolio guidelines for submitting code & software for further information.

Data

Policy information about availability of data

All manuscripts must include a data availability statement. This statement should provide the following information, where applicable:

- Accession codes, unique identifiers, or web links for publicly available datasets
- A description of any restrictions on data availability
- For clinical datasets or third party data, please ensure that the statement adheres to our policy

All data generated or analyzed during this study are included in this published article (and its supplementary information files).

Human research participants

Policy information about studies involving human research participants and Sex and Gender in Research.

Reporting on sex and gender	The study did not involve human research participants.
Population characteristics	The study did not involve human research participants.
Recruitment	The study did not involve human research participants.
Ethics oversight	The study did not involve human research participants.

Note that full information on the approval of the study protocol must also be provided in the manuscript.

Field-specific reporting

Please select the one below that is the best fit for your research. If you are not sure, read the appropriate sections before making your selection.

☐ Life sciences ☐ Behavioural & social sciences ☒ Ecological, evolutionary & environmental sciences

For a reference copy of the document with all sections, see nature.com/documents/nr-reporting-summary-flat.pdf

Ecological, evolutionary & environmental sciences study design

All studies must disclose on these points even when the disclosure is negative.

Study description	The data set of this study comprises data and samples which were collected from autumn 2005 to spring 2020 in the course of a 15 year longitudinal cohort-study on Nosema spp. epidemiology and honey bee health. Honey bee samples were collected in autumn and colonies were checked for their survival in spring of the respective overwintering period (weeks 36 to week 14 of the following year) from about 23 apiaries which were located in Northeast-Germany. Briefly, apiaries participated with ten so called “monitoring colonies” each. Apiaries or monitoring colonies that dropped out during the study period were substituted by adequate replacement. Hence, more than half of the apiaries (14 of ~23) participated for more than 9 years and 5 of them even for the entire duration of the study, i.e. 15 years. From at least 19 apiaries, samples were provided over a time period of consecutive 5-11 years. This resulted in an annual mean of 23.4 ± 2.26 (mean ± SD) apiaries with 9.77 ± 1.25 (mean ± SD) colonies each, giving an overall count of n = 3502 sampled monitoring colonies which provide the basis of our analyses.
Research sample	Sampling of European honey bee (Apis mellifera) workers was performed essentially as already described. Briefly, between calendar week 36 and 38 (late September/ beginning of October), about 300 in-hive honey bees were sampled from a super above the queen excluder from each monitoring colony.
Sampling strategy	Sampling strategy and sample size were chosen according to Fries I, et al. Standard methods for Nosema research. J Apicult Res 52, http://dx.doi.org/10.3896/IBRA.3891.3852.3891.3814 (2013).
Data collection	In-hive honey bees were sampled from a super above the queen excluder from each monitoring colony in autumn by the study bee inspector during the autumn visit. Data on colony winter survival/mortality was collected in spring when the study bee inspector visited the apiary.
Timing and spatial scale	Collection of honey bee samples started in autumn 2005 and ended in autumn 2019, collection of data on winter mortality started in spring 2006 (for the winter season 2005/2006) and ended in spring 2020 (for the winter season 2019/2020). Samples were taken and data collected continuously from all monitoring colonies throughout the 15-year study period. Monitored apiaries were located in Northeast Germany as shown in a map given as Fig. 6 in the manuscript.
Data exclusions	No data were excluded.
Reproducibility	The data presented in this manuscript originate from a long-term monitoring study, not from an experimental study. In this sense the

Reproducibility	data are not "reproducible", but representative for the studied area. In addition, the duration of the study (15 years) rules out that the observed effects are single events or outliers.
Randomization	Since this is a monitoring study, collected samples were not allocated to different groups.
Blinding	Data collected on winter mortality of each colony each year and laboratory analysis of the sampled bees per colony and year were performed independently. Moreover, when the bee samples were analyzed in autumn, the status of the colony in spring (collapse or survival over winter) was not yet known.

Did the study involve field work? ☒ Yes ☐ No

Field work, collection and transport

Field conditions	Sampling of the bees was performed independently from field and environmental conditions. Sampling was always performed in a two-weeks period between calendar week 36 and 38 (late September/ beginning of October).
Location	Locations of sampling are given in Fig. 6 of the manuscript.
Access & import/export	No specific permissions or permits requested by law were required for access to the apiaries and hives and for collecting samples from the managed honey bee colonies other than the express consent of the beekeepers. The monitoring study did not involve any endangered or protected species.
Disturbance	The study did not cause any disturbances.

Reporting for specific materials, systems and methods

We require information from authors about some types of materials, experimental systems and methods used in many studies. Here, indicate whether each material, system or method listed is relevant to your study. If you are not sure if a list item applies to your research, read the appropriate section before selecting a response.

Materials & experimental systems

n/a	Involved in the study	
☒	☐	Antibodies
☒	☐	Eukaryotic cell lines
☒	☐	Palaeontology and archaeology
☐	☒	Animals and other organisms
☒	☐	Clinical data
☒	☐	Dual use research of concern

Methods

n/a	Involved in the study	
☒	☐	ChIP-seq
☒	☐	Flow cytometry
☒	☐	MRI-based neuroimaging

Animals and other research organisms

Policy information about studies involving animals; ARRIVE guidelines recommended for reporting animal research, and Sex and Gender in Research

Laboratory animals	This study does not involve laboratory animals.
Wild animals	The study did not involve wild animals or wild honey bees or free flying honey bees, but all samples were taken only from inside the managed honey bee colonies (...about 300 in-hive honey bees were sampled from a super above the queen excluder...).
Reporting on sex	Only female worker bees were collected because (i) male drones are only present in the summer season and hence, at the time of sampling in autumn, male drones are no longer present and (ii) the winter survival of a honey bee colony depends on the health of the female bees (one queen and thousands of workers) only.
Field-collected samples	Bee samples were stored upon collection at -20 °C and kept at -20 °C until further analysis.
Ethics oversight	For field studies involving Western honey bees originating from managed colonies, no ethical approval or guidance is required.

Note that full information on the approval of the study protocol must also be provided in the manuscript.
